# Local Geometry and Evolutionary Conservation of Protein Surfaces Reveal the Multiple Recognition Patches in Protein-Protein Interactions

**DOI:** 10.1371/journal.pcbi.1004580

**Published:** 2015-12-21

**Authors:** Elodie Laine, Alessandra Carbone

**Affiliations:** 1 Sorbonne Universités, UPMC-Univ P6, CNRS, Laboratoire de Biologie Computationnelle et Quantitative—UMR 7238, Paris, France; 2 Institut Universitaire de France, Paris, France; Tel Aviv University, ISRAEL

## Abstract

Protein-protein interactions (PPIs) are essential to all biological processes and they represent increasingly important therapeutic targets. Here, we present a new method for accurately predicting protein-protein interfaces, understanding their properties, origins and binding to multiple partners. Contrary to machine learning approaches, our method combines in a rational and very straightforward way three sequence- and structure-based descriptors of protein residues: evolutionary conservation, physico-chemical properties and local geometry. The implemented strategy yields very precise predictions for a wide range of protein-protein interfaces and discriminates them from small-molecule binding sites. Beyond its predictive power, the approach permits to dissect interaction surfaces and unravel their complexity. We show how the analysis of the predicted patches can foster new strategies for PPIs modulation and interaction surface redesign. The approach is implemented in JET^2^, an automated tool based on the Joint Evolutionary Trees (JET) method for sequence-based protein interface prediction. JET^2^ is freely available at www.lcqb.upmc.fr/JET2.

## Author Summary

Many questions regarding Protein-Protein Interactions (PPI) cannot be answered by just knowing the approximate location of the interaction site at the protein surface but demand an understanding of the geometrical organization of the interacting residues. For instance, one would like to estimate the number of interactions for a protein, identify precisely the borders of each interaction site possibly overlapping other sites, understand the structure and the usage of a moonlighting protein interaction site shared with several partners, identify the anchor points in an interaction site that allow for strong versus weak binding, identify the locations on a protein surface where artificial molecules (*e.g.* drugs) could best interfere with protein partners. To answer these questions, a detailed description of the interaction at the atomic level is needed and we present a novel computational approach, JET^2^, bringing insights on such a description. Beyond its highly precise predictive power, the approach permits to dissect the interaction surfaces and unravel their complexity. It fosters new strategies for protein-protein interactions modulation and interaction surface redesign.

This is a *PLOS Computational Biology* Methods paper.

## Introduction

Proteins regulate biological processes through a complex network of dynamical interactions. Protein-protein interactions (PPIs) are considered as increasingly important therapeutic targets [1–4]. However protein-protein interfaces are notably more difficult to characterize than typical drug design targets (*e.g.* small-molecule binding pockets). Numerous studies have described some structural properties of PPIs sites [5–13]. By analogy to the interior-surface dichotomy for protein structure folding, a core-rim dichotomy was proposed for protein-protein interfaces [14, 15]. The amino acids forming the interface core tend to be more hydrophobic than over the rim [14–17]; they are also more frequently hotspots [18] and, therefore, usually more conserved [19–23]. Starting from these observations, a formal structural definition of these regions was proposed and a new structural region, the support, was introduced [24]. An effort was also engaged to define multiple recognition patches in large protein interfaces [25].

Many questions regarding PPIs cannot be answered by just knowing the approximate location of the interaction site at the protein surface but demand an understanding of the geometrical organization of the interacting residues. For instance, one would like to estimate the number of interactions for a protein, identify precisely the borders of each interaction site possibly overlapping other sites, understand the structure and the usage of a moonlighting protein interaction site shared with several partners, identify the anchor points in an interaction site that allow for strong versus weak binding, identify the locations on a protein surface where artificial molecules (*e.g.* drugs) could best interfere with protein partners. To answer these questions, a detailed description of the interaction at the atomic level is needed and any computational tool bringing insights on such a description becomes extremely useful.

The challenge of understanding PPIs on the one hand, and, on the other, the knowledge accumulated on experimental protein interfaces, have stimulated a growing interest in the development of computational methods to predict protein-protein interfaces. Pioneering works relied on physico-chemical and geometric descriptors of protein structures [26], and on residue conservation [19, 27]. More recent methods [28–35] exploit diverse types of information—including sequence conservation, side-chain flexibility, secondary structures—and employ various algorithms—including neural networks, Bayesian networks, support vector machines. For instance the VORFFIP method [36] makes use of tens of descriptors and integrates them in a two-step random forest classifier. Other machine learning approaches, such as PredUs [37] and eFindSite^PPI^ [38], rely on the hypothesis that protein-protein interfaces are structurally conserved: they map experimentally characterized interfaces of structurally similar proteins onto the target protein. Although these machine learning approaches sometimes perform very well, they generally do not provide a clear understanding of the molecular determinants of protein-protein association.

We previously developed Joint Evolutionary Trees (JET) for protein interface prediction [39]. JET relies on the assumption that protein interfaces are composed of a core, formed by highly conserved residues having particular physico-chemical properties, and extends through concentric layers of gradually less conserved amino acids (S1 Fig). JET showed good performance on diverse reference data sets and compared favorably to other methods [39]. However our recent complete cross-docking study [40] highlighted the need for a very precise definition of the predicted binding sites to have discriminating power in evaluating docking poses of protein partners versus non-partners.

The present work revisits the idea formalized in JET, by defining a protein interface as formed by three structural regions, called seed, extension, and outer layer, that approximate the structural notions of support, core, and rim defined for experimental interfaces [24] (S1 Fig). Intuitively, protein interfaces are comprised of residues issued by a combination of conservation and/or physico-chemical properties (seed-extension/support-core) and extend through neighboring protruding regions (outer layer/rim). We propose this three-layer structure to be characteristic of protein-protein interfaces (in contrast to interfaces with other chemical compounds or biomolecules) and, based on it, we provide a large-scale predictive pipeline, called JET^2^. Our basic goal is to unravel the complexity of protein interaction surfaces by pinpointing characteristics that can define their structures and distinguish their multiple properties. We show that interaction sites can be described by very simple and general rules of organisation of the interacting residues, indicating that evolutionary constraints on this organisation exist and can be revealed.

JET^2^ is a new tool that takes as input the three-dimensional structure of a single protein and exploits both sequence and structural information, in contrast to JET. The strategy implemented in JET^2^ does not rely on learning and the method is computationally much simpler than any machine learning approach. We show that the use of a very straightforward geometric descriptor (circular variance) captures with remarkable precision the intrinsic geometry of protein-protein interfaces. Moreover different combinations of structural and sequence descriptors allow the detection of different protein surface regions and to deconstruct protein interfaces. We provide a completely versatile tool that enables the user to tune all parameters depending on the biological question one wants to answer. JET^2^ is freely available to the community and can be downloaded at www.lcqb.upmc.fr/JET2.

## Results

### Evolutionary, physico-chemical and structural signals encoded in experimental interfaces

**Fig 1:**
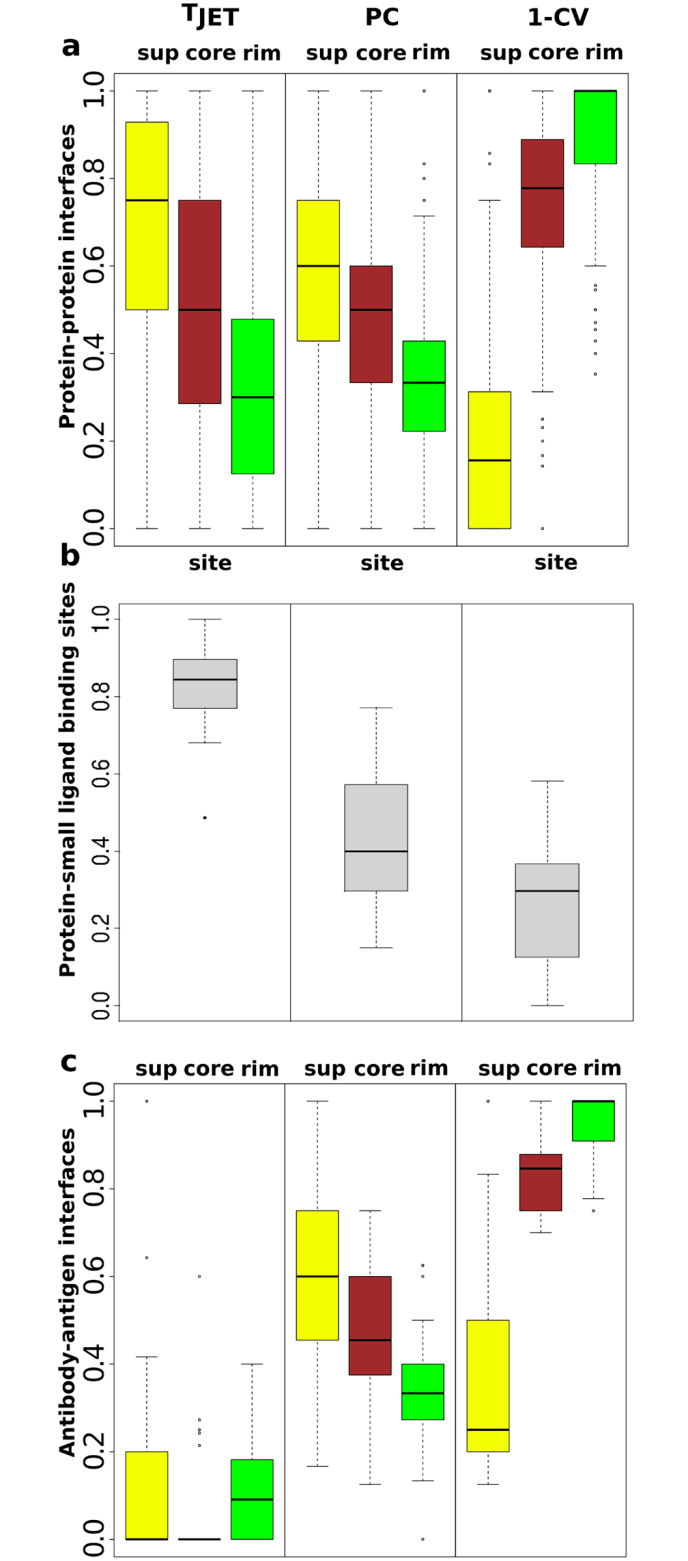
Signals detected in experimental interfaces. The boxplots represent the distributions of the proportions of interacting residues having values above the median value computed over the entire protein. T_*JET*_: conservation level, PC: interface propensity, CV: circular variance. Distributions are computed from PPDBv4 on: (**a**) all protein-protein interfaces, (**b**) all protein-small ligand binding sites, (**c**) protein-protein interfaces for antibodies. For protein-protein interfaces, the support, core and rim are in yellow, brown and green respectively.

**Fig 2:**
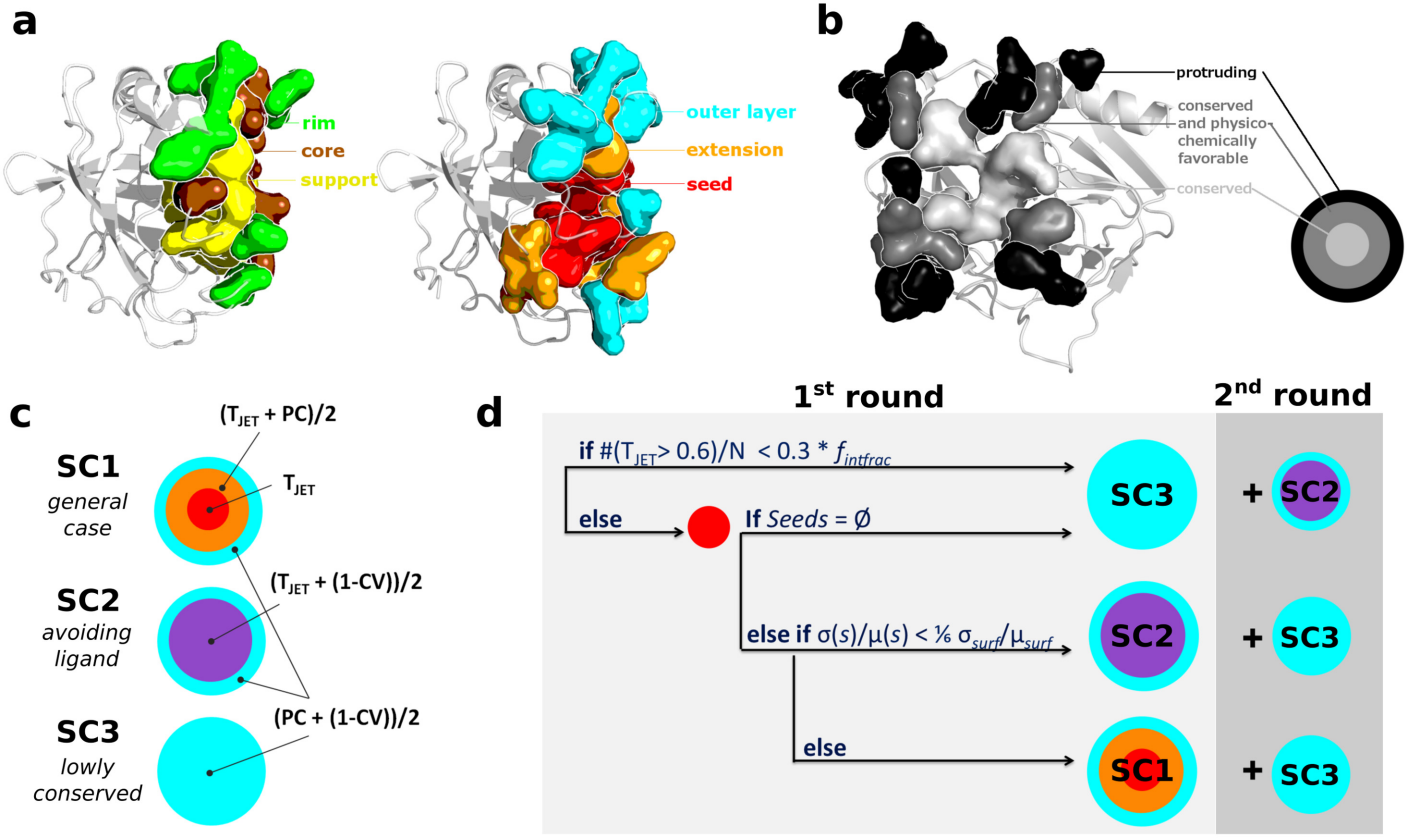
Experimental interface definition and JET^2^ scoring schemes. (**a**) Section of an experimental interface (on the left, PDB code: 1N8O) and of the corresponding JET^2^ prediction using **SC1** (on the right). The experimental and predicted interface residues are displayed in opaque surface: support, core and rim are in yellow, brown and green; cluster seed, extension and outer layer are in red, orange and cyan. (**b**) Front view of JET^2^ prediction illustrated in **a**. The cluster seed, extension and outer layer are colored in grey tones and labelled. (**c**) Schematic icons picturing JET^2^ three scoring schemes. T_*JET*_: conservation level, PC: interface propensity, CV: circular variance. Different colors correspond to different formulas used to detect the layers. (**d**) Schematic representation of the complete automated JET^2^ clustering procedure. In the first round, the implemented algorithm automatically chooses the most adequate scoring scheme depending on the studied system and detects a set of main clusters. The second round detects additional clusters, by using a complementary scoring scheme, which will be merged to the main clusters if sufficiently close to them (<5Å). JET^2^ clustering procedure can be run multiple times (iterative mode: iJET^2^) to get consensus predictions.

First, we analyzed experimentally determined protein complex structures to gain knowledge on protein-protein interfaces and identify their characteristic features. We computed the residue conservation levels (T_*JET*_), interface physico-chemical propensities (PC) and circular variances (CV) (see Materials and Methods) in the 176 protein complexes from the Protein-Protein Docking Benchmark version 4 (PPDBv4) [41] (S1 Table). The values obtained for the interacting residues were compared to those obtained for the rest of the protein (Fig 1A). Interacting residues are divided in support (the most buried, in yellow), core (intermediate, in brown) and rim (the most exposed, in green) [24] (Fig 2A, on the left; see Materials and Methods). T_*JET*_ distributions reveal that support residues are significantly more conserved than the rest of the protein whereas rim residues are significantly less conserved (Fig 1A). Core residues display intermediate profiles. PC values also decrease gradually from support through core to rim. For 1-CV, a striking contrast is observed between rim residues, that display a narrow distribution up to 1, indicating that they tend to be located in protruding protein regions, and support residues that have very low values. Overall, this analysis showed that different signals are encoded in the three different structural components of protein experimental interfaces.

Small ligand-binding sites generally form more concave and deeper cavities than protein-protein interfaces and those used by natural ligands are also expected to display strong conservation signals [42]. These characteristic features were verified on the 43 proteins from PPDBv4 that contain a bound small molecule (S2 Table). Interestingly, in most of those cases, the region of the protein surface targeted by the small ligand is close to or even overlaps with the protein-protein interface. The small ligand-binding site was defined as the set of residues located less than 5Å away from the small molecule (in grey). For T_*JET*_, a narrow distribution is observed around 0.84 while values for 1-CV are not higher than 0.58 (Fig 1B). These results support the assumption that small ligand-binding sites tend to be overall more conserved and more buried than protein-protein interfaces. Hence evolutionary information and geometric criteria could be used to distinguish the two types of binding sites.

We [40] and others [43, 44] have previously observed that antibody-antigen interfaces constitute particularly difficult cases for protein binding site prediction using evolutionary information. Fig 1C reports distributions for T_*JET*_, PC and 1-CV focusing on the 25 antibodies from PPDBv4. It is clear from the T_*JET*_ boxplots that the evolutionary signal that can be extracted from antibody interfaces is very poor. By contrast, PC and 1-CV distributions show trends similar to the other proteins from the dataset. This analysis revealed that evolutionary information may not be helpful for detecting protein binding sites in peculiar cases such as antibody-antigen interfaces.

### Modeling protein interfaces based on the geometry of the interactions

Based on our analysis of signals encoded in experimental protein interfaces, we defined a predictive model (Fig 2A, right; see Material and Methods and S3 Table) and we used it to detect putative PPI sites. In this model, we consider only surface residues and ignore completely or almost completely buried residues (*asa*<5%) that are generally highly conserved and would not contribute significantly to the interface (S3 Table). Predicted protein interfaces are defined as residue clusters composed of a *seed*, forming the core of the predicted site, several concentric layers forming an *extension*, and an additional *outer layer*. Intuitively, seed/extension/outer layer approximate support/core/rim, although we do not seek a perfect match between the individual components of the two models.

The variable nature of protein interfaces, represented in PPDBv4, emphasizes the need for adapted modeling approaches to correctly predict them. Consequently, we devised specific clustering strategies, aiming at detecting support, core and rim residues in a wide range of interfaces (Fig 2B). Namely, we developed three scoring schemes (**SC**) that combine evolutionary information from the sequence (T_*JET*_), amino-acid interface propensities (PC) and local geometry of the structure (CV), as follows (Fig 2C):

- **SC1** uses the three descriptors for defining the three components of the predictive model. Very conserved residues (T_*JET*_ only) are detected and grouped to form cluster seeds, which are then extended using T_*JET*_ and PC. An outer layer is finally added considering PC and CV. **SC1** is intended to detect diverse protein binding sites and thus corresponds to the general case.
- **SC2** uses a combination of T_*JET*_ and CV for seed detection and extension. This ensures that strong evolutionary signals are captured while avoiding residues too buried inside the protein. The outer layer is defined based on PC and CV, as in **SC1**. **SC2** was designed to specifically detect protein interfaces that are located close to or overlap with small-ligand binding pockets.
- **SC3** disregards evolutionary information and rather employs PC and CV for detecting all three components. The development of **SC3** was motivated by the observation that some protein interfaces, *e.g.* antigen-binding sites from PPDBv4, contain very low conservation signal to none. We expect **SC3** to yield consistent predictions for those difficult cases.

**Fig 3:**
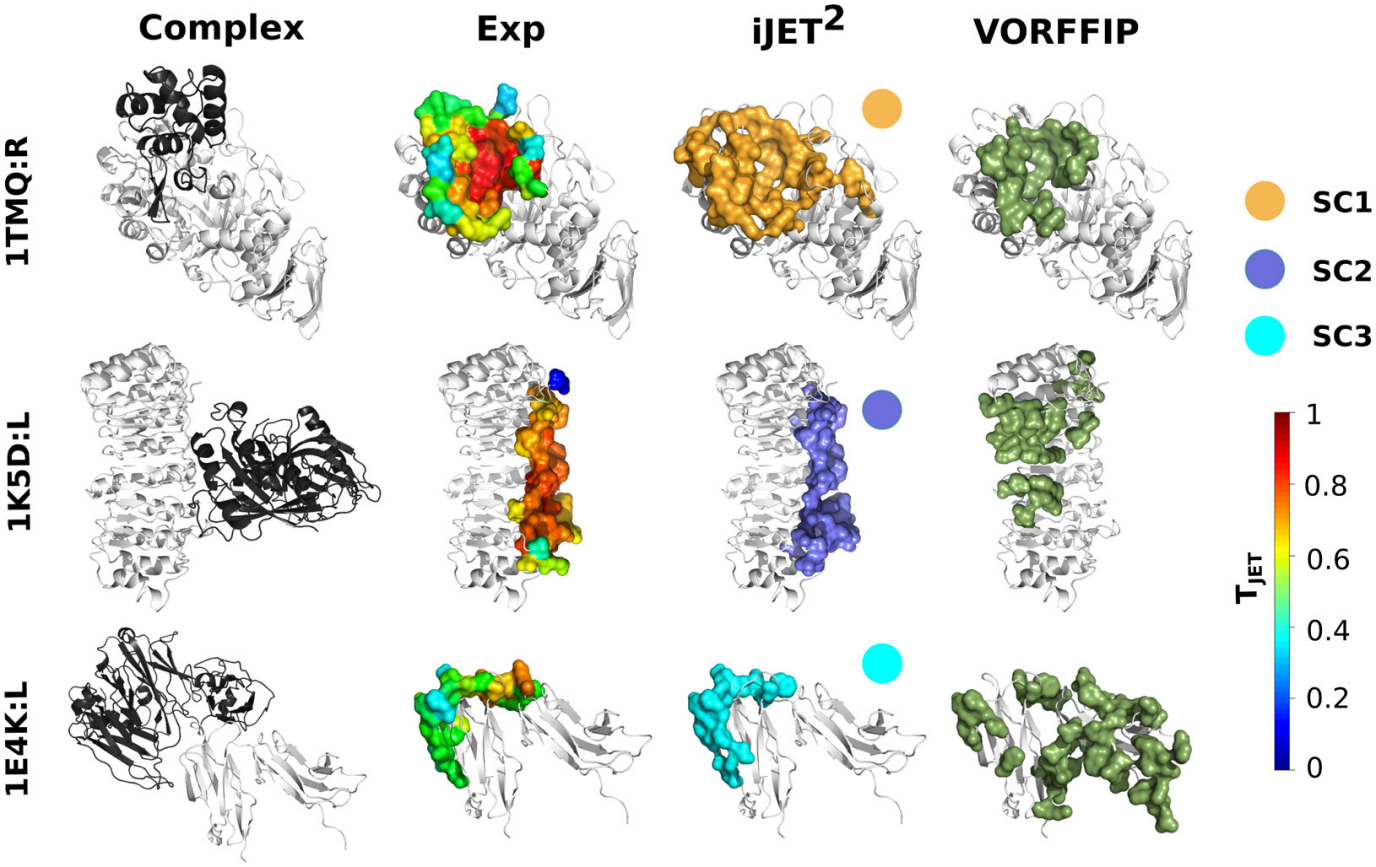
Examples of interaction sites predicted by the three scoring schemes. The experimental complexes formed between the proteins of interest (light grey) and their partners (dark grey) are represented as cartoons. The experimental and predicted binding sites are displayed as opaque surfaces. The experimental interface residues are colored according to T_*JET*_ values computed by iJET^2^. iJET^2^ predictions were obtained from a consensus of 2 runs out of 10. They are colored according to the scoring scheme from which they were obtained: **SC1** in orange, **SC2** in purple and **SC3** in cyan. The scoring schemes are indicated for each protein. They were automatically chosen by iJET^2^ clustering algorithm (first round). VORFFIP predictions are colored in dark green.

In each **SC**, T_*JET*_, PC and/or CV are combined in a very straightforward manner without any specific weight (S3 Table). Depending on the **SC** employed, the predicted patch may be highly conserved, display peculiar physico-chemical and/or geometrical properties. We developed an algorithm that determines the best strategy depending on the system studied (Fig 2D; see Materials and Methods). Examples of predictions are illustrated in Fig 3. They were obtained by running our tool JET^2^, that implements the fully automated clustering procedure. Let us stress that JET^2^ also allows the user to manually choose a particular scoring scheme. In the following we report JET^2^ performance on a number of different interfaces.

### Detecting lowly conserved interface residues based on the protein surface local geometry

To describe the local geometry of the protein surface, we use the measure of circular variance (CV) that evaluates the density of protein around an atom. This simple geometric descriptor captures the structural properties of interacting residues. To properly assess the predictive role of CV, we compared JET^2^ and its iterative version iJET^2^ (see Materials and Methods) to JET/iJET, which use only sequence information. We applied both methods to two testing sets, namely PPDBv4 and the Huang dataset of 62 protein complexes [20] (S1 Table). One should notice that PPDBv4 was also used for the analysis of the signals encoded in experimental interfaces (see above). Although this analysis conceptually inspired the detection strategies implemented in JET^2^, the method was not trained on PPDBv4 and no JET^2^ parameter was set based on PPDBv4 analysis. Hence, PPDBv4 could be used for assessing JET^2^ predictive power.

**Fig 4:**
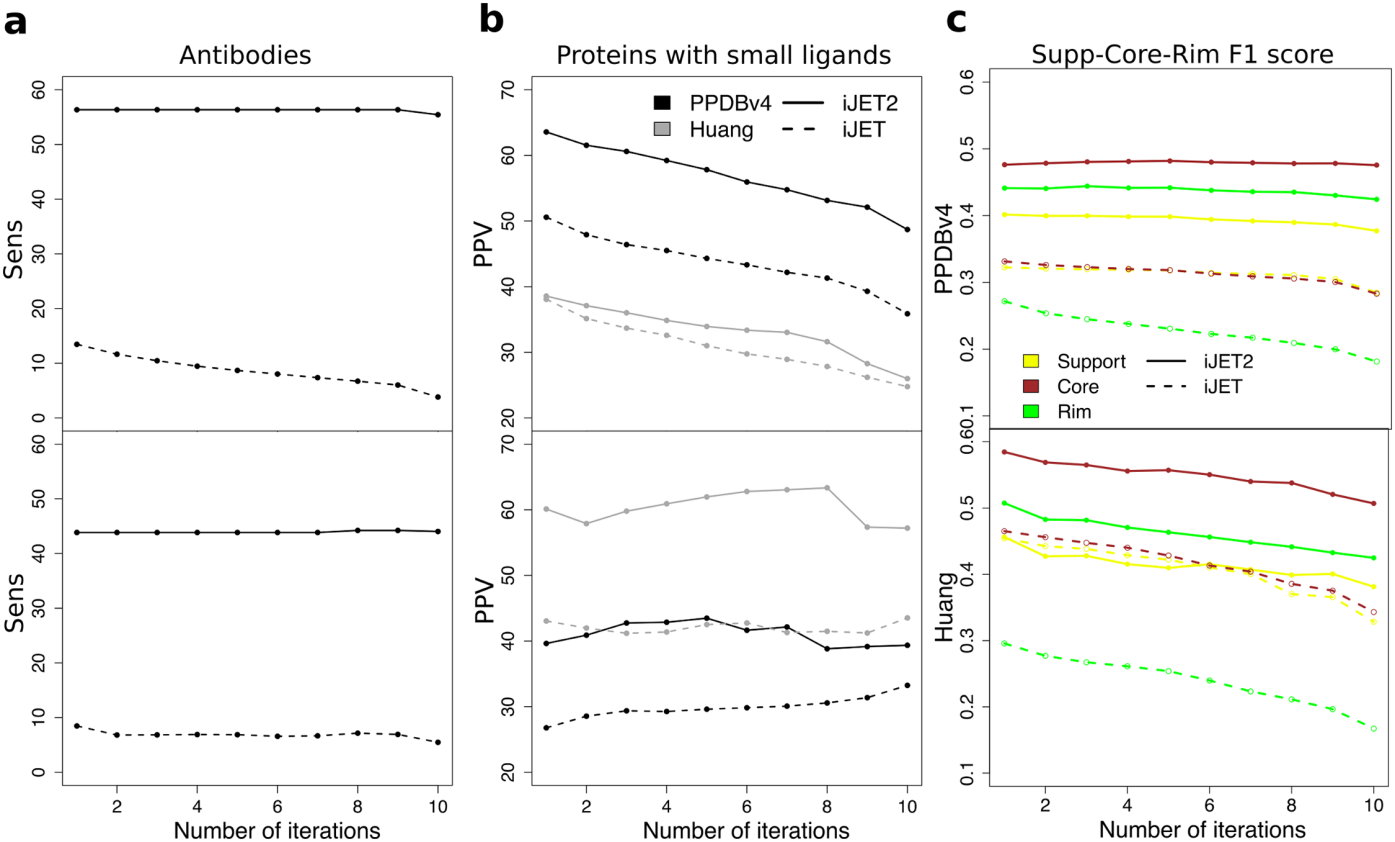
Performance of iJET^2^ on specific groups of proteins and comparison with iJET. (**a-b**) Sensitivity (top) and precision (bottom) average values computed on: (**a**) the antibodies (receptors) from PPDBv4, (**b**) the proteins containing a bound small molecule ligand, from PPDBv4 (in black) and Huang (in gray). (**c**) F1 score computed as the arithmetic mean of sensitivity and precision. The sensitivity is evaluated on the support (in yellow), core (in brown) or rim (in green) and the precision is evaluated on the whole interface. The values are averaged over all proteins from PPDBv4 (top) and Huang (bottom). Predictions were obtained by a consensus over 10 runs.

**Fig 5:**
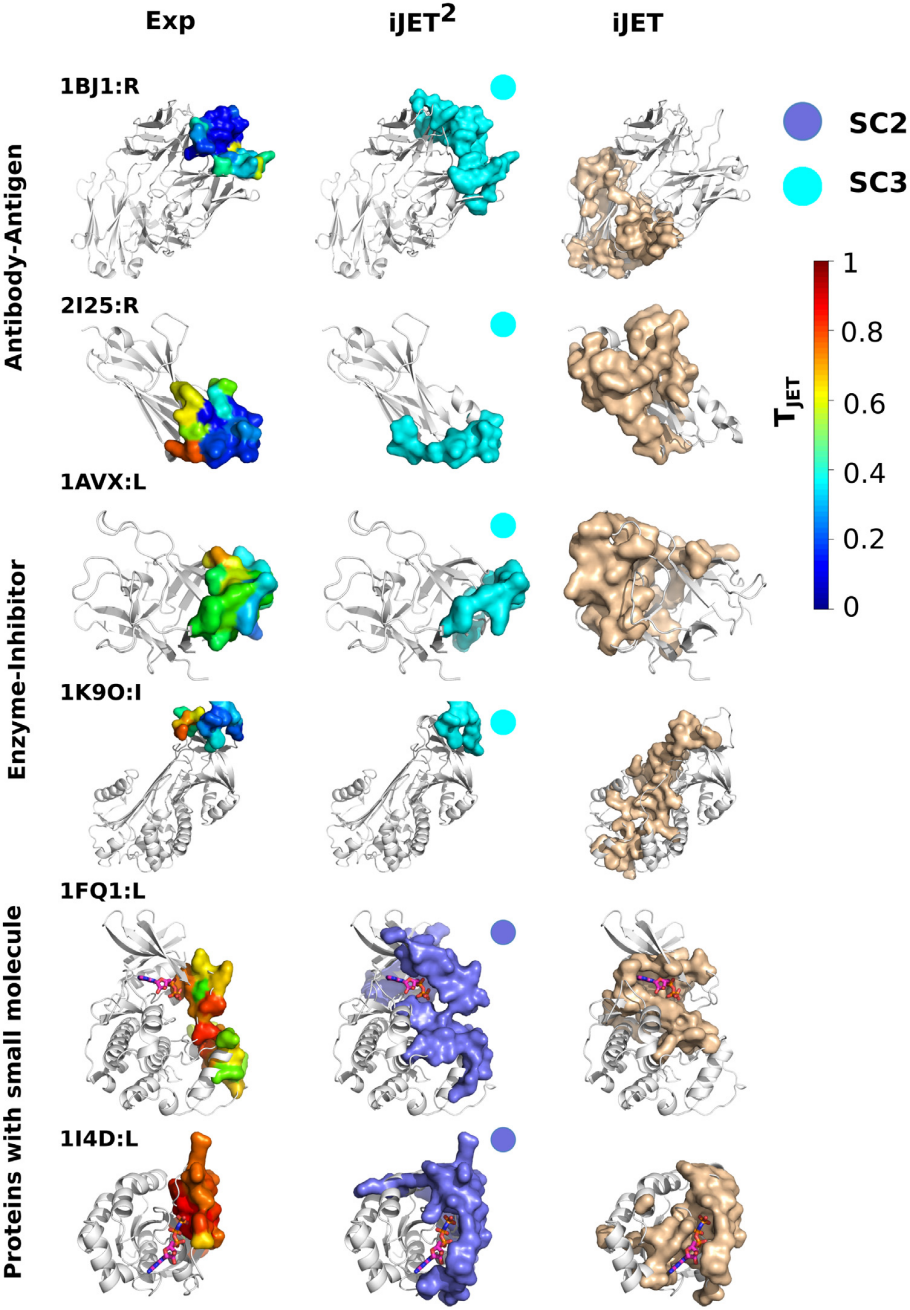
Single-patch interaction sites of groups of proteins known to be difficult to predict. The predicted and experimental binding sites are displayed as colored opaque surfaces. The experimental binding sites are colored according to T_*JET*_ computed by iJET^2^. iJET^2^ predictions were obtained from a consensus of 2 runs out of 10. They are colored according to the scoring scheme from which they were obtained: **SC2** in purple and **SC3** in cyan. The scoring schemes are indicated for each protein. A comparison with iJET predictions (right) is shown.

Lowly conserved interacting residues are found in the antigen-binding sites of the 25 antibodies from PPDBv4 (Fig 1C). JET^2^/iJET^2^ dramatically improves the detection of these interfaces compared to JET/iJET (Table 1 and Fig 4A). The scoring scheme **SC3** enables to increase sensitivity by almost 50 and precision by more than 30 for this group of proteins. The cases of the Fab fragment of an anti-VEGF antibody (1BJ1:R) and the shark new antigen receptor PBLA8 (2I25:R) illustrate the power of **SC3** in predicting with high precision (*PPV* = 72% and 60%) antigen-binding sites while iJET failed to correctly detect them (Fig 5).

**Table 1:** Comparison of JET^2^/iJET^2^, JET/iJET and VORFFIP performance.

	**Sen**	**ScSen**	**PPV**	**ScPPV**	**Spe**	**ScSpe**	**Acc**	**ScAcc**
**Antibodies (PPDBv4)**								
JET	8.41	-1.79	12.96	1.43	89.12	-0.68	82.94	4.23
iJET (7/10)	7.36	-4.54	6.66	0.58	87.21	-0.89	81.02	3.67
JET^2^	55.44	44.88	43.66	3.98	**93.05**	3.61	**89.52**	12.27
iJET^2^ (2/10)	**56.35**	**45.52**	**43.84**	**3.99**	92.85	**3.68**	89.43	**12.35**
**Proteins with bound small molecule**								
**PPDBv4**								
JET	42.99	26.86	32.09	1.41	87.75	3.89	82.75	14.33
iJET (7/10)	42.19	25.27	30.07	1.31	86.78	3.70	81.80	13.82
JET^2^	56.98	38.94	39.94	1.84	**87.27**	5.30	**83.86**	16.38
JET^2^ (2/10)	**61.55**	**41.48**	**40.89**	**1.89**	85.55	**5.62**	82.88	**16.48**
**Huang**								
JET	33.89	19.51	45.58	1.25	89.96	4.35	79.33	20.87
iJET (7/10)	28.92	14.95	41.30	1.13	89.17	3.14	77.47	19.37
JET^2^	33.51	21.63	**62.13**	**1.71**	**93.00**	4.88	**81.41**	**22.92**
iJET^2^ (2/10)	**37.11**	**23.02**	57.89	1.57	91.18	**5.27**	80.68	22.57
**Huang**								
**All**								
iJET (7/10)	31.96	15.12	49.32	1.14	88.68	5.52	75	20.37
iJET^2^ (2/10)	**47.92**	**26.39**	65.89	1.75	85.88	**7.41**	78.89	22.14
iJET^2^ (8/10)	41.69	25	**69.13**	**1.79**	**90.52**	7.21	**79.25**	**23.3**
${\text{iJET}}_{AutoComplete}^{2}$ (2/10)	43.43	22.33	55.02	1.40	84.64	5.74	76.30	19.88
${\text{iJET}}_{AutoComplete}^{2}$ (8/10)	34.37	18.31	57.26	1.47	88.53	4.59	76.44	19.89
All*								
iJET (7/10)	31.87	16.94	47.17	1.15	89.97	4.9	78.08	20.74
iJET^2^ (2/10)	46.6	28.74	66.18	1.9	89.27	7.14	81.65	23.06
iJET^2^ (8/10)	41.14	27.78	**69.87**	1.93	**93.62**	6.97	82.58	**24.14**
${\text{iJET}}_{AutoComplete}^{2}$ (2/10)	43.28	23.07	51.82	1.3	85.88	6.09	77.01	20.47
${\text{iJET}}_{AutoComplete}^{2}$ (8/10)	34.44	18.79	55.22	1.4	87.85	5.09	76.29	20.28
VORFFIP (>0.5)	**46.9**	**30.83**	68.34	**3.66**	92.39	**8.45**	**83.49**	12.13
**PPDBv4**								
**All**								
iJET (7/10)	34.56	14.54	28.79	1.06	81.39	3.12	76.46	11.58
iJET^2^ (2/10)	**58.34**	**32.77**	43.31	1.95	80.02	5.59	79.27	14.27
iJET^2^ (8/10)	52.83	31.3	**46.18**	**2.05**	**84.05**	**5.59**	**81.83**	**15.22**
${\text{iJET}}_{AutoComplete}^{2}$ (2/10)	54.16	25.17	33.64	1.47	75.20	4.19	74.84	11.34
${\text{iJET}}_{AutoComplete}^{2}$ (8/10)	44.34	22.01	34.30	1.48	79.78	3.82	76.81	12.17
All*								
iJET (7/10)	34.84	14.86	28.69	1.06	82.19	3.4	77.61	11.73
iJET^2^ (2/10)	**58.63**	**33.88**	43.51	2.11	80.87	5.62	80.2	14.21
iJET^2^ (8/10)	53.87	32.63	**45.7**	2.18	84.44	**5.68**	**82.54**	**15.11**
${\text{iJET}}_{AutoComplete}^{2}$ (2/10)	54.19	25.20	33.98	1.52	75.22	4.21	74.95	11.47
${\text{iJET}}_{AutoComplete}^{2}$ (8/10)	44.39	21.87	34.12	1.50	79.72	3.82	76.81	12.22
VORFFIP (p>0.5)	42.97	24.59	38.75	**3.24**	**86.09**	4.46	80.58	6.88

Statistical performance values are given in percentages. iJET^2^ (resp. iJET) predictions were obtained from a consensus of 2 or 8 (resp. 7) runs out of 10. For VORFFIP, predicted patches were defined as formed by residues with probability above 0.5. JET^2^/iJET^2^ were run in different modes. For the antibodies, **SC3** was manually chosen. For the proteins with bound small molecule, **SC2** was automatically selected by JET^2^ (26/34 proteins from PPDBv4, 19/20 from Huang) or manually chosen. For all proteins, the three scoring schemes were systematically used and the best patch or combination of patches was retained. The performance values obtained when running the complete automated clustering procedure of the program (${\text{JET}}_{AutoComplete}^{2}$) are also given.

In addition to antigen-binding sites, **SC3** detects 52% of the FCG-binding site of FCG receptor (1E4K:L) with a very high precision value of 86% (Fig 3). It also successfully predicts the binding sites of two trypsin inhibitors to their enzyme target, namely the Kunitz soybean trypsin inhibitor (1AVX:L, *Sens* = 55%, *PPV* = 69%) from PPDBv4 and alaserpin (1K9O:I, *Sens* = 79%, *PPV* = 48%) from Huang (Fig 5). By contrast, iJET could not detect any interacting residue of the former and predicted about 20% of the latter but with low precision (*PPV* = 24%).

Over all proteins, the most exposed interface residues, constituting the rim, are generally less conserved than the rest of the protein (Fig 1A). JET^2^ greatly improves the detection of these residues for both testing sets (Fig 4C). Sensitivity values obtained for the rim residues over 10 iterative runs of iJET^2^ are increased by 20 to 32 compared to iJET.

These results show that JET^2^, by exploiting the local geometry of the protein surface and combining it it with amino acids physico-chemical properties, is able to specifically detect lowly conserved protruding interacting residues, as those forming the rim of the interface or those involved in *e.g.* antigen-binding sites, which represent particularly difficult cases.

### Segregating protein- from small ligand-binding sites

We observed in PPDBv4 that small ligand-binding sites were often located in the vicinity of protein-binding sites or even overlapped with them. This can make the specific detection of protein-binding sites a difficult task. The ability of JET^2^/iJET^2^ to correctly detect protein-protein interfaces was evaluated on the 67 proteins—43 from PPDBv4 and 24 from Huang—that contain a bound small molecule (in the complexed conformational state). For 80% of these proteins, **SC2** enabled to get a more precise definition of the protein interface (Table 1 and Fig 4B). JET^2^/iJET^2^ predictions display improved precision by up to 17 and increased sensitivity by up to 20 on average compared to JET/iJET. Furthermore, in the large majority (83%) of these cases, JET^2^/iJET^2^ automated procedure successfully detected the presence of a small-ligand binding pocket and consequently chose **SC2** for protein interface prediction (Fig 2D; see Materials and Methods).

For example, iJET^2^ detects 60% and 79% of the residues involved in the protein-protein interfaces of adrenoxin reductase (1E6E:R) and the Ras-related protein Rab-33B (2G77:L) with precision values of 50% and 64% (Fig 5). By contrast, iJET patches are wrapped around the small molecule ligand.

Among the 67 analyzed proteins, 13 have a protein interface whose prediction is not improved when using **SC2**. This can be explained by three reasons: (1) the protein interface displays very low evolutionary signal, hence it is correctly detected using **SC3** (1ATN:R, 1RLB:L, 1XQS:L, 1WEJ:L from PPDBv4; 1QOR:A, 1RRP:C from Huang), (2) the protein interface does not contain a significant number of protruding residues and is thus correctly captured by **SC1** (1F6M:R, 1R8S:R, 2A9K:R, 2J7P:R from PPDBv4; 1ALL:A, 1ALL:B from Huang), (3) the protein interface displays very peculiar features and is not detected by JET^2^/iJET^2^ (1AZS:R and 1XQS:L from PPDBv4).

When JET^2^ was run on the entire PPDBv4 (except for antibodies) and Huang datasets, it automatically chose **SC2** for 126 proteins—90 from PPDBv4 and 36 from Huang. 62 of those proteins have a bound small molecule in their PDB structure (complexed conformational state). For 67% of the remaining proteins, we could find experimental evidence reported in the literature that the proteins do interact with a small molecule (see S17 Table for the list of proteins along with manually collected information about their ligands).

Overall, this analysis revealed that (*i*) exploiting the local geometry of the protein surface in combination with evolutionary information often permits to better define protein-binding patches and segregate them from small ligand-binding pockets, (*ii*) JET^2^/iJET^2^ automated clustering procedure is efficient for selecting the most appropriate scoring strategy (**SC2**).

### Predicting multi-patch interaction sites and their origins

**Fig 6:**
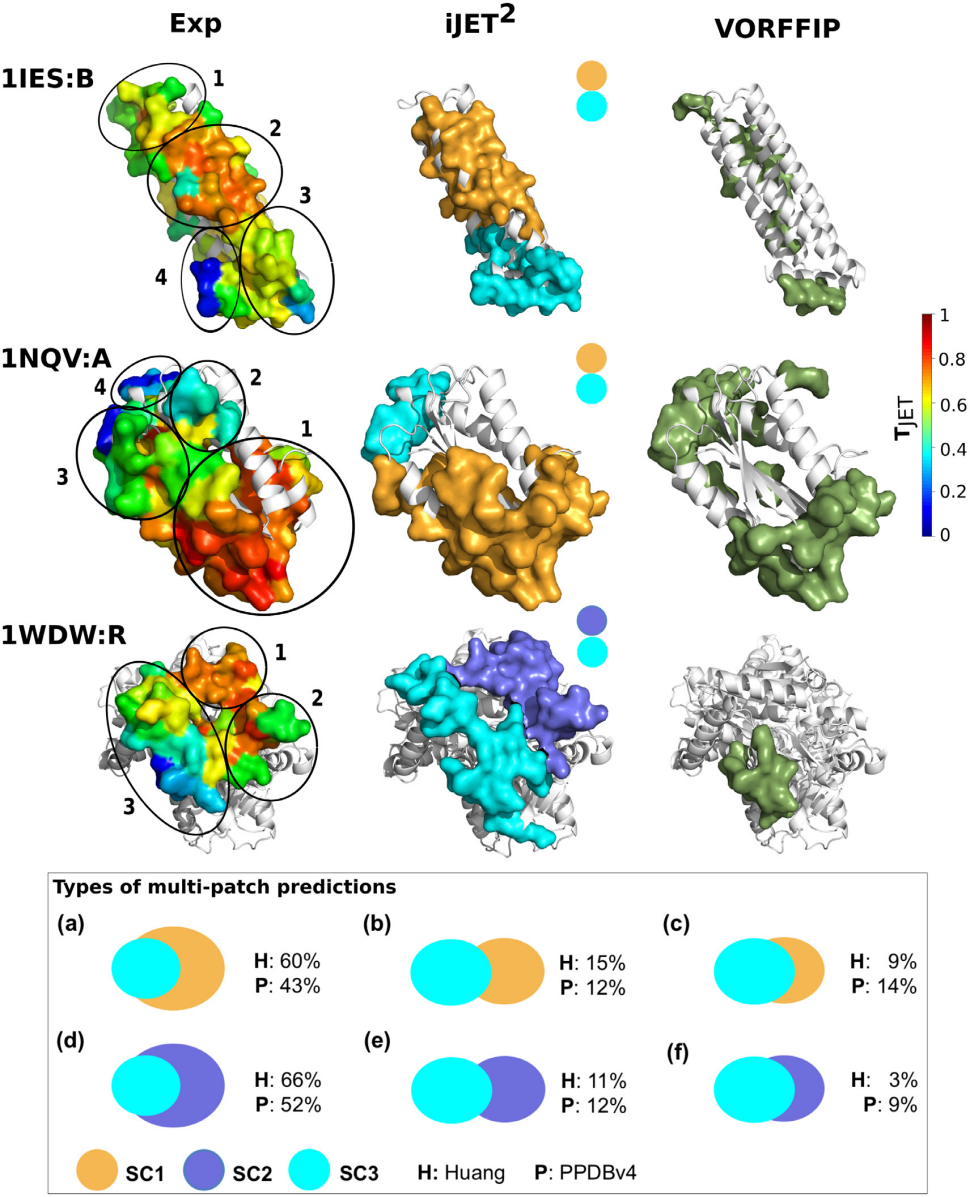
Multi-patch interaction sites of selected proteins. The experimental interface residues are colored according to T_*JET*_ computed by iJET^2^. iJET^2^ predictions were obtained from a consensus of 2 runs out of 10. They are colored according to the scoring scheme used: **SC1** in orange, **SC2** in purple and **SC3** in cyan. They were automatically chosen by iJET^2^ clustering algorithm (full procedure). The residues detected by both scoring schemes are colored in cyan (**SC3**). VORFFIP predictions are colored in dark green. The insert at the bottom shows the most frequent types of multi-patch predictions by JET^2^. Overlaps are computed as: *o*_*i*,*j*_ = #(*pred*_*i*_ ∩ *pred*_*j*_)/#(*pred*_*i*_), where *pred*_*i*_ and *pred*_*j*_ represent the residues predicted at the interface by **SC*i*** and **SC*j*** respectively. (**a,d**) *o*_1/2,3_ < 60% & *o*_3,1/2_ ≥ 60%, (**b,e**) *o*_1/2,3_ < 30% & *o*_3,1/2_ < 30%, (**c,f**) *o*_1/2,3_ ≥ 60% & *o*_3,1/2_ ≥ 60%. The proportions of predictions corresponding to each case, among all **SC1-SC3** predictions (upper row) or all **SC2-SC3** predictions (lower row), are given for Huang (H) and PPDBv4 (P).

A single (typically large) protein interface may be comprised of multiple recognition patches. Janin and co-authors [25] previously proposed to detect multiple patches within an interface by clustering the positions of the interacting atoms in 3D space [25]. They applied this clustering on experimentally known interaction sites. By contrast, JET^2^ considers the whole surface of the protein where it predicts interacting residues and groups them based on their shared evolutionary, physico-chemical and/or geometric properties and on their 3D proximity. We observed that **SC1** or **SC2** (in short **SC1-2**) and **SC3** often point to different surface regions (S2 Fig). This suggests that JET^2^ could be employed to revisit the way recognition patches are defined within protein interfaces. To test this hypothesis, we analyzed JET^2^ multi-patch predictions and we compared their **SC**-driven decomposition with the 3D position-based clustering [25] of experimental interacting residues (Fig 6).

JET^2^ complete automated clustering procedure (Fig 2D; see Materials and Methods), by combining patches detected using different scoring schemes, yielded predicted interacting surfaces of different types (insert in Fig 6). For both Huang and PPBDv4, in more than half of the cases, the **SC3**-predicted patches were smaller and partially or totally included in the **SC1-2**-predicted patches (**a, d**). We also observed cases where **SC1-2**- and **SC3**-predicted patches were either almost disjoint (**b, e**) or largely overlapping (**c, f**). For 60 proteins—44 from PPDBv4 and 16 from Huang—JET^2^ predicted a site comprised of multiple patches, *i.e.* the second round of the clustering procedure added at least 5 residues and the added residues represent more than a quarter of the whole predicted site. 57% of those predictions match experimental interfaces comprised of multiple recognition patches, according to the definition of Janin and co-authors [25].

iJET^2^ predictions for ferritin (1IES:B) and lumazine synthase (1NQV:A) contain two patches, one detected by **SC1** and the other by **SC3**, that match well one or several experimental recognition patches (Fig 6, numbered black circles). For example, in lumazine synthase (1NQV:A), the **SC1**-predicted patch matches the experimental patch numbered *1* (highly conserved) with an accuracy value of 87%. The **SC3**-predicted patch matches the experimental patches *3* and *4* (highly protruding) with an accuracy value of 88%. For tryptophan synthase beta chain 1 (1WDW:R), iJET^2^ predicted a site composed of two patches issued from **SC2** and **SC3**. The **SC2**-predicted patch corresponds to the experimental patches *1* and *2* (*Acc* = 96%) while the **SC3** prediction corresponds to patch *3* (*Acc* = 98%). Interestingly the experimental patches *1* and *2* display different characteristics: the former is highly conserved while the latter comprises a concave conserved region (in red) and lowly conserved highly protruding residues (in green). This example illustrates the power of **SC2** to detect interacting residues having different properties.

For the remaining proteins for which JET^2^ predictions are composed of multiple patches, only one predicted patch matches the experimental interface. For 2 out of 3 proteins (66%) from Huang and 11 out of 23 proteins (48%) from PPDBv4, the JET^2^ predicted patch that would be considered as false positive actually corresponds to an interaction with another partner, based on experimental data available in the literature (see S18 Table for the list of proteins along with manually collected information about their partners).

This analysis showed that JET^2^ is able to capture the spatial organization of interacting residues at the protein surface based on their evolutionary conservation, physico-chemical and geometric properties. JET^2^ provides insights on the (possibly multiple) origin(s) of an interaction, *i.e.* the set(s) of interacting residues forming the core(s) of the physical contact between the two partners. It reveals how these multiple origins shape large protein interfaces and, in doing so, it revisits the way recognition patches are defined. Moreover, it enables to characterize the different properties of these recognition patches. Such information cannot be obtained by using purely 3D position-based clustering of experimentally known interaction surfaces.

### Identifying specificity determinants based on the geometry of the predicted interface

Some proteins present several binding sites, to different partners, that have a lot of residues in common. In such cases, we expect predicted patches to encompass residues involved in the interaction(s) with one, several or all partners. Consequently, given an experimental complex, residues that would be considered as false positives might actually participate in the interaction with another partner. We used JET^2^ to understand the geometry of these patches by analyzing two proteins that interact with multiple partners via the same region of their surface.

**Fig 7:**
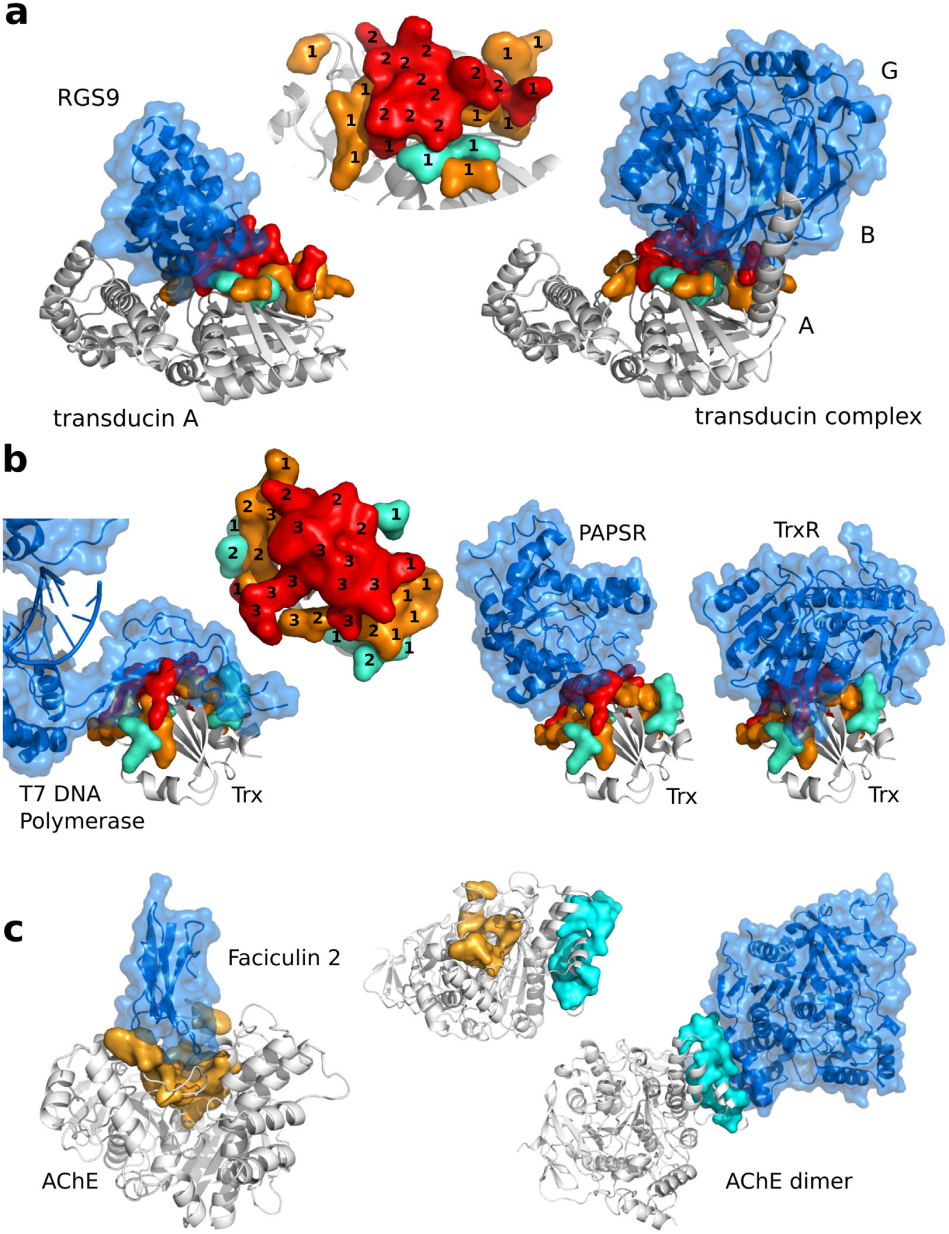
JET^2^ predictions of protein interfaces with different partners. The protein of interest is represented as a grey cartoon while its partners are displayed as blue cartoons and transparent surfaces. (**a-b**) Moonlighting proteins, interacting with different partners via the same surface region. Residues (true positives) predicted by JET^2^ (**SC2**) are displayed as a colored opaque surface: cluster seed, extension and outer layer are in red, orange and cyan. On the predicted patches are indicated, for each residue, the number of interactions it is involved in. **(a)** Complexes of transducin G_*α*_ subunit with RGS9 (on the left, PDB code: 1FQJ) and with transducin G_*βγ*_ (on the right, PDB code: 1GOT). **(b)** Complexes of thioredoxin (Trx) with T7 DNA polymerase (on the left, PDB code: 1X9M), with PAPS reductase (in the middle, PDB code: 2O8V) and with Trx reductase (on the right, PDB code: 1F6M). (**c**) Protein which partners bind to two different locations. iJET^2^ predicted patches are colored according the scoring scheme used: **SC1** in orange and **SC3** in cyan.

Transducin is a heterotrimeric guanine-nucleotide-binding (G) protein composed of three polypeptide chains *α*, *β* and *γ* (1GOT, Huang). Once activated, the G_*α*_ subunit dissociates from the complex and triggers phototransduction signaling cascade. The regulatory protein RGS9 terminates the signal by binding to G_*α*_ (1FQJ, PPDBv4). By using **SC2**, iJET^2^ predicted the G_*βγ*_- and RGS9-binding patches of G_*α*_ with high sensitivity (*Sens* = 58% and 62%) and high precision (*PPV* = 78% and 54%). The two patches predicted over the two structures share 73% of their residues (Fig 7A). Among the true positives, 64% (9/14) of the seed residues are involved in both interactions whereas most if not all residues from the extension and the outer layer are specific of the interaction with one partner (S4 Table).

Thioredoxin (Trx) from *E. coli* facilitates the reduction of other proteins such as PAPS reductase (2O8V, PPDBv4). Trx is itself reduced by Trx reductase (1F6M, PPDBv4). In addition to its anti-oxidant function, Trx also plays a structural role upon bacteriophage T7 infection by associating with T7 DNA polymerase (PDB code: 1X9M) [45]. This ability to perform different autonomous functions via the same domain is typical of moonlighting proteins [46]. 86% of Trx interacting residues in structures 1F6M:L and 2O8V:L were detected by JET^2^ (**SC2**). The two predicted patches share 84% of their residues and T7 DNA polymerase also binds to this region (Fig 7B). 60% (9/15) of the seed residues are involved in all three interactions (S5 Table). By contrast 46% of the extension and 67% of the outer layer participate in only one interaction.

This analysis of the usage of an interaction site by several partners showed that the residues located in the outer layer, and to a smaller extent the extension, of JET^2^ predicted patches tend to specifically interact with one partner. Consequently, the predictive model implemented in JET^2^, composed of a seed, an extension and an outer layer, provides insight into the determinants of molecular recognition specificity. Thanks to the experimental knowledge on the two proteins studied here, we could validate JET^2^ predictions. This further suggests that JET^2^ could be useful for identifying moonlighting proteins and help to learn about a protein’s partners by looking at the characteristics of the interaction site. For instance, given a protein for which we know a partner and the localisation of its binding site, the presence of a significant number of false positives located in the extension and/or outer layer of a patch predicted by JET^2^ may indicate that this patch is involved in the association with another partner—and that the false positive residues actually interact with this other partner. To test this hypothesis, we selected the proteins from PPDBv4 whose interaction site was predicted by **SC1** with high sensitivity (> 80%) but rather low precision (< 60%) and looked for additional structural data in the Protein Data Bank (PDB). Among the 11 identified proteins, we could identify another partner for 6 (55%) of them and match the false positives of JET^2^ prediction with the corresponding interaction site (see S19 Table for the list of proteins along with manually collected information about their partners).

### An improved overall performance

**Fig 8:**
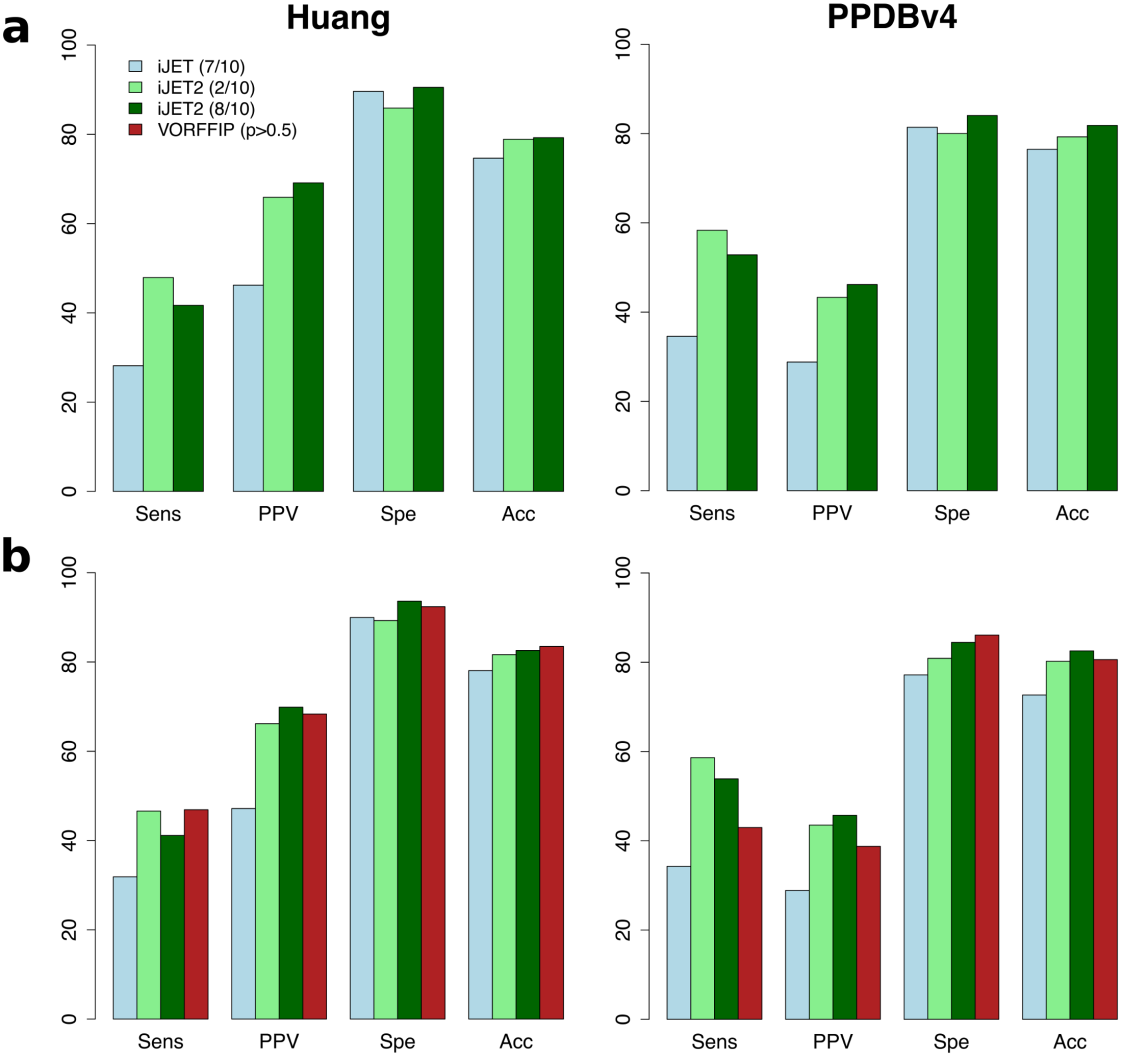
Summary of iJET^2^, JET and VORFFIP performance. Average sensitivity (*Sens*), positive predictive value (*PPV*), specificity (*Spe*) and accuracy (*Acc*) are plotted for proteins from the Huang dataset (on the left) and PPDBv4 (on the right). (**a**) all proteins considered; (**b**) only proteins that are not part of VORFFIP training set. For iJET, consensus predictions were obtained from 7 runs out of 10 (light blue). For iJET^2^, consensus predictions were obtained from 2 (in light green) and 8 (in forest green) runs out of 10. Setting the threshold at 2 yields higher sensitivity while setting it at 8 yields higher precision and specificity. The clustering procedure was run using all three scoring schemes for each protein and the best patch or combination of patches was retained for performance assessment. For VORFFIP, predicted patches were defined as formed by residues with scores greater that 50 (in firebrick).

A large scale assessment of the role played by structural information in JET^2^ was realized by comparing the results of JET^2^ to those of JET, which is based on sequence information only. Proteins may interact with different partners via several binding sites located in distinct regions of the protein surface. An example is given by acetylcholinesterase (1MAH, PPDBv4) for which **SC1** predicted a patch that corresponds to the binding site of the snake toxin fasciculin and **SC3** predicted well the protein homodimeric interface (Fig 7C). Consequently, to compute JET^2^ performance on the testing sets, we ran the tool by selecting manually each of the three scoring schemes and we considered the best patch or combination of patches. iJET^2^ consensus predictions (2 runs out of 10, best precision/recall balance) cover about 50% and 60% of all protein interfaces from Huang and PPDBv4, with precision of 66% and 43% respectively (Table 1 and Fig 8). These values are increased by 15 to 25 compared to values computed for iJET consensus predictions (7 runs out of 10). This significant improvement of JET^2^ over JET performance is consistently observed when considering consensus predictions obtained from 1 to 10 runs out of 10 and the different functional and structural classes of the two testing sets (S6 and S7 Tables and S3 Fig). The improvement is less striking when we consider the predictions obtained from JET^2^ complete automated clustering procedure (see ${\text{iJET}}_{AutoComplete}^{2}$ in Table 1). This reflects the fact that the entirely automatic process may detect patches that are not present in the testing sets, *i.e.* patches possibly involved in interactions with other partners (see Fig 7C and S18 Table for such cases). Performance values for individual proteins are reported in S8, S9 and S10 Tables for Huang and S11, S12, S13 and S14 Tables for PPDBv4.

### Comparison with other tools

The strategy employed in JET^2^ combines three physically and biologically meaningful residue-based descriptors in a rational way to predict protein-protein interfaces at large scale. To evaluate the relevance of our approach, we compared the results of iJET^2^ to those of three state-of-the-art methods that use machine learning, namely VORFFIP, PredUs and eFindSite^PPI^.

The VORFFIP method [36] integrates a broad set of residue descriptors—including solvent accessibility, energy terms, sequence conservation, crystallographic B-factors and Voronoi Diagrams-derived contact density—in a two-steps random forest ensemble classifier. We applied VORFFIP on the Huang dataset and PPDBv4, after having removed the proteins that were used for training the method (Table 1 and Fig 8). VORFFIP predictions were defined from residues having a normalized score (or probability) greater than 0.5. The overall performance of iJET^2^ (2 or 8 runs out of 10) is comparable to that of VORFFIP on the Huang dataset (S6 Table). On PPDBv4, iJET^2^ achieved higher sensitivity and higher precision than VORFFIP, with similar specificity and accuracy values (S7 Table). iJET^2^ performed particularly better on the antibody-antigen and bound antibody-antigen complexes and on the proteins with other function from PPDBv4. Fig 3 shows examples where iJET^2^ single-patch predictions give a better coverage (1TMQ:R), better overall localization (1K5D:L) or more precise definition (1E4K:L) of the experimental protein interfaces than VORFFIP predictions. iJET^2^ also detects more experimental interacting residues in the multi-patch predictions reported on Fig 6.

The PredUs method [37, 47] predicts protein interfaces by using only information from structural neighbors of the query protein for which experimental data on their interacting surface is available. The development of the method was motivated by the observation that experimentally known interfaces are conserved across proteins that adopt similar structures [37]. Given a query protein structure, PredUs maps residues of structural neighbors involved in an interaction to residues on the surface of the query. Scores are calculated for all residues based on the mapped contacting frequencies by using a support vector machine [47]. The method was ranked first in a recent comparative evaluation of different protein-protein interfaces prediction tools [48]. We used the PredUs web server [47] to predict binding sites on the proteins from Huang and PPDBv4. We excluded proteins from PPDBv4 that were used for training the method (proteins from PPDBv3 [49]) and proteins containing several chains, as PredUs can only treat single chains. The predictions were defined from residues with strictly positive raw scores (default settings). PredUs achieved an average sensitivity of 80% and precision of 53% on all but 2 proteins from Huang, for which no structural neighbors could be found (S4 Fig). Among the 92 tested proteins from PPDBv4, PredUs calculation failed because of the absence of any structural neighbor for 11 proteins (12%). On the remaining proteins, PredUs predictions cover on average 65% of the interaction sites with a precision of 34%, while iJET^2^ average sensitivity and precision values are 59% and 43%. Consequently, PredUs predicted overall more interacting residues than iJET^2^ but with lower precision. In addition, there are a significant number of cases where the PredUs calculation failed. For the majority of these cases, iJET^2^ performed very well: 10/13 predictions have sensitivity values above 50% (up to 100%) and 7/13 predictions have precision values above 50% (up to 82%). In 9 cases over 13, **SC3** alone or in combination with **SC1** or **SC2** yielded the best prediction.

The eFindSite^PPI^ method [38] combines meta-threading, structural alignment and machine learning algorithms to predict the residues involved in protein-protein interfaces and their molecular interactions—hydrogen bonds, hydrophobic contacts, salt bridges, aromatic interactions. Given a protein query structure, eFindSite^PPI^ applies meta-threading to identify structurally and functionally related templates and maps the known interfaces of the templates to the query protein using structural alignment. Then, the algorithm combines the information from the templates with four residue-based descriptors (relative accessible area, generic and position-specific interface propensities, sequence entropy) to compute interfacial probability scores for every residues of the query protein, by using support vector machines and a naive Bayes classifier. The method was shown to outperform PredUs when protein models are given as inputs instead of experimentally determined structures [48]. eFindSite^PPI^ was applied to the proteins from the Huang dataset, for which it showed better performance values than iJET [38]. On average, eFindSite^PPI^ achieved a sensitivity of 62% and a precision of 62% (see Table 5 in [38]). iJET^2^ displays a lower sensitivity of 48% (resp. 42%) but a higher precision of 66% (resp. 69%) with 2 (resp. 8) runs out of 10. We used the eFindSite^PPI^ web server [38] to predict binding sites on the three examples illustrating JET^2^ ability to predict and dissect multi-patch interaction sites (Fig 6). About one third of the interface residues of ferritin (1IES:B, *Sens* = 33%) and lumazine synthase (1NQV:A, *Sens* = 36%) were detected by eFindSite^PPI^ with very high precision of 96% and 94% respectively (S5 Fig). The predicted residues are sparsely distributed at the protein surface and do not form multiple identifiable patches, as was observed for iJET^2^ predictions (Fig 6). The case of tryptophan synthase beta chain 1 (1WDW:R) could not be treated by eFindSite^PPI^ due to a too large number of residues in the protein structure.

Overall, these analyses showed that the performance values computed for JET^2^/iJET^2^ on Huang and PPDBv4 are comparable to those computed for state-of-the-art methods that use machine learning algorithms. An advantage of our method is that it predicts interacting residues that form recognition patches, it gives insights into the origins of these patches and it enables to contrast the different properties of the interacting residues and their role for the molecular recognition specificity. It can be applied to proteins for which a structurally similar protein with experimentally characterized interface(s) cannot be found, to proteins of arbitrary length and to multi-chain complexes.

## Discussion

The geometrical support-core-rim model suggested us a novel way to predict protein binding sites. We highlighted a structural feature of these sites that was never exploited for PPI prediction before: the rim mainly consists of protruding residues. We showed that circular variance represents an elegant solution for detecting such residues, especially when the evolutionary signal is very low. Moreover, when combined with conservation levels, CV achieved extremely high precision in the detection of PPI-mediating residues versus nearby residues forming small-molecule binding pockets.

A previous analysis of solvent-exposed loops in single-domain proteins suggested that some loops may drive fulfillment/prevention of homodimerization [50]. In our predictions, almost 70% of the protruding residues (1-CV>0.5) form up to 8-residue-long loops. We extend the intuition of enabling/disabling loops to protruding highly localized regions and we propose that these regions may play the role of anchors in PPIs.

We have addressed the issue of the specific and precise automatic detection of protein interaction surfaces with JET^2^. We predicted interfaces for 238 protein complexes forming a wide spectrum of functional and structural classes. The construction of a predicted interface through the generation and the fusion of patches associated to different scoring schemes allows JET^2^ to identify different origins of the interactions and recover the dissecting patterns of protein-protein recognition sites that can be obtained by residue density analysis of experimental interfaces [16]. We explored these protein interactions at the residue level and analyzed the binding sites shared by several proteins at the same time or used, possibly partially, by proteins in different moments.

### Antibody interfaces

Antibody Fab fragments are comprised of framework regions, that are absolutely or very strongly conserved [51], and hypervariable loops that recognize the antigen. They represent a paradigm for protein recognition specificity as one antibody usually specifically targets one antigen [52]. Thornton and co-authors previously demonstrated that the “antigenic” residues of antibodies tend to be located in loops which protrude from the surface of the protein [43, 44]. In agreement with these early studies, we showed that JET^2^ predicts remarkably well the antigen-binding sites of the 25 antibodies from PPDBv4 by exploiting the local geometry of the protein surface (**SC3**). Interestingly, the evolutionary trace-driven strategy (**SC1**) captured with high accuracy the interfaces between light and heavy chains (S6 Fig). This observation suggests that JET^2^ different scoring schemes permit to detect patches involved in different types of interactions and subject to different evolutionary constraints at the surface of a protein.

### Prediction of multiple partners

JET^2^ can be used to predict and learn about the different partners of a protein in several ways. First, it can detect the presence of a putative small ligand binding site at the surface of the protein based on the specific properties of these sites compared to protein-protein interaction surfaces. When the cluster seed detected by **SC1** is highly conserved, then a small ligand binding site is suspected and the program will automatically switch to **SC2** on order to specifically detect the protein binding site. Second, a prediction, obtained by running JET^2^ complete automated procedure, comprised of multiple patches that do not or slightly overlap, may indicate that the protein can interact with two different partners binding to the two detected locations. Thirdly, a large predicted patch matching a known interaction site with high sensitivity but low precision can reveal the shared usage of the detected region of the protein surface by several partners.

### Design of inhibitors

Lymphocyte function-associated antigen-1 (LFA-1) is an integrin that plays a crucial role in antigen-specific responses [53] by recognizing intercellular adhesion molecule 1 (ICAM-1) (1MQ8, PPDBv4). By using **SC1**, JET^2^ successfully predicted ICAM-1 binding site of LFA-1 (*Sens* = 67% and *PPV* = 48%). Another patch was predicted by using **SC3** and the overlap between the two patches is 50%. We could identify five residues in the **SC3**-predicted patch that are not involved in the interaction with ICAM-1 but are found in the interface with Efalizumab (PDB code: 3EOA) (S7 Fig). Efalizumab is an antibody drug used in the treatment of psoriasis which inhibits the association between LFA-1 and ICAM-1 [54]. This example suggests that JET^2^, by exploiting surface local geometry and physico-chemical properties, could be useful for identifying putative sites at the surface of therapeutic targets. This opens new perspectives for the design of highly specific drugs

These examples illustrate the predictive potential of JET^2^ to detect multiple interactions involving different partners of a protein. By manually inspecting the predictions and looking for additional experimental data reported in the literature, we could assess JET^2^ predictive power beyond the interactions present in the studied datasets. It is likely that the increasing availability of experimental structures of protein complexes will further validate JET^2^ predictions in the future. Let us stress however that many aspects of protein regulation (*e.g.* post-translational modification, differential expression…), which are out of the scope of this work, may also influence protein binding specificity.

We have carefully compared the performance of JET^2^ with state-of-the-art methods for predicting protein-protein interfaces based on machine learning algorithms. JET^2^ is computationally much simpler than any machine learning approach. It combines in a rational way three residue-based descriptors that have straightforward biological interpretability. This strategy allows to reach comparable or even better results than dozens of features used to train unsupervised classifiers. We thereby demonstrate that machine learning is not a necessary algorithmic approach for protein-protein binding site prediction. To have few features instead of dozens is important while exploring the very large landscape of protein interaction sites, since many of these sites might not be classifiable yet, and many others might be overlapping, making their recognition a difficult task for learning algorithms. A clear advantage of JET^2^ is that it provides a detailed residue-based characterization of the conservation, physico-chemical and geometrical properties of the recognition patches. By characterizing different signals at the surface of a protein, it enlightens the complexity of this surface and more specifically of the surface regions involved in functional interactions. It can be used to discover novel interfaces, which is not possible with methods based on the learning of experimentally known interfaces of structurally similar proteins.

Our evaluation of iJET^2^ performance shows that the first step of the clustering procedure, *i.e.* seed detection, yields very precise predictions with zero or very few false positive(s) for a large number of proteins (S15 Table). The subsequent steps, extension and addition of an outer layer, essentially contribute to increasing the coverage of the interface. Consequently the tool can be used in a broad set of contexts depending on whether one is interested in achieving high coverage of experimental interfaces, or obtaining very high precision for further use of the predictions to discriminate partners within the cell [40]. Furthermore, the predicted interfaces can be used to guide mutation studies. Specifically, the core residues are particularly well predicted (best precision/recall balance) by JET^2^ (Fig 4C). The method can also be useful to probe the evolutionary landscape of protein interfaces, and to propose allosteric sites that could be targeted in the context of drug development. Last, it can be used to provide a straightforward understanding of the molecular descriptors that are characteristic of protein interfaces.

## Materials and Methods

### Levy model of experimental interfaces

We describe experimental protein interfaces by using the Support-Core-Rim model information obtained from complex structures (Fig 2A, *on the left*). The Levy model [24] argues that interface residues play different roles in the interaction depending on the geometry of the interacting surface. Interface residues are defined based on relative solvent accessible surface area (*rasa*) changes, as computed by NACCESS [55] with a probe radius of 1.4Å, between the unbound and bound protein in complexed conformational state (S3 Table). The interface is divided in three structural components: *support* residues are buried in unbound and bound protein (*rasa*_*u*_ < 0.25, *rasa*_*b*_ < 0.25); *core* residues become buried upon binding to the partner (*rasa*_*u*_ ≥ 0.25, *rasa*_*b*_ < 0.25); *rim* residues are exposed in unbound and bound protein (*rasa*_*u*_ ≥ 0.25, *rasa*_*b*_ ≥ 0.25). The support and the rim resemble protein interior and surface respectively, while the core has a specific amino-acid composition.

### Datasets of protein-protein interactions

Two datasets were used to analyze experimental protein interfaces and evaluate JET^2^ performance: the Huang dataset of 62 protein complexes [20] which was previously used to assess JET performance [39], and the Protein Protein Docking Benchmark version 4 (PPDBv4) of 176 protein complexes [41] (S1 Table). The Huang dataset comprises 41 homodimeric chains, 24 heterodimeric chains and 19 transient chains. PPDBv4 proteins can be grouped in four functional classes—antibody-antigen (26), bound antibody-antigen (24), enzyme-inhibitor (104) and proteins with other functions (198). PPDBv4 collects both their free and complexed states. Experimental interfaces were defined using the complexed conformations while JET^2^ predictions were performed on the free conformations. Given the variety of proteins and protein complexes represented and the presence of both free and complexed states, these testing data sets constitute a rich mine of information.

### JET workflow

JET^2^ development is based on the large-scale method Joint Evolutionary Trees (JET) [39]. JET predicts binding patches for protein families by combining residue conservation (T_*JET*_) and amino-acid physico-chemical properties (PC). JET does not need to know the binding partner but only requires information on a single query protein. The tool was designed to (*i*) detect very different types of interfaces (with a protein, a small molecule, DNA or RNA), (*ii*) provide predictions even with weak signal thereby ensuring broad applicability, (*iii*) provide robust consensus predictions from iterative runs (iJET). An important characteristics of JET algorithm is that it uses suitable heuristics to detect and extend binding patches. Consequently alternative predictions are produced by different JET runs. Consensus predictions are defined from the likelihood of a residue to belong to the interface predicted in several JET independent runs.

### JET^2^ workflow

The JET^2^ method requires as input a protein query sequence for which three-dimensional structural coordinates are available in the Protein Data Bank (PDB) [56]. In a first step, JET^2^ determines the conservation level (T_*JET*_) of every amino acid in the query sequence. The calculation is performed on a set of homologous sequences (ideally 100 or more). This step, essentially unchanged compared to JET, was extensively described in [39].

In a second step, JET^2^ detects and extends putative binding patches at the surface of the three-dimensional structure of the query protein. For this, residues are scored using a mixture of three descriptors: the evolutionary traces (T_*JET*_) computed in step 1, interface propensities (PC) specific to each amino acid and circular variances (CV) computed from the protein 3D structure and representing the density of protein around each residue. JET^2^ implements three different scoring schemes, *i.e.* combinations of the three descriptors (Fig 2C and S3 Table). They can be used alternatively depending on the system studied, according to the user’s choice or through an automated procedure (Fig 2D and Table 2). JET^2^ clustering algorithm unfolds as follows: (*1*) highly-scored residues are selected and grouped together based on 3D proximity (< 5Å) to form cluster seeds; (*2*) seeds are extended by progressively adding highly-scored neighboring residues until a cluster mean score threshold is reached down; (*3*) an outer layer is added comprised of highly protruding residues. Seeds too small to be considered as putative protein binding sites are filtered out, as described in [39]. The strategy employed for seed extension and the addition of the outer layer are new compared to JET and are described in details below. The obtained residue clusters represent predicted binding patches. Patches that are in close proximity at the surface of the protein can form a single interaction site. JET^2^ allows to automatically combine different binding patches predicted using complementary scoring schemes in order to form multi-patch binding sites (Fig 2D and Table 2).

**Table 2:** JET^2^ automated clustering algorithm.

	**First round to detect main clusters**
**if** #(*T*_*JET*_ > 0.6)/*N* < 0.3 * *f*_*intfrac*_(*N*)—————–	• Test whether the evolutionary signal is too low: *N*, number of surface residues *f*_*intfrac*_(*N*), expected size of the interface #(*T*_*JET*_ > 0.6)/*N*, proportion of highly conserved residues
**then**	
choose **SC3**;	
**else**	
choose **SC1**;	
**end**	
detect cluster seeds;	
**if SC1 then**	
**if** *Seeds* = ∅————————————————	• Test whether **SC1** found some seed: *Seeds*, set of seeds
**then**	
choose **SC3**;	
detect cluster seeds;	
**else**
**if** $\frac{\sigma (s)}{\mu (s)}<\frac{1}{6}\frac{\sigma_{surf}}{\mu_{surf}}\text{,}$ *for all s* ∈ Seeds—————–	• Test whether the seed conservation signal is homogeneous: *σ*(*s*) and *μ*(*s*), dispersion and mean of the distribution of the scores computed over the residues in seed *s* *σ*_surf_ and *μ*_surf_, dispersion and mean of the distribution of the scores computed over all surface residues
**then**	
choose **SC2**;	
detect cluster seeds;	
**end**	
**end**
**end**	
add the extensions to the seeds;	
add the outer layers to the clusters;	
**if SC1** *or* **SC2 then**	**Second round to detect additional clusters using a complementary SC**
choose **SC3**;	
**else**	
choose **SC2**;	
**end**	
detect cluster seeds;	
add the extension to the seeds;	
add the outer layers to the clusters;	
combine clusters;	Merge main clusters with additional clusters <5Å away
*scoreMax* ← max_*r* ∈ *C*_(*score*(*r*));	**Extension of a cluster *C***
**while** $\mu \left(C\right)>score_{clus}^{CUT}$ **do**	
*newRes* ← {};	
**for** *r* ∈ {*neighbors of C*} **do**	*score*(*r*), score of the residue *r* *μ*(*C*), mean score computed over cluster *C* $score_{res}^{CUT}$, threshold score for residues $score_{clus}^{CUT}$, threshold score for clusters {*neighbors of* *C*}, ensemble of residues <5Å away from *C*
**if** $score_{res}^{CUT}<score\left(r\right)<scoreMax$ **then**	
add *r* to *newRes*;	
**end**	
**end**	
add *newRes* to *C*;	
*scoreMax* ← max_*r* ∈ *newRes*_(*score*(*r*));	
**end**
*newRes* ← {};	**Addition of an outer layer to a cluster *C***
**for** *r* ∈ {*neighbors of C*} **do**	
**if** $\frac{\mu (C)\times |C|+score(r)}{|C|+1}\geq \mu \left(C\right)$ **then**	{*neighbors of* *C*}, ensemble of residues <5Å away from *C*
add *r* to *newRes*;	
**end**	
**end**	
add *newRes* to *C*;	

In JET^2^, the clustering procedure can be performed in two modes: (1) either the user let JET^2^ automatically decide which scoring scheme is appropriate for the studied system, or (2) the user can manually choose a particular scoring scheme. The user can also decide to run only one round of JET^2^ clustering procedure (patches will be detected by the automatically or manually chosen main scoring scheme) or to run the complete procedure where a second round is performed to detect additional patches by using a scoring scheme complementary to the main one (see below). Finally, as described above for JET, JET^2^ can be run multiple times, in an iterative mode of the program (iJET^2^), to get more robust predictions.

### Sequence, physico-chemical and structural descriptors

Three measures, T_*JET*_, PC and CV, were introduced in JET^2^ to evaluate single residues in a protein. Conservation levels (T_*JET*_) are computed from phylogenetic trees constructed using sequences homologous to the query sequence and sampled by a Gibbs-like approach [39]. *N* trees are constructed from *N* representative subsets of sequences. For each position in the query sequence, a *tree trace* is computed from each tree *T*: it corresponds to the level *n* in the tree *T* where the amino acid at this position appeared and remained conserved thereafter [39]. Let us recall that JET definition of evolutionary trace is notably different from the measure defined by Lichtarge and co-authors to rank protein residues [57, 58].

*Tree traces* are averaged over the *N* trees to get more statistically significant values, which we denote *relative trace significances*. The final T_*JET*_ value of amino acid *a*_*j*_ at position *j* is obtained by accounting for *a*_*j*_’s environment and is expressed as follows [39]:

$$\begin{array}{c} T_{JET}\left(j\right)=\frac{w_{I}*\left(\frac{1}{\left|I\right|}\sum_{h\in I}d_{h}\right)+w_{j}*d_{j}}{w_{I}+w_{j}} \end{array}$$ (1)

where *I* is the set of residue positions which are neighbors of *a*_*j*_ (*i.e.* with at least one atom distant by less than 5Å to at least one atom of *a*_*j*_) and where *d*_*j*_ is the *relative trace significance* of *a*_*j*_. The weights were fixed at *w*_*I*_ = 3 and *w*_*j*_ = 4, as in [39]. T_*JET*_ values are scaled between 0 (least conserved residue of the protein) and 1 (most conserved residue of the protein) for the calculation of residue scores.

Physico-chemical properties (PC) are derived from propensities specific to every amino acid to be located at a protein interface, taken from [59]. The original values, ranging from 0 to 2.21, are scaled between 0 and 1 for the calculation of residue scores.

Circular variance (CV) is a measure of the vectorial distribution of a set of neighboring points around a fixed point in 3D space [60]. For a given residue, CV reflects the density of protein around it. CV has the advantage of changing more smoothly than surface accessibility in passing from the surface to the interior of the protein [61], making it less sensitive to small conformational changes. CV can be applied equally well to atomic or coarse-grain representations [60]. The CV value of an atom *i* is computed as:

$$\begin{array}{c} CV\left(i\right)=1-\frac{1}{n_{i}}\left|\sum_{j\neq i,r_{i}\leq r_{c}}\frac{\vec{r_{ij}}}{∥\vec{r_{ij}}∥}\right| \end{array}$$ (2)

where *n*_*i*_ is the number of atoms distant by less than *r*_*c*_Å from atom *i*. The CV value of a residue *j* is then computed as the average of the atomic CVs, over all the atoms of *j*. A low CV value indicates for a residue that it is located in a protruding region of the protein surface. CV values are scaled between 0 (most protruding residue of the protein) and 1 (least protruding residue of the protein) for the calculation of residue scores.

The cutoff distance *r*_*c*_ directly influences the resolution of the protein surface. Here we chose to use two different values of *r*_*c*_. We set *r*_*c*_ = 100Å when using **SC3** as the main scoring scheme, to be able to capture the most protruding regions of the protein surface at a global level. Otherwise we set *r*_*c*_ = 12Å to obtain a local description of the geometry of the protein surface. Values in the range 10–14Å for *r*_*c*_ give similar results.

CV complement (1-CV) was preferred to CV to visualize the results because it permits to better contrast this measure with the two other descriptors,T_*JET*_ and PC.

The descriptors T_*JET*_, PC and CV are combined in a very straightforward way in the different scoring schemes, without any specific weight. Each residue score is simply computed as the arithmetic mean of the scaled T_*JET*_, PC and/or (1-CV) values (Fig 2C and S3 Table). Let us stress that the scaling of T_*JET*_, PC and (1-CV) values between 0 and 1 is performed by considering the variability of the values for residues in the query protein. As a consequence, interface predictions are protein-specific.

### Expected size of the interface

The expected size of a protein interface is computed as *f*_*intfrac*_(*N*) = (26.54/*N*) + 0.03, where *N* is the number of surface residues [39]. The parameters in this analytical expression were determined in [39] based on a dataset of 1256 protein chains collected from the PDB [62]. The function also approximates well the experimental data from PPDBv4 (S8 Fig). *f*_*intfrac*_(*N*) is used in JET^2^ clustering procedure to define two thresholds (Table 2). The $score_{res}^{CUT}$ threshold is the score determined with a confidence level of 2 × *f*_*intfrac*_(*N*) on the distribution of score values. It is used to select appropriate residues for constructing the clusters. The $score_{clus}^{CUT}$ threshold is the score determined with a confidence level of *f*_*intfrac*_(*N*)/*x*, where *x* values 2 or 4, on the same distribution. It is used to decide when to terminate the cluster seed construction step (*x* = 4) and the cluster extension step (*x* = 2). These thresholds are the same as those used in JET and more details on how they are determined and employed can be found in [39].

### JET^2^ extension algorithm

JET^2^ algorithm for extending cluster seeds reflects the decreasing gradient of conservation and interface propensities observed in the experimental interfaces from support through core to rim (Fig 1A and Table 2). Specifically, at each iteration *n* of the JET^2^ extension step, we list all neighboring residues, *i.e.* residues that are distant by less than 5Å from any residue of the considered cluster. Among this ensemble of neighbors, residues are included in the cluster extension only if they display scores lower than the maximum score computed among the residues that were added in the previous iteration *n* − 1 (*scoreMax*, in Table 2). This constraint imposed on individual residues was not used in the extension algorithm of JET [39]. The algorithm stops when the average score of the cluster reaches down the threshold $score_{clus}^{CUT}$, determined with a confidence level of *f*_*intfrac*_(*N*)/2 on the distribution of residue scores [39]. Following the extension step, clusters that neighbor each other (distant by < 5Å) are merged.

### JET^2^ outer layer detection algorithm

The final step of JET^2^ clustering procedure runs in only one iteration and the strategy adopted is quite different from that of the regular extension step (described above). Each cluster is extended toward neighboring residues (distant by < 5Å) that have a high score—combining PC and CV, provided that the inclusion of the considered residue leads to a mean cluster score as high as or greater than before the inclusion (Table 2). The purpose of this additional step is to include highly exposed protein regions—protruding loops typically—that often compose the rim of experimental interfaces. Clusters are not merged anymore at this stage. Notice that this step is completely new compared to JET clustering procedure [39] and it handles clusters whose residues are detected using CV, a measure that was not considered in JET.

### Automated JET^2^ procedure

The user can let JET^2^ automatically choose the scoring scheme appropriate to the studied system (Fig 2D). The implemented algorithm proceeds as described in Table 2. First, the proportion of highly conserved residues (T_*JET*_ > 0.6) is compared to the expected size of the interface, *f*_*intfrac*_(*N*). If the protein surface is characterized by evolutionary signals too low to help detect protein interfaces, then the strategy is to look for sites comprised of protruding residues that satisfy expected physico-chemical properties (**SC3**). Otherwise, **SC1** is used to detect conserved seeds. If the search finds no seed with satisfiable size, then the strategy is switched for **SC3**. Otherwise, if a seed displays a very homogeneous conservation signal, *i.e. the ratio of the dispersion to the mean of the conservation score computed over the seed is much smaller (<1/6) than the ratio computed over the whole surface*, then a protein-small ligand binding site is suspected and the strategy is to use **SC2**. Otherwise, the seeds are considered to display good characteristics and **SC1** is further employed for their extension and the addition of the outer layer.

### Manual JET^2^ procedure

Alternatively the user can select the scoring scheme of his/her choice. He/She can decide to run only the seed detection step, the seed detection and extension steps, or all three steps of JET^2^ clustering procedure.

### Complete JET^2^ procedure

Whether JET^2^ procedure is run in the automated mode or the manual mode, the user can decide to run only one round of the clustering algorithm (main clusters will be detected by the automatically or manually chosen scoring scheme) or the complete procedure where a second round of the clustering is performed to detect complementary clusters by using a scoring scheme different from the main one (Table 2). **SC3** will be used in the second round if **SC1** or **SC2** was chosen as the main scoring scheme, and **SC2** will be coupled with **SC3** when **SC3** is chosen as the main scoring scheme (Fig 2D). If a new site is located sufficiently close (less than 5Å) to the first predicted site, then it will be fused together with the first site.

### Iterative mode

For a given query protein and a fixed set of homologous protein sequences, different independent runs of JET^2^ may produce slightly different results (typically differing by a few amino-acid residues). This is due to the heuristics employed to compute evolutionary traces T_*JET*_ and to filter out small cluster seeds (see [39]). To get more robust predictions, JET^2^ can be run multiple times, in an iterative mode of the program which we call iJET^2^. For each residue, we count the number of runs where it was detected in a cluster and divide it by the total number of runs. The resulting number is comprised between 0 and 1 and corresponds to the probability of each residue to be predicted at an interface. In order to define interaction patches, we set up a threshold from which we get a consensus prediction. Varying the threshold permits to shift the balance between sensitivity and precision. The optimal number of independent runs for establishing the consensus was evaluated to be 7 out of 10 for JET [39]. In JET^2^, the predictions are stable from 2 runs out of 10.

### Evaluation of JET^2^

We previously showed that consensus predictions obtained by running multiple independent runs of JET were more accurate than predictions from a single run [39]. Consequently we used the iterative mode of JET^2^ (iJET^2^) to evaluate the performance of the tool. We relied on the following quantities: the number of residues correctly predicted as interacting (true positives, TP), the number of residues correctly predicted as non-interacting (true negatives, TN), the number of non-interacting residues incorrectly predicted as interacting (false positives, FP) and the number of interacting residues incorrectly predicted as non-interacting (false negatives, FN). We used the four standard measures of performance: sensitivity *Sens* = *TP*/(*TP* + *FN*), specificity *Spe* = *TN*/(*TN* + *FP*), accuracy *Acc* = (*TP* + *TN*)/(*TP* + *FN* + *TN* + *FP*) and positive predictive value *PPV* = *TP*/(*TP* + *FP*). We also evaluated the statistical significance of *Sens*, *Spe*, *PPV* and *Acc* by using scores that account for the expected values of these measures. Expected numbers of true/false positives/negatives are computed as: *TP* ^*exp*^ = *C* ⋅ *S*, *TN* ^*exp*^ = (1 − *C*)(*N* − *S*), *FP* ^*exp*^ = *C* ⋅ (*N* − *S*), *FN* ^*exp*^ = (1 − *C*) ⋅ *S*, where *C* = *P*/*N* in the coverage obtained with JET^2^, *P* is the number of surface residues predicted by JET^2^, *N* is the total number of surface residues and *S* is the number of residues in the experimental interaction site. Note that the calculation of expected values assumes that *C* ⋅ *N* residues have been selected at random as being positives on the structure of the protein under study. This means that expected values depend on the protein studied. The expected sensitivity, specificity, positive predictive value and accuracy are then computed as: *Sens* ^*exp*^ = *C*, *Spe* ^*exp*^ = 1 − *C*, *PPV* ^*exp*^ = *S*/*N*, *Acc* ^*exp*^ = ((1 − *C*) ⋅ (1 − *S*/*N*)) + *C* ⋅ *S*/*N*. Pertinence scores are expressed as: *ScSens* = *Sens* − *Sens* ^*exp*^, *ScSpe* = *Spe* − *Spe* ^*exp*^, *ScPPV* = *PPV* − *PPV* ^*exp*^ and *ScAcc* = *Acc* − *Acc* ^*exp*^. The R software [63] was used to compute all performance values and produce the corresponding graphs.

### Geometry-based clustering of interface residues

Multiple recognition patches within protein experimental interfaces from the Huang dataset and PPDBv4 were detected by applying hierarchical clustering to interface residues using the average linkage method, as shown in [25]. Calculations were performed with the R software [63]. A threshold distance of 20Å enabled to obtain values similar to those reported in [25]. However, contrary to [25], for each complex we consider the two interfaces from the receptor and the ligand individually. The two datasets show very different trends: while the majority of the proteins from all functional classes of PPDBv4 contain one single recognition patch, more than 70% of the homodimers, heterodimers and transients from the Huang dataset have multi-patch interaction sites, containing up to 7 patches (S16 Table).

### Comparison with other tools

The Multi-VORFFIP webserver [64] was used to predict binding sites on the proteins from PPDBv4 and Huang. The proteins listed in the O333 dataset [65], which was used to train the method, were excluded. Given a query protein, Multi-VORFFIP gives four predictions corresponding to putative interfaces with a protein, DNA, RNA or a peptide. We considered only predictions related to protein-protein binding sites. The prediction consists in two scores assigned to each residue of the query protein: a raw score and a normalized score (or probability). Following the recommandations of the authors of the method, we used the normalized score and set up a threshold of 0.5, which seemed to yield the best balance between sensitivity and precision. The PredUs web server [47] was also used to predict binding sites on the proteins from Huang and PPDBv4. Proteins from PPDBv4 that were used for training the method (proteins from PPDBv3 [49]) and proteins containing several chains were excluded. The prediction consists in an interfacial score attributed to each residue reflecting the distance above (positive score) or below (negative score) the hyperplane defined by the Support Vector Machine classifier of the method. Residues displaying positive scores were considered as predicted to be at the interface (default settings). Finally, the eFindSite^PPI^ webserver [38] was used to predict the protein binding sites of selected proteins. The prediction consists in a list of confidently predicted interfacial residues determined from several interfacial probability scores obtained by using five features integrated in two different classifiers.

## Supporting Information

S1 Table Numbers of proteins contained in each class of the Huang dataset and PPDBv4. (PDF)

S2 Table List of proteins from PPDBv4 with a bound small molecule. (PDF)

S3 Table Specifications of the experimental and predictive models for protein interfaces. (PDF)

S4 Table Specificity of the molecular recognition between transducin and its partners. (PDF)

S5 Table Specificity of the molecular recognition between thioredoxin and its partners. (PDF)

S6 Table Summary of the performance computed for iJET, iJET^2^ and VORFFIP on the proteins from the Huang dataset. (PDF)

S7 Table Summary of the performance computed for iJET, iJET^2^ and VORFFIP on the proteins from the PPDBv4. (PDF)

S8 Table Comparison of iJET and iJET^2^ performance on Huang dataset homodimers. (PDF)

S9 Table Comparison of iJET and iJET^2^ performance on Huang dataset heterodimers. (PDF)

S10 Table Comparison of iJET and iJET^2^ performance on Huang dataset transients. (PDF)

S11 Table Comparison of iJET and iJET^2^ performance on PPDBv4 antibodies-antigens. (PDF)

S12 Table Comparison of iJET and iJET^2^ performance on PPDBv4 bound antibodies-antigens. (PDF)

S13 Table Comparison of iJET and iJET^2^ performance on PPDBv4 enzymes-inhibitors. (PDF)

S14 Table Comparison of iJET and iJET^2^ performance on PPDBv4 others. (PDF)

S15 Table Precision achieved by JET^2^ clustering steps. (PDF)

S16 Table Recognition patches defined from experimental interfaces using geometrical hierarchical clustering. (PDF)

S17 Table List of proteins from Huang and PPDBv4 for which SC2 was automatically chosen by JET^2^ and that do not contain a bound small molecule. (PDF)

S18 Table List of proteins from Huang and PPDBv4 for which JET^2^ yielded multi-patch predictions, obtained by combining different scoring schemes, that do not match experimental interfaces comprised of multiple recognition patches as defined by Janin and co-authors [25]. (PDF)

S19 Table List of proteins from PPDBv4 whose interaction site was predicted by SC1 with high sensitivity (> 80%) but rather low precision (< 60%). (PDF)

S1 Fig Schematic representation of JET, JET^2^ and SCR models of protein binding sites. JET predictive model for protein binding sites comprises a cluster seed, detected based on conservation levels or physico-chemical properties, and an extension, detected by using a mixture of conservation and physico-chemical properties. JET^2^ predictive model comprises a seed, and extension and an outer layer. In scoring scheme **SC1** taken here as an example, the seed is detected based on conservation levels, the extension is defined from conservation and physico-chemical properties, and the determination of the outer layer accounts for physico-chemical properties and surface geometry. The SCR model [24] of experimental protein interfaces comprises support residues, that are buried, core residues, that become buried upon the formation of the complex, and rim residues, that are exposed.
(TIFF)

S2 Fig Types of overlap between iJET^2^ predictions issued from different scoring schemes. The predictions were obtained from a consensus of 2 runs out of 10 of iJET^2^. Overlaps are computed as: *o*_*i*,*j*_ = #(*pred*_*i*_ ∩ *pred*_*j*_)/#(*pred*_*i*_), where *pred*_*i*_ and *pred*_*j*_ represent the residues predicted at the interface by **SC*i*** and **SC*j*** respectively. (**a**) *o*_1,2_ > 50% & *o*_2,1_ > 50%, (**b**) *o*_1,3_ < 10% & *o*_3,1_ < 10%, (**c**) *o*_2,3_ < 10% & *o*_3,2_ < 10%, (**d**) *o*_1,2_ < 10% & *o*_2,1_ < 10%, (**e**) 10%≤*o*_1,3_ < 60% & 10%≤*o*_3,1_ < 60%, (**f**) 10%≤*o*_2,3_ < 60% & 10%≤*o*_3,2_ < 60%, (**g**) *o*_3,1_ > 90% & *o*_1,3_ < 80%, (**h**) *o*_3,2_ > 90% & *o*_2,3_ < 80%. The proportions of proteins from the Huang dataset (H) and PPDBv4 (P) corresponding to each case are given.
(TIFF)

S3 Fig Comparison of the performance of iJET and iJET^2^. Average sensitivity and precision were computed on (**A**) the homodimers (in green), heterodimers (in blue), transients (in orange) and all proteins (in black) from Huang; (**B**) the antibodies-antigens (in orange), bound antibodies-antigens (in pink), enzymes-inhibitors (in green), others (in blue) and all proteins (in black) from PPDBv4. Predictions were obtained by a consensus over 10 runs (x-axis).
(TIFF)

S4 Fig Summary of iJET^2^ and PredUs performance. Average sensitivity (*Sens*), positive predictive value (*PPV*), specificity (*Spe*) and accuracy (*Acc*) are plotted for proteins from the Huang dataset (on the left) and PPDBv4 (on the right). Huang: 2 proteins for which PredUs calculation failed were excluded; PPDBv4: the proteins used for training PredUs, the multi-chain proteins and the 11 proteins for which PredUs calculation failed were excluded, resulting in a subset of 80 proteins. The numbers of proteins that could not be treated are indicated on the right (“No Pred”). For iJET^2^, consensus predictions were obtained from 2 (in light green) and 8 (in forest green) runs out of 10. The clustering procedure was run using all three scoring schemes for each protein and the best patch or combination of patches was retained for performance assessment. For PredUs, predicted patches were defined as formed by residues with positive scores (in firebrick).
(TIFF)

S5 Fig Examples of eFindSite^PPI^ predictions for multi-patch binding sites. The predicted residues are displayed in dark pink surface. The predictions were defined from the list of confidently predicted interfacial residues determined by eFindSite^PPI^ web server (compare to Fig 6).
(TIFF)

S6 Fig Complex between lysozyme and its antibody. The antibody’s heavy and light chains are represented as marine and pale cyan cartoons while lysozyme is in pink. The patches predicted by JET^2^ for the antibody are depicted as opaque surfaces and colored according to the scoring scheme used: **SC1** in orange, **SC3** in green.
(TIFF)

S7 Fig Complex between lymphocyte function-associated antigen-1 (LFA-1) and the antibody drug Efalizumab. LFA-1 is displayed as a grey cartoon. The heavy and light chains of Efalizumab are colored in marine and palecyan and displayed as cartoon and transparent surface. The experimental binding site of LFA-1 natural ligand is depicted as opaque grey surface. The five residues detected by iJET^2^ **SC3** that are specific to the interaction with Efalizumab are depicted as green opaque surface.
(TIFF)

S8 Fig Protein surface size and interface fraction. The fraction of the surface covered by the interface (y-axis) is plotted versus the number of surface residues (x-axis) for all 352 proteins from PPDBv4. The red line corresponds to the *f*_*intfrac*_(*N*) function.
(TIFF)
