# Supplementary material for: Local Geometry and Evolutionary Conservation of Protein Surfaces Reveal the Multiple Recognition Patches in Protein-Protein Interactions
Source: PLoS Comput Biol. 2015 Dec 21;11(12):e1004580. doi: 10.1371/journal.pcbi.1004580 (PMC4686965; doi:10.1371/journal.pcbi.1004580)
Supplement: S1 Table — (PDF) [file pcbi.1004580.s001.pdf]

	Huang				PPDBv4				
	**Homo**	**Hetero**	**Trans**	**All**	**A**	**AB**	**EI**	**O**	**All**
	41	24	19	84	24	26	104	198	372
*	35	8	10	53	14	14	52	82	162

The starred row corresponds to subsets obtained by removing the VORFFIP training set proteins.
