# Supplementary material for: Local Geometry and Evolutionary Conservation of Protein Surfaces Reveal the Multiple Recognition Patches in Protein-Protein Interactions
Source: PLoS Comput Biol. 2015 Dec 21;11(12):e1004580. doi: 10.1371/journal.pcbi.1004580 (PMC4686965; doi:10.1371/journal.pcbi.1004580)
Supplement: S2 Table — (PDF) [file pcbi.1004580.s002.pdf]

Complex PDB id	L	R
1A2K	✓	
1ATN		✓
1AZS	✓	✓
1E4K		✓
1E6E		✓
1E96		✓
1EWY		✓
1F6M		✓
1FQ1	✓	✓
1GP2		✓
1GRN		✓
1HE8	✓	
1I4D	✓	
1IB1	✓	
1IBR		✓
1J2J		✓
1JWH	✓	
1K5D		✓
1KXP		✓

Complex PDB id	L	R
1LFD		✓
1R8S		✓
1RLB	✓	
1WEJ	✓	
1WQ1	✓	
1XQS	✓	
1Y64	✓	
1Z0K		✓
1ZM4	✓	
2A9K	✓	✓
2BTF		✓
2CFH		✓
2FJU	✓	
2G77	✓	
2H7V	✓	
2J7P	✓	✓
2OOR	✓	
2PCC	✓	✓
3CPH	✓	

The PDB id of the complex and the role of the protein in the complex, either receptor (R) or ligand (L), are given.
