# Supplementary material for: Local Geometry and Evolutionary Conservation of Protein Surfaces Reveal the Multiple Recognition Patches in Protein-Protein Interactions
Source: PLoS Comput Biol. 2015 Dec 21;11(12):e1004580. doi: 10.1371/journal.pcbi.1004580 (PMC4686965; doi:10.1371/journal.pcbi.1004580)
Supplement: S3 Table — (PDF) [file pcbi.1004580.s003.pdf]

Experimental SCR Model			Predictive JET^2 Model				
**Region**	**Composition**	**Definition**	**Region**	**Selection**	**Scoring schemes**		
					**SC1**	**SC2**	**SC3**
**Support**	interior-like	$rasa_u < 25\%$	**Seed**	high-score residues	T_{JET}	$\frac{T_{JET}+(1-CV)}{2}$	$\frac{PC+(1-CV)}{2}$
**Core**	specific	$rasa_u > 25\%$ & $rasa_b < 25\%$	**Extension**	high-score neighbors	$\frac{T_{JET}+PC}{2}$	$\frac{T_{JET}+(1-CV)}{2}$	$\frac{PC+(1-CV)}{2}$
**Rim**	surface-like	$rasa_b > 25\%$	**Outer layer**	high-score neighbors	$\frac{PC+(1-CV)}{2}$	$\frac{PC+(1-CV)}{2}$	$\frac{PC+(1-CV)}{2}$
**Ensemble**	interface residues ($\Delta rasa > 0$)		**Ensemble**	surface residues ($rasa_u > 5\%$)			

For the experimental model, the amino-acid compositions and the definitions of the support, core and rim are given. $rasa_u$: relative solvent accessible surface area in the unbound protein (complexed state). $rasa_b$: relative solvent accessible surface area in the bound protein (complexed state). For the predictive JET^2 model, the strategies employed to select residues and the scoring schemes used are given. T$_{JET}$: evolutionary trace; PC: physico-chemical properties; CV: circular variance.
