# Supplementary material for: Local Geometry and Evolutionary Conservation of Protein Surfaces Reveal the Multiple Recognition Patches in Protein-Protein Interactions
Source: PLoS Comput Biol. 2015 Dec 21;11(12):e1004580. doi: 10.1371/journal.pcbi.1004580 (PMC4686965; doi:10.1371/journal.pcbi.1004580)
Supplement: S4 Table — (PDF) [file pcbi.1004580.s004.pdf]

Cluster seed

	T177	T178	G179	I180	G199	R201	S202	E203	K205	K206	W207	H209	F211	E212
1FQJ	✓	✓	✓	✓		✓	✓	✓	✓	✓		✓		
1GOT		✓	✓	✓	✓	✓	✓	✓	✓	✓	✓	✓	✓	✓
Total #	**1**	2	2	2	**1**	2	2	2	2	2	**1**	2	**1**	**1**

Cluster extension

	V175	K176	E182	V197	Q200	C210	V214	D234	R253	Y254
1FQJ	✓	✓		✓				✓		
1GOT			✓	✓	✓	✓	✓		✓	✓
Total #	**1**	**1**	**1**	2	**1**	**1**	**1**	**1**	**1**	**1**

Cluster outer layer

	I181	F195
1FQJ	✓	
1GOT		✓
Total #	**1**	**1**

True positives belonging to the cluster seed, extension and outer layer of iJET^2 **SC2**-predicted patch are listed. Residues that participate in the experimental interfaces formed by transducin in complex structures 1FQJ and 1GOT are checked.
