# Supplementary material for: Local Geometry and Evolutionary Conservation of Protein Surfaces Reveal the Multiple Recognition Patches in Protein-Protein Interactions
Source: PLoS Comput Biol. 2015 Dec 21;11(12):e1004580. doi: 10.1371/journal.pcbi.1004580 (PMC4686965; doi:10.1371/journal.pcbi.1004580)
Supplement: S5 Table — (PDF) [file pcbi.1004580.s005.pdf]

Cluster seed

	E30	W31	C32	G33	P34	E36	M37	P40	N59	D61	G71	R73	G74	I75	A93
1F6M		✓	✓	✓	✓		✓	✓			✓	✓	✓	✓	✓
2O8V	✓	✓	✓	✓	✓	✓	✓		✓	✓		✓	✓	✓	✓
1X9M	✓	✓	✓	✓	✓	✓	✓			✓		✓	✓	✓	✓
Total #	**2**	3	3	3	3	**2**	3	**1**	**1**	**2**	**1**	3	3	3	3

Cluster extension

	I41	E44	I60	Q62	P64	A67	Y70	I72	V91	G92	L94	S95	K96
1F6M	✓	✓	✓		✓	✓	✓	✓	✓		✓	✓	✓
2O8V			✓	✓				✓	✓	✓			
1X9M			✓		✓	✓		✓	✓	✓	✓		
Total #	**1**	**1**	3	**1**	**2**	**2**	**1**	3	3	**2**	**2**	**1**	**1**

Cluster outer layer

	S1	P68	K69	G97	Q98	F102
1F6M		✓	✓	✓	✓	
2O8V	✓					
1X9M		✓			✓	✓
Total #	**1**	**2**	**1**	**1**	**2**	**1**

True positives belonging to the cluster seed, extension and outer layer of iJET^2 **SC2**-predicted patch are listed. Residues that participate in the experimental interfaces formed by thioredoxin in complex structures 1F6M, 2O8V and 1X9M are checked.
