# Supplementary material for: Local Geometry and Evolutionary Conservation of Protein Surfaces Reveal the Multiple Recognition Patches in Protein-Protein Interactions
Source: PLoS Comput Biol. 2015 Dec 21;11(12):e1004580. doi: 10.1371/journal.pcbi.1004580 (PMC4686965; doi:10.1371/journal.pcbi.1004580)
Supplement: S6 Table — (PDF) [file pcbi.1004580.s006.pdf]

	Sen	ScSen	PPV	ScPPV	Spe	ScSpe	Acc	ScAcc
**Homodimers**								
iJET (7/10)	28.58	15.28	44.74	1.09	90.85	4.15	77.27	21.14
iJET^2 (2/10)	**42.66**	**29.04**	69.55	1.91	93.87	**7.5**	**82.94**	**25.17**
iJET^2 (8/10)	38.52	26.9	**71.85**	**1.97**	**95.16**	6.78	82.87	25.09
**Homodimers***								
iJET (7/10)	28.11	15.22	42.55	1.09	90.96	3.85	78.56	20.56
iJET^2 (2/10)	42.72	29.73	68.59	1.96	94.13	7.12	84.06	**24.92**
iJET^2 (8/10)	39.3	28.12	**71.17**	2.04	**95.33**	6.51	**84.19**	24.88
VORFFIP (p>0.5)	**44.32**	**31.17**	68.24	**3.8**	94.3	**7.45**	84.16	11.37
**Heterodimers**								
iJET (7/10)	33.32	14.16	51.68	1.19	**86.91**	6.06	73.03	18.82
iJET^2 (2/10)	**52.63**	**25.45**	61.99	1.58	80.08	**7.27**	75.19	19.45
iJET^2 (8/10)	42.92	23.01	**67.77**	**1.7**	86.31	6.23	**75.55**	**20.78**
**Heterodimers***								
iJET (7/10)	34.43	18.97	51.25	1.19	90.3	5.76	78.2	22.14
iJET^2 (2/10)	**52.97**	30.05	61.53	1.54	85.44	8.37	78.66	23.81
iJET^2 (8/10)	37.42	26.34	**73.06**	1.84	**95.96**	7.03	82.92	**25.74**
VORFFIP (>0.5)	52.72	**34.27**	68.48	**3.23**	91.74	**10.18**	**83.65**	15.36
**Transients**								
iJET (7/10)	37.52	15.99	56.24	1.19	**86.23**	7.76	72.6	20.64
iJET^2 (2/10)	**53.31**	21.85	62.9	**1.64**	75.95	7.42	74.85	19.02
iJET^2 (8/10)	46.98	**23.43**	**64.97**	1.49	85.84	**9.39**	**76.14**	**22.63**
**Transients***								
iJET (7/10)	42.95	21.31	60.08	1.35	**86.24**	7.88	76.28	20.24
iJET^2 (2/10)	**55.12**	24.25	61.46	1.96	75.34	6.21	75.57	15.97
iJET^2 (8/10)	50.57	**27.77**	62.75	1.61	85.75	8.54	76.65	**20.28**
VORFFIP (>0.5)	51.29	26.91	**68.6**	**3.52**	86.2	**10.58**	**80.99**	12.24
**All**								
iJET (7/10)	31.96	15.12	49.32	1.14	88.68	5.52	75	20.37
iJET^2 (2/10)	**47.92**	**26.39**	65.89	1.75	85.88	**7.41**	78.89	22.14
iJET^2 (8/10)	41.69	25	**69.13**	**1.79**	**90.52**	7.21	**79.25**	**23.3**
**All***								
iJET (7/10)	31.87	16.94	47.17	1.15	89.97	4.9	78.08	20.74
iJET^2 (2/10)	46.6	28.74	66.18	1.9	89.27	7.14	81.65	23.06
iJET^2 (8/10)	41.14	27.78	**69.87**	1.93	**93.62**	6.97	82.58	**24.14**
VORFFIP (>0.5)	**46.9**	**30.83**	68.34	**3.66**	92.39	**8.45**	**83.49**	12.13

For iJET, predictions were obtained from a consensus of 7 iterations over 10. For iJET^2, 2 and 8 iterations over 10 were considered. For VORFFIP, predicted patches were defined as formed by residues with probability above 0.5. For each class of proteins, the best performance values are highlighted in bold. The proteins belonging to VORFFIP training set were removed for its evaluation and comparison with iJET and iJET^2 (starred subsets).
