# Supplementary material for: Local Geometry and Evolutionary Conservation of Protein Surfaces Reveal the Multiple Recognition Patches in Protein-Protein Interactions
Source: PLoS Comput Biol. 2015 Dec 21;11(12):e1004580. doi: 10.1371/journal.pcbi.1004580 (PMC4686965; doi:10.1371/journal.pcbi.1004580)
Supplement: S7 Table — (PDF) [file pcbi.1004580.s007.pdf]

	**Sen**	**ScSen**	**PPV**	**ScPPV**	**Spe**	**ScSpe**	**Acc**	**ScAcc**
**Antibody-Antigen**								
iJET (7/10)	10.16	-6.15	9.53	0.42	82.7	-0.99	75.43	4.5
iJET^2 (2/10)	**52.6**	**32.11**	**40**	**2.48**	82.73	**3.21**	79.96	**11.03**
iJET^2 (8/10)	49.86	30.38	37.75	2.4	**83.44**	2.92	**80.19**	10.33
**Antibody-Antigen***								
iJET (7/10)	9.47	-5.86	7.23	0.41	83.65	-1.02	77.49	3.87
iJET^2 (2/10)	**53.28**	**36.15**	39.06	2.86	85.83	**2.96**	83.45	**10.92**
iJET^2 (8/10)	51.45	34.84	37.31	2.79	86.19	2.8	**83.52**	9.97
VORFFIP (p>0.5)	37.21	23.51	**39.61**	**5.96**	**88.26**	1.96	83.42	3.53
**Bound Antibody-Antigen**								
iJET (7/10)	15.74	2.41	14.45	0.98	86.26	-0.41	79.83	6.02
iJET^2 (2/10)	**58.36**	**42.88**	49.15	3.78	88.4	3.88	85.97	12.93
iJET^2 (8/10)	57.3	42.74	**50.17**	**3.83**	**89.41**	**3.97**	**86.73**	**13.33**
**Bound Antibody-Antigen***								
iJET (7/10)	11.36	1.45	11.43	1.04	89.5	-0.59	83.51	5.9
iJET^2 (2/10)	**60.52**	**47.23**	50.6	4.8	90.09	3.39	88.54	12.14
iJET^2 (8/10)	58.94	46.79	**51.53**	4.86	**91.29**	**3.44**	**89.47**	**12.48**
VORFFIP (p>0.5)	52.23	38.68	41.02	**6.76**	89	2.56	85.66	4.81
**Enzyme-Inhibitor**								
iJET (7/10)	40.03	16.73	35.49	1.15	**78.58**	3.8	74.34	12.58
iJET^2 (2/10)	**64.69**	**31.35**	44.81	1.62	73.23	6.57	75.35	13.99
iJET^2 (8/10)	59.19	30.83	**49.11**	**1.77**	78.31	**6.67**	**78.41**	**15.55**
**Enzyme-Inhibitor***								
iJET (7/10)	42.45	19.33	38.97	1.28	81.51	4.64	77.08	14.19
iJET^2 (2/10)	**64.95**	**32.25**	43.36	1.58	74.15	6.84	75.92	14.59
iJET^2 (8/10)	58.5	31.12	46.79	1.69	79.47	6.85	79.08	**16.13**
VORFFIP (p>0.5)	51.59	30.41	**50.13**	**3.21**	**86.07**	**7.25**	**80.31**	10.54
**Other**								
iJET (7/10)	37.17	17.58	29.53	1.1	82.11	3.72	77.3	12.66
iJET^2 (2/10)	**55.75**	**32.37**	42.24	1.83	82.22	**5.6**	80.43	15.01
iJET^2 (8/10)	49.34	30.28	**45.26**	**1.94**	**86.5**	5.56	**83.24**	**15.93**
**Other***								
iJET (7/10)	38.35	17.86	28.78	1.04	81.12	4.05	76.95	12.51
iJET^2 (2/10)	**55.21**	**32.24**	43.16	1.85	82.71	5.68	80.95	14.88
iJET^2 (8/10)	50.48	30.78	**45.45**	1.93	**86.11**	**5.81**	**83.39**	**15.78**
VORFFIP (p>0.5)	37	18.75	31.14	**2.2**	85.23	3.48	79.4	5.52
**All**								
iJET (7/10)	34.56	14.54	28.79	1.06	81.39	3.12	76.46	11.58
iJET^2 (2/10)	**58.34**	**32.77**	43.31	1.95	80.02	**5.59**	79.27	14.27
iJET^2 (8/10)	52.83	31.3	**46.18**	**2.05**	**84.05**	5.59	**81.83**	**15.22**
**All***								
iJET (7/10)	34.84	14.86	28.69	1.06	82.19	3.4	77.61	11.73
iJET^2 (2/10)	**58.63**	**33.88**	43.51	2.11	80.87	5.62	80.2	14.21
iJET^2 (8/10)	53.87	32.63	**45.7**	2.18	84.44	**5.68**	**82.54**	**15.11**
VORFFIP (p>0.5)	42.97	24.59	38.75	**3.24**	**86.09**	4.46	80.58	6.88

The legend is the same as in S6 Table.
