# Supplementary material for: Local Geometry and Evolutionary Conservation of Protein Surfaces Reveal the Multiple Recognition Patches in Protein-Protein Interactions
Source: PLoS Comput Biol. 2015 Dec 21;11(12):e1004580. doi: 10.1371/journal.pcbi.1004580 (PMC4686965; doi:10.1371/journal.pcbi.1004580)
Supplement: S8 Table — (PDF) [file pcbi.1004580.s008.pdf]

Homodimers																
	**iJET**								**iJET^2**							
**Protein**	**Sens**	**ScSens**	**PPV**	**ScPPV**	**Spe**	**ScSpe**	**Acc**	**ScAcc**	**Sens**	**ScSens**	**PPV**	**ScPPV**	**Spe**	**ScSpe**	**Acc**	**ScAcc**
1BNC:A	12.82	3.35	12.2	0.59	90.86	0.33	83.83	9.94	**26.83**	23.53	**78.57**	3.53	**99.22**	2.52	**92.22**	16.33
1DAA:B	46.88	31.71	71.43	1.44	94.37	9.53	83.39	33.12	**62.5**	43.37	**75.47**	1.56	93.9	13.03	**86.64**	35.71
1DPG:B	15.38	7.34	25.64	0.85	93.1	1.14	82.68	16.09	**36.92**	30.33	**75**	2.61	**98.1**	4.69	**89.9**	21.46
1ECP:B	33.33	18.57	77.14	1.12	94.87	9.64	73.84	36.98	**48.15**	29.58	**88.64**	1.29	**96.79**	15.36	**80.17**	41.89
1EFU:D	34.44	18.13	67.39	1.26	92.19	8.5	73.76	26.17	21.11	11.54	**70.37**	1.31	**95.83**	5.41	71.99	24.87
1FRP:A	31.43	19.59	57.89	1.27	93.63	5.46	80.06	26.59	**47.14**	30.01	**60**	1.32	91.24	8.37	**81.62**	28.63
1FUQ:A	43.14	36.12	68.75	2.99	97.53	4.55	**91.45**	18.21	17.65	15.45	**90**	3.81	**99.75**	1.95	90.55	15.32
1GDH:B	28.75	15.62	54.76	1.13	92.08	5.21	76.25	25.13	**46.25**	32.81	**86.05**	1.77	**97.5**	10.94	**84.69**	33.58
1GES:A	13.04	5.9	37.5	0.88	94.38	1.52	77.68	21.5	**50**	37.31	**78.95**	1.9	**96.66**	9.35	**87.31**	30.94
1GLQ:A	52.94	40.5	69.23	2.24	95.43	7.87	88.52	24.18	50	39.95	**80.95**	2.6	**97.71**	7.76	**89.95**	24.92
1GP1:B	**70**	45	30.43	1.4	80.49	5.49	79.35	15.22	**85**	59.46	**36.17**	1.68	**81.71**	7.25	**82.07**	18.13
1GPM:D	3.53	-4.09	7.89	0.22	91.55	-0.84	76.55	15.23	**35.29**	26.08	**65.22**	1.85	**96.14**	5.35	**85.77**	23.76
1HUR:B	50	30.56	22.86	1.3	83.54	2.98	80.56	10.74	**62.5**	40.28	**25**	1.42	81.71	3.93	80	11.99
1HYH:B	10.34	-0.54	28.12	0.44	88.89	-0.23	65.65	26.21	**33.72**	16.21	**55.77**	0.89	**89.1**	6.61	**73.06**	31.07
1IDS:C	27.5	12.35	73.33	1.06	93.22	8.37	66.67	29.89	**36.25**	15.04	69.05	1	88.98	10.2	**67.68**	28.6
1IES:B	29.41	8.15	67.57	0.75	86.52	7.78	58.62	31.85	**55.29**	19.66	**75.81**	0.84	83.15	18.78	**69.54**	31.16
1LEH:B	5.88	-5.66	4.76	0.24	87.88	-0.58	80.22	7.05	**55.88**	50.11	**90.48**	4.66	**99.39**	5.16	**95.33**	18.29
1MAS:A	40.74	28.73	29.73	1.5	90.75	2.76	86.36	13.46	**82.76**	64.11	**43.64**	2.05	88.35	6.99	**87.8**	19.81
1MLD:B	38.98	24.93	52.27	1.25	91.73	5.79	81.79	25.93	33.9	25.27	**74.07**	1.83	**97.24**	5.87	**85.3**	27.37
1NHK:L	15.15	-7.93	15.15	0.35	74.55	-2.38	60.84	6.99	**39.39**	25.41	**65**	1.52	**93.64**	7.62	**81.12**	25.97
1NQV:A	36.84	13.47	77.78	0.84	89.74	13.12	63.64	36.36	**56.58**	20.22	76.79	0.83	83.33	19.7	**70.13**	31.77
1ORO:B	44.44	22.11	34.78	1.03	82.35	4.68	75.73	16.68	41.67	32.93	**83.33**	2.48	**98.24**	6.97	**88.35**	24.85
1OSJ:A	32.2	15.39	32.76	0.91	86.36	3.18	77.1	17.79	**37.29**	28.88	**75.86**	2.03	**97.55**	5.96	**87.25**	26.72
1PKY:A	22.81	13.11	28.89	1.13	92.14	1.84	83.62	14.01	**42.37**	32.21	**56.82**	1.9	**94.92**	5.08	**87.76**	21.78
1POY:1	28.95	17.49	29.73	1.17	90.88	2.33	83.59	14.58	7.89	6.66	**75**	2.96	**99.65**	0.89	**88.85**	14.8
1QOR:A	18.18	8.37	18.75	0.89	91.13	0.94	83.74	10.56	**48.48**	42.66	**84.21**	3.98	**98.98**	4.8	**93.87**	18.38
1RAH:D	19.23	-0.38	33.33	0.6	80.2	-0.19	59.48	13.07	**30.77**	18.35	**84.21**	1.57	**97.03**	9.45	**74.51**	27.22
1SCU:B	27.78	19.27	75.76	1.75	97.32	5.82	81.19	25.6	**33.33**	23.28	**76.92**	1.71	96.98	7.03	**82.22**	28.22
1SET:A	15.62	7.31	28.57	0.99	93	1.31	81.24	13.58	**17.19**	11.01	**42.31**	1.41	**95.8**	1.97	**83.85**	16.24
1SFT:B	46.59	33.96	85.42	1.67	**97.6**	10.23	85.79	36.66	**55.68**	41.47	**90.74**	1.77	**98.29**	12.5	**88.42**	39.25
1TPH:2	48.89	33.38	57.89	1.47	92	7.51	84.08	26.82	**68.89**	41.54	46.27	1.23	82	9.35	79.59	23.93
1XIK:B	26.03	17.23	63.33	1.31	95.9	4.69	80.94	29.57	8.22	5.87	**75**	1.55	**99.25**	1.6	79.77	28.19
2CST:A	14.14	7.57	51.85	1.02	95.83	2.4	76.16	27.05	**44.44**	30.58	**77.19**	1.51	95.83	9.7	**83.45**	34.2
2EIP:A	36.84	15.41	19.44	0.89	80.54	1.97	75.6	9.5	**47.37**	36.39	**47.37**	2.27	**93.51**	4.49	**88.44**	15.71
2HHM:A	30	18.97	50	1.26	93.24	4.27	81.62	23.58	**36**	26.07	**66.67**	1.68	**95.95**	5.87	**84.93**	26.66
2PCD:M	22.64	5.47	**90**	0.75	94.59	11.76	45.49	41.76	**40.88**	7.4	83.33	0.69	82.43	15.91	**54.08**	27.36
2POL:A	0	-8.74	0	0	90.42	-0.84	82.51	5.61	**53.12**	38.64	**32.08**	1.74	89.22	3.7	**86.07**	13.61
3LAD:B	14.15	5.68	37.5	0.81	93.17	1.64	75.42	22.17	**39.62**	30.09	**93.33**	2.03	**99.18**	8.71	**85.81**	32.64
3MDE:A	34.62	25.78	52.94	1.76	95.2	4.03	87.01	20.59	**42.31**	31.92	**55**	1.76	94.59	4.98	**87.53**	22.74
6GSV:B	33.33	20.43	46.43	1.3	91.57	4.48	81.11	20.56	**58.97**	40.54	**57.5**	1.68	90.45	8.88	**84.79**	24.82
8CAT:A	14.88	6.84	45	1.01	94.16	2.2	74.9	20.27	11.57	8.36	**87.5**	1.97	**99.47**	2.68	**78.11**	22.95
**All**	**28.58**	**15.28**	**44.74**	**1.09**	**90.85**	**4.15**	**77.27**	**21.14**	**42.66**	**29.04**	**69.55**	**1.91**	**93.87**	**7.5**	**82.94**	**25.17**

Consensus predictions were obtained from 7 and 2 iterations over 10 for iJET and iJET^2. The reported values correspond to the best predicted patch. Values highlighted in bold correspond to improved performance of iJET^2 over iJET. Maximum values in each column are highlighted in blue and bold. Average values computed on all proteins from the ensemble are reported in the last row. Rows highlighted in green indicate very good iJET^2 predictions ($Sens > 60$, $PPV > 60$, $Spe > 80$, $Acc > 80$). Rows highlighted in red indicate very bad iJET^2 predictions ($Sens = 0$).
