# Supplementary material for: Local Geometry and Evolutionary Conservation of Protein Surfaces Reveal the Multiple Recognition Patches in Protein-Protein Interactions
Source: PLoS Comput Biol. 2015 Dec 21;11(12):e1004580. doi: 10.1371/journal.pcbi.1004580 (PMC4686965; doi:10.1371/journal.pcbi.1004580)
Supplement: S9 Table — (PDF) [file pcbi.1004580.s009.pdf]

Heterodimers																
	**iJET**								**iJET^2**							
**Protein**	**Sens**	**ScSens**	**PPV**	**ScPPV**	**Spe**	**ScSpe**	**Acc**	**ScAcc**	**Sens**	**ScSens**	**PPV**	**ScPPV**	**Spe**	**ScSpe**	**Acc**	**ScAcc**
1ALL:A	27.03	8.28	33.33	0.83	83.74	2.49	70.62	14.51	**64.86**	24.24	**36.92**	0.92	66.67	7.29	66.25	14.42
1ALL:B	38.89	15.76	37.84	1.06	81.45	4.58	71.88	14.16	**63.89**	18.26	31.51	0.88	59.68	5.3	60.62	9.37
1HCG:A	18.18	4.2	18.18	0.59	86.7	0.68	77.12	13.32	**30.3**	26.07	**100**	3.24	**100**	4.24	**90.25**	22.72
1HCG:B	**69.57**	8.78	51.61	0.9	46.43	7.21	56.86	5.25	**95.65**	-2.39	44	0.77	0	-1.96	43.14	-14.07
1LUC:A	15.79	12.42	81.82	2.12	**99.26**	2.63	84.66	23.95	**49.12**	35.01	60.87	1.58	93.31	7.42	**85.58**	27.34
1LUC:B	51.72	40.47	83.33	2.1	97.71	8.96	89.38	31.41	**65.52**	48.33	69.09	1.74	93.51	10.7	88.44	31.7
1SCU:D	42.42	28.84	71.79	1.38	95.02	8.61	82.93	34.36	**59.09**	40.97	**75**	1.44	94.12	12.24	**86.06**	37.32
1SCU:E	26.67	18.42	75	1.67	97.32	5.56	80.93	26.75	**33.33**	23.28	**76.92**	1.71	96.98	7.03	**82.22**	28.22
1TCO:A	17.39	8.3	25	0.87	92.16	1.25	82.39	15	**39.13**	34.02	**100**	3.48	**100**	5.11	**92.05**	22.97
1TCO:B	41.27	17.6	65	1.1	86.79	10.46	69.82	24.5	**57.14**	18.68	55.38	0.94	72.64	11.1	66.86	18.91
1TCO:C	37.5	-0.82	21.95	0.54	61.45	-0.24	56.07	3.9	**79.17**	18.42	**29.23**	0.72	44.58	5.33	52.34	4.34
1TCR:A	30.16	15.8	65.52	1.4	92.81	7.16	73.27	20.89	**47.62**	19.9	53.57	1.15	81.29	9.02	70.79	19.31
1TCR:B	45.31	27.09	67.44	1.42	91.86	10.08	79.24	27.59	**53.12**	35.33	**80.95**	1.71	**95.35**	13.15	**83.9**	32.23
1UBS:A	65	47.88	59.09	1.76	91.71	8.83	87.55	26.77	**75**	55.16	58.82	1.75	90.32	10.17	**87.94**	28.05
1UBS:B	0	-10.28	0	0	88.51	-1.21	79.18	12.08	**26.83**	23.23	**78.57**	2.76	**99.14**	2.74	**91.52**	21.54
1WDC:A	43.86	3.23	**96.15**	1.06	85.71	26.34	48.44	6.03	38.6	-2.03	84.62	0.94	42.86	-16.52	39.06	-3.35
1WDC:B	42.11	14.64	61.54	1.13	82.35	9.82	66.2	18.13	**66.67**	15.96	52.78	0.97	60	10.7	62.68	12.62
1WDC:C	26.53	10.08	52	1.1	88.35	4.8	68.42	16.49	**40.82**	13.84	48.78	1.04	79.61	6.59	67.11	15.78
2PCD:B	33.71	13.71	75	0.96	90.99	10.99	65.5	32.34	**75.28**	33.78	**80.72**	1.03	85.59	27.09	**81**	35.77
2PCD:N	20.75	4.87	89.19	0.73	94.59	10.47	44.21	43.55	**35.85**	7.09	85.07	0.7	86.49	15.24	**51.93**	32.65
8ATC:A	12.5	-1.05	9.52	0.41	86.33	-0.12	78.71	9.29	9.38	5.5	**25**	1.07	**96.76**	0.63	**87.74**	13.16
8ATC:B	22.5	2.64	31.03	0.71	81.13	1	65.07	11.14	**57.5**	35.58	**71.88**	1.65	**91.51**	13.43	**82.19**	28.53
9ATC:A	13.64	0.73	7.5	0.47	87.15	0.06	81.94	6.58	13.64	9.12	**21.43**	1.35	**96.18**	0.7	**90.32**	9.24
9ATC:B	57.14	48.24	61.54	4.13	96.21	5.12	**92.47**	13.61	**85.71**	73.39	**66.67**	4.48	95.45	7.78	**94.52**	18.07
**All**	**33.32**	**14.16**	**51.68**	**1.19**	**86.91**	**6.06**	**73.03**	**18.82**	**52.63**	**25.45**	**61.99**	**1.58**	**80.08**	**7.27**	**75.19**	**19.45**

The legend is the same as in S8 Table.
