# Supplementary material for: Local Geometry and Evolutionary Conservation of Protein Surfaces Reveal the Multiple Recognition Patches in Protein-Protein Interactions
Source: PLoS Comput Biol. 2015 Dec 21;11(12):e1004580. doi: 10.1371/journal.pcbi.1004580 (PMC4686965; doi:10.1371/journal.pcbi.1004580)
Supplement: S10 Table — (PDF) [file pcbi.1004580.s010.pdf]

Transients																
	**iJET**								**iJET^2**							
**Protein**	**Sens**	**ScSens**	**PPV**	**ScPPV**	**Spe**	**ScSpe**	**Acc**	**ScAcc**	**Sens**	**ScSens**	**PPV**	**ScPPV**	**Spe**	**ScSpe**	**Acc**	**ScAcc**
1APM:E	41.46	32.08	53.12	2.05	95	4.38	88.56	19.03	26.83	22.14	**68.75**	2.65	**98.33**	3.03	**89.74**	17.94
1EFU:A	21.69	13.99	64.29	1.49	96.44	4.13	79.4	23.48	**30.12**	19.96	**67.57**	1.57	95.73	5.89	**80.77**	25.2
1EFU:B	22.22	12.29	71.43	1.37	95.83	5.76	72.34	23.96	**33.33**	14.54	56.6	1.09	88.02	6.82	70.57	21.83
1G3N:A	14.14	2.2	40	0.61	89.18	1.12	63.82	25.67	**33.33**	15.93	**64.71**	0.99	**90.72**	8.13	**71.33**	31.48
1G3N:B	27.59	-0.16	18.6	0.54	72.22	-0.04	63.87	6.98	24.14	-15.22	11.48	0.33	57.14	-3.5	50.97	-2.33
1G3N:C	32.61	15.44	37.5	0.94	86.63	3.8	75.97	19.4	**71.74**	44.7	**52.38**	1.31	83.96	11	**81.55**	26.95
1GOT:A	50	40.24	54.55	2.68	95.03	4.8	**90.24**	16.37	**58.33**	50.35	**77.78**	3.82	**98.01**	6	**93.79**	18.86
1GOT:B	5.13	2.47	66.67	0.91	**98.65**	1.3	66.37	38.27	**28.21**	13.16	64.71	0.88	91.89	6.94	**69.91**	36.08
1GOT:G	68.09	9.46	**94.12**	1.12	81.82	40.44	70.69	14.84	**97.87**	1.32	82.14	0.98	9.09	5.64	**81.03**	-0.55
1K9O:E	45.71	31.81	51.61	1.39	92.02	5.92	84.75	25.54	**57.14**	39.21	50	1.34	89.36	7.3	84.3	26.12
1K9O:I	21.05	8.02	8.16	0.74	87.39	0.43	84.04	5.19	**52.63**	49.44	**83.33**	7.59	**99.44**	2.63	**97.07**	10.55
1RRP:A	14.81	-0.38	38.71	0.56	84.55	-0.25	56.86	20.25	**29.63**	12.96	**70.59**	1.02	**91.87**	8.54	**67.16**	29.98
1RRP:B	46.03	16.93	74.36	1.04	85.92	15.02	67.16	26.19	**79.37**	26.38	70.42	0.98	70.42	23.41	**74.63**	23.34
1RRP:C	25	4.44	43.24	0.64	81.9	2.45	61.67	21.89	**39.06**	22.95	**86.21**	1.28	**96.55**	12.66	**76.11**	37.88
1UGH:E	63.64	47.94	60	1.75	92.63	8.33	88.34	27.62	**81.82**	60.74	57.45	1.67	89.47	10.55	88.34	29.3
1UGH:I	42.42	18.03	70	1.17	87.76	12.15	69.51	24.63	**78.79**	12.93	48.15	0.8	42.86	8.71	57.32	4.15
1YTF:A	52.73	32.73	80.56	1.54	94.4	14.4	81.67	33.1	34.55	14.55	52.78	1.01	86.4	6.4	70.56	21.98
1YTF:B	**80**	12.61	64.52	1.06	47.62	15.01	65.22	11.4	**96**	9.04	60	0.98	23.81	10.77	63.04	4.93
1YTF:D	38.57	3.57	77.14	0.95	73.33	8.33	49	8.42	**60**	0	70	0.86	40	0	**54**	-2.28
**All**	**37.52**	**15.99**	**56.24**	**1.19**	**86.23**	**7.76**	**72.6**	**20.64**	**53.31**	**21.85**	**62.9**	**1.64**	**75.95**	**7.42**	**74.85**	**19.02**

The legend is the same as in S8 Table.
