# Supplementary material for: Local Geometry and Evolutionary Conservation of Protein Surfaces Reveal the Multiple Recognition Patches in Protein-Protein Interactions
Source: PLoS Comput Biol. 2015 Dec 21;11(12):e1004580. doi: 10.1371/journal.pcbi.1004580 (PMC4686965; doi:10.1371/journal.pcbi.1004580)
Supplement: S11 Table — (PDF) [file pcbi.1004580.s011.pdf]

Antibody-Antigen																
	iJET								iJET^2							
**Protein**	**Sens**	**ScSens**	**PPV**	**ScPPV**	**Spe**	**ScSpe**	**Acc**	**ScAcc**	**Sens**	**ScSens**	**PPV**	**ScPPV**	**Spe**	**ScSpe**	**Acc**	**ScAcc**
1AHW:L	0	-12.38	0	0	85.55	-2.07	73.27	5.3	**17.24**	-2.07	**12.82**	0.49	80.35	-0.35	71.29	6.63
1AHW:R	0	-3.97	0	0	95.73	-0.3	89.02	6.08	**73.33**	63.52	**52.38**	3.68	94.97	4.79	**93.46**	14.7
1BGX:L	8.14	6.06	**41.18**	1.89	98.63	0.71	89.11	12.11	**10.47**	9.24	**90**	4.12	**99.86**	1.09	**90.45**	12.97
1BGX:R	11.36	5.16	38.46	0.97	95.17	1.37	77.57	18.49	**31.82**	24.02	**84.85**	2.14	**98.51**	6.31	**84.63**	25.89
1BVK:L	18.18	-8.17	11.76	0.38	71.96	-1.68	62.79	3.8	**68.18**	17.79	**23.08**	0.74	53.27	3.66	55.81	5.96
1BVK:R	18.18	-15.3	5.33	0.29	64.85	-1.67	60.27	-0.09	**63.64**	30.6	**18.92**	1.01	**70.3**	3.33	**69.64**	9
1DQJ:L	19.23	-7.13	14.71	0.4	71.84	-1.8	61.24	5.16	**53.85**	-3.52	**18.92**	0.51	41.75	-0.89	44.19	-3.92
1DQJ:R	0	-6.13	0	0	93.43	-0.43	87.26	4.56	**57.14**	50.54	**57.14**	4.49	**96.97**	3.57	**94.34**	11.99
1E4K:L	21.74	10.18	25	1.07	90	1.56	80.92	10.53	**52.17**	44.08	**85.71**	3.65	**98.67**	6.76	**92.49**	20.25
1E4K:R	0	-1.67	0	0	98.23	-0.1	92.6	3.74	**37.5**	31.53	**36**	3.68	95.95	1.92	92.6	7.19
1E6J:L	40	-12.78	15.79	1.42	43.86	-3.36	43.06	-4.78	**93.33**	21.11	**26.92**	2.42	33.33	5.56	**45.83**	13.12
1E6J:R	4.55	-4.31	2.63	0.27	90.91	-0.23	86.48	3.49	**81.82**	71.56	**40.91**	4.13	**93.61**	3.87	**93.01**	11.14
1JPS:L	0	-19.23	0	0	76.97	-3.8	64.29	-0.57	**10**	5.6	**37.5**	1.45	**96.71**	1.11	**82.42**	10.4
1JPS:R	0	-6.35	0	0	93.18	-0.47	86.82	4.9	**72.41**	61.83	**46.67**	3.48	**93.94**	4.53	**92.47**	13.64
1MLC:L	10	-14.81	6.25	0.22	72.48	-2.72	62.79	1.99	**60**	31.32	**32.43**	1.14	**77.06**	5.75	**74.42**	15.28
1MLC:R	0	-5.32	0	0	94.38	-0.3	89.35	3.93	**43.48**	37.92	**41.67**	4.02	**96.58**	2.13	**93.75**	8.51
1VFB:L	8.7	-16.11	6.25	0.2	71.7	-3.5	60.47	0.72	**34.78**	20.05	**42.11**	1.37	**89.62**	4.35	**79.84**	16.21
1VFB:R	15	-20.71	3.75	0.22	62.25	-2.03	58.04	-1.28	**60**	41.7	**29.27**	1.68	**85.78**	4.09	**83.48**	12.81
1WEJ:L	18.75	-7.21	11.11	0.42	72.73	-1.31	64.42	2.99	**50**	-4.81	**14.04**	0.54	44.32	-0.87	45.19	-2.52
1WEJ:R	0	-3.46	0	0	96.34	-0.19	91.22	4.24	**69.57**	60.1	**39.02**	3.8	93.9	3.37	**92.61**	10.4
2FD6:L	5.88	-4.19	3.7	0.29	89.64	-0.28	84.33	4.46	**11.76**	-0.92	**5.88**	0.47	87.25	-0.06	82.46	4.55
2FD6:R	0	-4.52	0	0	95.3	-0.18	91.67	2.9	**93.75**	81.54	**28.85**	3.91	90.98	3.18	91.08	8.86
2I25:L	**41.67**	16.09	30.3	0.91	78.1	3.68	71.32	13.18	**70.83**	15.79	23.94	0.72	48.57	3.61	52.71	4.39
2I25:R	5.26	-34.56	2.22	0.08	53.19	-6.99	45.13	-9.27	**63.16**	45.46	**60**	2.12	**91.49**	9.19	**86.73**	22.74
2VIS:L	12	10.75	25	1.3	**99.04**	0.29	**96.77**	16.77	4	3.69	**33.33**	1.73	**99.79**	0.1	**97.29**	16.71
2VIS:R	5.56	0.22	4.35	0.53	94.67	0.01	90.95	3.56	**83.33**	77.3	**57.69**	7.08	**97.34**	3.37	**96.75**	9.95
**All**	**10.16**	**-6.15**	**9.53**	**0.42**	**82.7**	**-0.99**	**75.43**	**4.5**	**52.6**	**32.11**	**40**	**2.48**	**82.73**	**3.21**	**79.96**	**11.03**

The legend is the same as in S8 Table.
