# Supplementary material for: Local Geometry and Evolutionary Conservation of Protein Surfaces Reveal the Multiple Recognition Patches in Protein-Protein Interactions
Source: PLoS Comput Biol. 2015 Dec 21;11(12):e1004580. doi: 10.1371/journal.pcbi.1004580 (PMC4686965; doi:10.1371/journal.pcbi.1004580)
Supplement: S12 Table — (PDF) [file pcbi.1004580.s012.pdf]

Bound Antibody-Antigen																
	iJET								iJET^2							
**Protein**	**Sens**	**ScSens**	**PPV**	**ScPPV**	**Spe**	**ScSpe**	**Acc**	**ScAcc**	**Sens**	**ScSens**	**PPV**	**ScPPV**	**Spe**	**ScSpe**	**Acc**	**ScAcc**
1BJ1:L	5	-14.58	2.7	0.16	78.7	-1.73	70.9	0.7	**65**	54.42	**65**	3.87	**95.86**	6.44	**92.59**	16.42
1BJ1:R	0	-7.19	0	0	92.35	-0.46	86.77	3.99	**69.23**	63.43	**72**	6.15	**98.27**	4.07	**96.52**	12.67
1FSK:L	**54.55**	37.56	44.44	1.76	89.05	6.03	84.28	17.96	54.55	45.74	**85.71**	3.39	**98.54**	7.35	**92.45**	22.09
1FSK:R	3.7	-0.9	5	0.42	95.33	-0.06	89.63	5.09	**55.56**	47.03	**40.54**	3.39	94.59	3.12	**92.17**	10.6
1I9R:L	4.17	-0.67	4.76	0.41	95.12	-0.04	90.09	5.45	**87.5**	72.29	**31.82**	2.73	89.02	4.23	88.94	12.25
1I9R:R	20	16.8	42.86	2.5	**98.04**	1.24	92.69	11.94	**56.67**	45.25	34	1.98	91.91	3.33	89.5	14.14
1IQD:L	10.53	-10.86	5.88	0.24	77.14	-1.47	69.18	4.87	**42.11**	35.19	**72.73**	2.91	**97.86**	4.78	**91.19**	19.65
1IQD:R	8.33	0.43	9.38	0.54	92.14	0.04	84.69	7.17	**19.44**	17.24	**77.78**	4.49	**99.46**	1.67	**92.4**	11.15
1K4C:L	31.58	22.7	17.14	0.63	92.27	1.15	89.34	20.54	**89.47**	80.08	**45.95**	1.69	**94.67**	4.06	**94.42**	25.85
1K4C:R	0	-6.03	0	0	93.6	-0.37	88.17	4.33	**64**	54.95	**41.03**	3.56	**94.33**	3.38	**92.58**	11.06
1KXQ:L	13.33	-25	8.7	0.19	53.33	-8.33	43.33	-7.56	**53.33**	30.83	**59.26**	1.28	**87.78**	10.28	**79.17**	27.05
1KXQ:R	30.56	26.32	**52.38**	2.91	97.83	2.06	92.94	13.65	**41.67**	36.02	**53.57**	2.98	97.17	2.82	**93.15**	14.76
1NCA:L	10.34	2.81	10.34	0.57	92.7	0.23	86.49	9.52	10.34	2.61	10	0.55	92.48	0.21	86.34	9.49
1NCA:R	15.15	9.78	21.74	1.44	95.44	0.82	89.25	8.08	**75.76**	62.88	**44.64**	2.96	92.29	5.16	**91.03**	15.1
1NSN:L	16.67	-4.5	17.24	0.44	77.57	-1.26	64.23	7.87	**40**	-8.91	**17.91**	0.46	48.6	-2.5	46.72	-3.53
1NSN:R	12.9	5.27	12.5	0.88	92.78	0.42	86.87	6.61	**74.19**	60.84	**40.35**	2.82	91.41	4.76	**90.16**	13.98
1QFW:L	34.78	10.8	17.02	0.87	77.46	1.44	72.45	6.57	**65.22**	50.93	**53.57**	2.75	**92.49**	6.77	**89.29**	17.49
1QFW:R	18.52	-13.8	6.76	0.3	65.84	-1.84	60.26	0.67	**51.85**	14.73	**16.47**	0.72	64.85	1.97	**63.32**	6.33
2HMI:L	0	-6.91	0	0	92.7	-0.39	87.79	3.59	**78.26**	66.51	**35.29**	3.42	91.97	3.72	**91.24**	10.89
2HMI:R	37.5	34.95	24	6.8	98.03	0.58	**97.04**	2.94	37.5	36.89	**100**	28.31	**100**	0.61	**98.98**	3.08
2JEL:L	9.52	-24.59	6.9	0.17	57.81	-8.07	45.88	-6.92	**76.19**	3.25	**25.81**	0.63	28.12	1.07	40	-5.95
2JEL:R	6.9	3.6	14.29	1.08	96.97	0.26	90.82	6.43	**75.86**	66.21	**52.38**	3.97	95.07	4.73	**93.79**	14.08
2QFW:L	30	14.69	20	1.18	86.36	1.67	80.61	7.68	**65**	54.8	**65**	3.84	**96.02**	6.23	**92.86**	16.55
2QFW:R	3.7	-12.89	2.7	0.13	81.63	-1.78	72.2	3.22	**51.85**	35.78	**38.89**	1.8	**88.83**	4.9	**84.38**	15.1
**All**	**15.74**	**2.41**	**14.45**	**0.98**	**86.26**	**-0.41**	**79.83**	**6.02**	**58.36**	**42.88**	**49.15**	**3.78**	**88.4**	**3.88**	**85.97**	**12.93**

The legend is the same as in S8 Table.
