# Supplementary material for: Local Geometry and Evolutionary Conservation of Protein Surfaces Reveal the Multiple Recognition Patches in Protein-Protein Interactions
Source: PLoS Comput Biol. 2015 Dec 21;11(12):e1004580. doi: 10.1371/journal.pcbi.1004580 (PMC4686965; doi:10.1371/journal.pcbi.1004580)
Supplement: S13 Table — (PDF) [file pcbi.1004580.s013.pdf]

Enzyme-Inhibitor																
	**iJET**								**iJET^2**							
**Protein**	**Sens**	**ScSens**	**PPV**	**ScPPV**	**Spe**	**ScSpe**	**Acc**	**ScAcc**	**Sens**	**ScSens**	**PPV**	**ScPPV**	**Spe**	**ScSpe**	**Acc**	**ScAcc**
1ACB:L	72.22	22.22	37.14	0.89	57.69	7.69	61.43	11.43	**94.44**	28.73	36.96	0.88	44.23	9.95	57.14	9.7
1ACB:R	64.52	54.31	80	2.86	97.66	7.87	93.47	25.9	**74.19**	51.34	41.07	1.47	84.58	7.44	83.27	21.28
1AVX:L	0	-16.57	0	0	81.21	-2.22	71.6	2.7	**55**	45.53	**68.75**	3.16	**96.64**	6.11	**91.72**	18.81
1AVX:R	44.12	30.94	51.72	1.57	92.47	5.65	85	22.49	**73.53**	54.25	**58.14**	1.76	90.48	9.76	**87.89**	27.45
1AY7:L	82.35	56.51	60.87	1.86	87.5	13.34	86.52	28.15	**94.12**	54.79	45.71	1.4	73.61	12.94	77.53	23.83
1AY7:R	55	17.5	30.56	0.92	67.11	4.61	64.58	10.42	**90**	37.92	**36**	1.08	57.89	9.98	64.58	15.28
1BVN:L	75	31.76	65.62	1.17	76.09	19.33	75.68	26.49	**85.71**	38.42	**68.57**	1.22	76.09	23.38	**79.73**	30.05
1BVN:R	41.86	37.62	85.71	4.03	99.34	3.58	**94.34**	18.07	**51.16**	45.91	84.62	3.97	99.12	4.37	**94.95**	19.25
1CGI:L	66.67	13.1	46.67	0.84	54.29	7.86	58.93	8.55	**100**	17.86	45.65	0.83	28.57	10.71	55.36	1.97
1CGI:R	51.35	41.56	79.17	2.31	97.6	7.39	90.61	27.96	**54.05**	38.95	54.05	1.58	91.83	6.93	86.12	25.14
1CLV:L	0	0	0	0	0	0	0	0	**95.65**	8.15	**78.57**	0.82	**33.33**	20.83	**78.12**	-6.25
1CLV:R	48.72	42.76	67.86	3.46	97.91	3.87	93.83	17.05	**58.97**	51.1	62.16	3.17	96.75	4.62	93.62	18
1D6R:L	40	-1.38	25	0.68	58.14	-0.48	53.45	1.14	**100**	10.34	**28.85**	0.79	13.95	3.61	36.21	-3.15
1D6R:R	43.75	27.84	40	1.28	88.83	4.74	82.27	19.57	**68.75**	49.47	**51.16**	1.63	**89.01**	8.29	**86.1**	24.65
1DFJ:L	18	16.03	**100**	4.4	**100**	1.97	91.01	14.81	18	12.3	34.62	1.52	95.81	1.51	87.28	13.12
1DFJ:R	42.5	15.89	51.52	0.95	80.95	7.57	68.55	20.44	**85**	31.77	51.52	0.95	61.9	15.13	**69.35**	19.09
1E6E:L	66.67	35.83	54.55	1.37	81.25	12.09	77.57	23.63	**92.59**	39.32	43.86	1.1	60	13.27	68.22	18.9
1E6E:R	30	20.77	21.43	1.5	92.24	1.47	88.13	9.01	**60**	52.09	**50**	3.5	**95.76**	3.68	**93.41**	13.34
1EAW:L	65.22	16.94	53.57	0.95	62.86	11.13	63.79	14	**95.65**	6	42.31	0.75	14.29	3.94	46.55	-8.28
1EAW:R	15	-3.06	14.63	0.4	81.28	-0.65	69.6	11.11	**60**	43.82	**61.54**	1.68	**92.54**	8.72	**87.14**	28.14
1EWY:L	60.87	26.18	41.18	1.02	73.33	8.03	70.41	17.45	**82.61**	24.45	33.33	0.83	49.33	7.5	57.14	8.72
1EWY:R	34.78	22.58	22.22	1.43	89.71	1.91	85.42	9.37	**65.22**	43.52	**23.44**	1.51	81.99	3.68	80.68	11.17
1EZU:L	17.14	8.69	25	1.19	92.77	1.22	83.45	9.32	**60**	37.46	**32.81**	1.57	82.73	5.27	79.93	13.98
1EZU:R	37.78	21.34	47.22	1.03	89.08	5.52	78.54	25.8	**60**	36.23	**50.94**	1.11	85.39	9.16	**80.27**	28.13
1F34:L	19.44	-11.26	17.95	0.45	64.84	-4.46	51.97	-2.06	**44.44**	24.76	**64**	1.62	**90.11**	9.79	**77.17**	20.84
1F34:R	25.45	13.8	36.84	1	91.14	2.8	80.06	20.03	25.45	16.56	**48.28**	1.31	**94.46**	3.36	**82.82**	22.06
1F6M:L	65.52	34.96	57.58	1.21	82.28	12.83	77.78	26.82	**86.21**	50.1	**64.1**	1.35	82.28	18.39	**83.33**	32.65
1F6M:R	33.33	23.87	30	1.88	92.76	2.22	87.7	10.11	33.33	23.87	30	1.88	92.76	2.22	87.7	10.11
1FLE:L	55	-0.32	42.31	0.68	44.44	-0.24	48.94	-2.39	**90**	0.64	**42.86**	0.69	11.11	0.47	44.68	-15.16
1FLE:R	6.67	-4.69	7.69	0.28	87.94	-0.71	77.29	9.92	**53.33**	36.67	**40**	1.45	**88.57**	5.24	**84.17**	19.18
1FQ1:L	13.64	-0.16	7.5	0.49	86.19	-0.01	80.69	5.47	**54.55**	24.2	**13.64**	0.9	71.64	1.99	70.34	6.65
1FQ1:R	60	39.78	41.67	1.57	86.27	6.5	82.58	18.65	**84**	48.04	32.81	1.23	71.9	7.85	73.6	17.02
1GL1:L	75	-4.41	55.56	0.75	14.29	-6.3	50	-14.16	**85**	-0.29	**58.62**	0.79	14.29	-0.42	**55.88**	-11.11
1GL1:R	60	48.56	66.67	2.47	95.63	7.07	91.1	23.39	**70**	55.17	60	2.22	93.2	8.03	90.25	24.1
1GXD:L	0	-13.74	0	0	84.08	-2.19	72.53	1.37	**72**	58.81	**75**	3.6	**96.18**	9.37	**92.86**	21.38
1GXD:R	0	-1.94	0	0	97.93	-0.13	91.6	5.74	**20**	18.06	**66.67**	5.25	**99.31**	1.25	**94.18**	8.33
1HIA:L	**94.44**	25.06	50	1	45.16	14.55	63.27	13.27	66.67	34.01	**75**	1.5	**87.1**	19.75	**79.59**	29.59
1HIA:R	15.15	-8.17	9.62	0.31	75.26	-1.42	66.37	6.46	**63.64**	32.17	**28.77**	0.92	73.87	5.33	**72.41**	15.53
1IJK:L	5	-1.73	7.14	0.34	93.09	-0.18	84.62	9.38	0	-6.25	0	0	93.09	-0.66	84.13	8.61
1IJK:R	15	-7.83	5.17	0.33	76.5	-0.67	71.65	2.98	**50**	9.06	**9.62**	0.62	59.83	0.77	59.06	2.83
1JIW:L	52.63	24.35	35.71	1.18	77.5	5.78	72.73	14.11	**84.21**	29.67	29.63	0.98	52.5	7.05	58.59	10.39
1JIW:R	29.73	20.79	26.19	1.6	92.84	1.78	87.87	10.25	29.73	21.01	**26.83**	1.64	**93.07**	1.79	**88.09**	10.32
1JTG:L	27.78	-1.31	20.83	0.52	70.54	-0.37	61.21	7.22	19.44	10.35	**46.67**	1.15	**93.8**	2.89	**77.58**	19.76
1JTG:R	43.59	31.04	51.52	1.57	92.86	5.4	85.55	22.65	**46.15**	27.14	36	1.1	85.71	4.73	79.85	19.17
1KKL:L	64	32.97	59.26	1.21	82.26	13.29	77.01	26.64	**88**	37.43	50	1.02	64.52	15.09	71.26	21.28
1KKL:R	21.74	16.22	19.23	1.91	96.65	2.17	92.99	7.45	**78.26**	70.39	**47.37**	4.72	95.65	3.52	**94.82**	11.15
1M10:L	0	-5.26	0	0	93.94	-0.8	81.58	9.37	**25.71**	13.31	**27.27**	1.08	89.61	2.02	81.2	12.54
1M10:R	17.65	8.99	33.33	0.92	93.1	1.76	80.77	19.33	**32.35**	9.28	22.92	0.63	78.74	1.81	71.15	13.71
1MAH:L	52.38	3.2	36.67	0.79	52.5	1.68	52.46	2.4	**100**	6.56	**36.84**	0.79	10	3.44	40.98	-6.12
1MAH:R	13.16	9.22	23.81	1.3	96.77	0.71	90.81	11.58	**15.79**	12.6	**35.29**	1.93	**97.78**	0.97	**91.93**	12.23
1N8O:L	0	-20.44	0	0	76.07	-3.49	64.96	0.18	**55**	43.57	**68.75**	2.75	**95.83**	7.26	**90**	20.71
1N8O:R	48.57	34.23	50	1.57	91.58	5.93	85.23	22.27	**68.57**	52.54	**63.16**	1.98	**93.07**	9.1	**89.45**	27.1
1NW9:L	42.42	5.42	37.84	0.65	65.67	2.67	58	10.05	**78.79**	6.79	36.11	0.62	31.34	3.34	47	-6.47
1NW9:R	14.71	3.84	20	0.73	91.84	2.71	80.43	12.76	11.76	7.63	**40**	1.46	**97.12**	1.25	**85.12**	14.41
1OC0:L	86.67	22.78	56.52	1.06	52.38	16.27	66.67	15.67	**100**	11.11	46.88	0.88	19.05	7.94	52.78	0
1OC0:R	33.33	29.04	37.5	3.5	97.18	1.47	94.1	8.19	16.67	13.45	25	2.33	**97.46**	0.68	93.57	6.81
1OPH:L	45.16	32.43	50	1.68	92.59	5.32	85.91	20.86	**64.52**	46.13	48.78	1.64	89.06	7.45	85.65	22.88
1OPH:R	0	-5.38	0	0	94.43	-0.19	91.13	3.45	**76.92**	72.08	**55.56**	7.14	**97.77**	2.61	**97.04**	8.91
1OYV:L	30.77	-1.4	21.62	0.58	67.42	-0.41	59.13	4.55	**53.85**	-0.94	**22.22**	0.6	44.94	-0.27	46.96	-1.81
1OYV:R	38.1	27.88	57.14	1.63	94.83	5.05	86.13	24.2	**47.62**	34.48	55.56	1.59	93.1	6.24	86.13	25.07
1PPE:L	**94.44**	1.34	62.96	0.77	9.09	2.19	62.07	-15.36	88.89	16.48	**76.19**	0.93	**54.55**	26.96	**75.86**	11.6
1PPE:R	37.84	23.29	43.75	1.21	90.16	4.71	81.36	21.63	**62.16**	43.33	**54.76**	1.51	89.78	8.62	**85.2**	26.65
1PXV:L	32.26	-3.78	25	0.56	62.5	-1.46	54.05	2.64	**41.94**	26.62	**76.47**	1.7	**95**	10.32	**80.18**	26.66
1PXV:R	33.33	16.19	40	1.12	87.05	4.19	76	16.57	**61.11**	25.68	35.48	1	71.22	6.65	69.14	14.96
1R0R:L	56.25	1.35	32.14	0.72	45.71	0.62	49.02	-0.44	**87.5**	12.99	**36.84**	0.83	31.43	5.94	49.02	1.74
1R0R:R	51.61	37.38	41.03	1.57	90.53	4.77	86.13	19	**58.06**	44.93	**50**	1.92	**92.59**	5.73	**88.69**	21.03
1TMQ:L	23.33	-5.73	20.59	0.47	68.97	-1.97	57.26	4.8	**46.67**	32.99	**87.5**	1.98	**97.7**	11.38	**84.62**	30.34
1TMQ:R	41.86	35.48	60	2.68	97.19	3.57	92.13	18.05	**58.14**	49.84	**64.1**	2.86	96.72	5.02	**93.19**	20.17
1UDI:L	33.33	20.08	90.91	1.67	98.11	11.37	74.7	28.04	**83.33**	11.04	41.67	0.76	33.96	6.25	51.81	-0.22
1UDI:R	56.25	38.71	45	1.56	88.78	6.32	84.21	20.47	**81.25**	62.83	**61.9**	2.15	**91.84**	10.26	**90.35**	26.98
1YVB:L	35.29	1.96	16.67	0.71	67.03	0.37	62.04	3.24	**88.24**	69.72	**75**	3.18	**94.51**	13.02	**93.52**	26.9
1YVB:R	31.43	15.66	28.95	0.94	86.89	2.66	78.84	15.63	**65.71**	38.74	**35.38**	1.15	79.61	6.58	77.59	18.7

Protein	Sens	ScSens	PPV	ScPPV	Spe	ScSpe	Acc	ScAcc	Sens	ScSens	PPV	ScPPV	Spe	ScSpe	Acc	ScAcc
1ZLI:L	68.75	19.42	59.46	0.95	65.12	14.45	66.67	16.84	**81.25**	5.25	45.61	0.73	27.91	3.91	50.67	-5.96
1ZLI:R	5	-4.48	6.9	0.22	93.98	3.46	82.35	17.36	**22.5**	14.04	**26.47**	0.84	93.09	1.55	**86.07**	20.7
2ABZ:L	57.89	3.06	32.35	0.68	46.51	1.35	50	0.24	**89.47**	15.28	**36.96**	0.78	32.56	6.75	50	1.21
2ABZ:R	29.63	18.08	22.86	1.07	90.22	1.77	84.82	12.85	**48.15**	27.69	20.97	0.98	82.25	2.71	79.21	12.33
2B42:L	46.34	27.42	54.29	1.19	88.89	7.81	79.46	26.7	**58.54**	28.27	42.86	0.94	77.78	8.05	73.51	21.76
2B42:R	6.98	1.45	15	0.57	94.67	0.2	84.25	13.24	**20.93**	17.89	**81.82**	3.1	**99.37**	2.41	**90.06**	17.87
2J0T:L	28.57	4.76	20	0.73	77.14	0.95	69.05	7.14	**33.33**	23.02	**53.85**	1.97	**94.29**	4.6	**84.13**	16.09
2J0T:R	51.72	33.25	51.72	1.5	89.06	7.53	82.17	22.41	**86.21**	47.35	40.98	1.19	71.88	10.73	74.52	21.07
2MTA:L	70	38.57	42.42	1.29	77.65	9.08	76.19	19.8	**80**	43.81	42.11	1.28	74.12	10.31	75.24	20.48
2MTA:R	50	38.12	24.56	1.78	90.49	2.36	88.12	10.52	17.86	15.77	**50**	3.62	**98.89**	0.98	**94.17**	9.47
2O3B:L	47.37	27.37	33.33	1.35	84.48	4.48	79.26	14.06	**84.21**	38.28	25.81	1.05	60.34	6.27	63.7	11.64
2O3B:R	50	38.28	53.57	1.96	93.78	5.5	88.28	20.88	**70**	52.01	48.84	1.79	89.47	7.47	87.03	22.48
2O8V:L	80.95	51.32	53.12	1.52	82.76	12.39	82.41	26.3	**85.71**	49.6	46.15	1.32	75.86	11.97	77.78	23.61
2O8V:R	50	32.33	13.16	1.72	83.9	1.58	82.33	4.94	**70**	63.95	**53.85**	7.05	**97.07**	3.12	**95.81**	8.57
2OUL:L	82.14	53.96	74.19	1.88	90.24	18.43	88.18	33.57	**85.71**	52.99	66.67	1.69	85.37	18.09	85.45	31.81
2OUL:R	38.24	20.86	31.71	1.08	86.14	3.51	79.24	15.74	**67.65**	33.33	28.4	0.97	71.29	5.61	70.76	14.28
2PCC:L	22.22	-4.63	13.79	0.45	72.22	-0.93	63.89	4.87	**94.44**	35.19	**26.56**	0.87	47.78	7.04	55.56	9.17
2PCC:R	6.67	-4.25	3.12	0.27	88.85	-0.23	84.64	4.72	**20**	0.89	**5.36**	0.46	80.94	0.05	77.82	4.17
2SIC:L	0	-16.67	0	0	80.65	-2.69	69.44	1.74	**73.33**	57.59	**64.71**	2.76	**93.55**	9.29	**90.74**	22.54
2SIC:R	44.12	33.57	51.72	1.78	94.19	4.74	88	21.48	**70.59**	54.95	**55.81**	1.92	92.12	7.75	**89.45**	25.06
2SNI:L	64.71	20.09	37.93	0.98	62.5	7.12	63.08	11.85	100	15.38	30.91	0.8	20.83	5.45	41.54	-0.59
2SNI:R	60	49.42	62.07	2.38	95.49	6.08	91.61	22.75	**83.33**	61.8	42.37	1.62	86.07	7.6	85.77	22.15
2UUY:L	58.33	14.7	29.17	0.95	60.47	4.1	60	7.55	**83.33**	43.33	**45.45**	1.48	**72.09**	12.09	**74.55**	20.7
2UUY:R	45.16	31.98	48.28	1.62	92.06	5.25	85.45	20.59	**61.29**	43.35	47.5	1.59	89.06	7	85.2	22.25
3SGQ:L	64.29	5.46	30	0.77	43.24	2.07	49.02	0.98	**92.86**	2.66	28.26	0.73	10.81	1.01	33.33	-7.73
3SGQ:R	22.22	0.19	15.38	0.53	78	0.03	69.49	7.76	**81.48**	50.13	**37.93**	1.31	77.22	8.57	**77.84**	20.02
4CPA:L	0	0	0	0	0	0	0	0	**41.67**	-27.56	**18.52**	0.37	**18.52**	-12.25	**25.64**	-24.36
4CPA:R	52.17	39.14	30	1.59	90.14	3.17	87.3	14.27	**56.52**	32.42	17.57	0.93	78.52	2.63	76.87	10.74
7CEI:L	10	-10.61	7.41	0.27	77.48	-1.91	67.18	4.11	**45**	-0.8	**15**	0.54	54.05	-0.14	52.67	0.81
7CEI:R	47.83	1.85	27.5	0.69	54.69	0.66	52.87	2.04	**73.91**	3.8	**27.87**	0.7	31.25	1.36	42.53	-3.31
BOYV:L	40	9.57	11.43	0.8	70.48	0.91	67.83	3.85	100	68.7	**27.78**	1.94	**75.24**	6.54	**77.39**	14.04
BOYV:R	55.17	44.22	53.33	2.19	94.29	5.23	90.15	20.13	**58.62**	45.48	47.22	1.94	92.24	5.38	88.69	19.79
**All**	**40.03**	**16.73**	**35.49**	**1.15**	**78.58**	**3.8**	**74.34**	**12.58**	**64.69**	**31.35**	**44.81**	**1.62**	**73.23**	**6.57**	**75.35**	**13.99**

The legend is the same as in S8 Table.
