# Supplementary material for: Local Geometry and Evolutionary Conservation of Protein Surfaces Reveal the Multiple Recognition Patches in Protein-Protein Interactions
Source: PLoS Comput Biol. 2015 Dec 21;11(12):e1004580. doi: 10.1371/journal.pcbi.1004580 (PMC4686965; doi:10.1371/journal.pcbi.1004580)
Supplement: S14 Table — (PDF) [file pcbi.1004580.s014.pdf]

Other																
	**iJET**								**iJET^2**							
**Protein**	**Sens**	**ScSens**	**PPV**	**ScPPV**	**Spe**	**ScSpe**	**Acc**	**ScAcc**	**Sens**	**ScSens**	**PPV**	**ScPPV**	**Spe**	**ScSpe**	**Acc**	**ScAcc**
1A2K:L	85	67.18	47.22	2.29	89.56	7.38	89.11	20.2	**95**	76.68	**51.35**	2.49	**90.11**	8.43	**90.59**	21.98
1A2K:R	38.46	23.01	26.32	1.22	87.27	2.72	82.11	12.41	**61.54**	41.62	**32.65**	1.52	85	4.92	**82.52**	15.37
1AK4:L	0	-29.93	0	0	67.72	-2.36	62.77	-2.79	100	87.59	**58.82**	5.24	**94.49**	6.9	**94.89**	15.75
1AK4:R	70	48.05	38.89	1.46	84.72	6.67	82.93	19.84	**95**	60.85	33.93	1.27	74.31	8.45	76.83	19.43
1AKJ:L	19.44	-19.15	7.95	0.25	57.81	-3.59	51.75	-2.51	**50**	6.14	**18**	0.57	57.29	1.15	**56.14**	3.84
1AKJ:R	32.35	11.82	14.29	0.81	80.65	1.18	76.27	7.24	20.59	16.05	**41.18**	2.33	**97.07**	1.6	**90.13**	10.77
1ATN:L	6.45	4.9	50	1.82	99.12	0.67	87.98	16.12	**25.81**	10.3	20	0.73	85.9	1.41	78.68	13.11
1ATN:R	0	-8.63	0	0	90.83	-0.54	85.44	4.18	**36.36**	33.94	**88.89**	7.27	**99.71**	2.14	**95.96**	10.01
1AZS:L	59.26	49.11	47.06	2.77	94.16	4.31	91.34	15.03	**70.37**	58.73	**48.72**	2.87	93.51	5.15	**91.64**	16.31
1AZS:R	0	-9.63	0	0	89.82	-0.55	84.99	3.59	0	-4.82	0	0	**94.91**	-0.27	**89.8**	4.66
1B6C:L	7.69	-8.67	3.7	0.22	82.89	-0.74	76.97	4.62	**19.23**	16.5	**55.56**	3.31	**98.68**	1.41	**92.42**	11.01
1B6C:R	66.67	24.61	40	0.89	66.25	8.31	66.36	15.56	37.04	21.15	**58.82**	1.31	**91.25**	7.14	**77.57**	24.16
1BKD:L	54.55	32.26	64.86	1.37	89.34	11.63	80.12	28.63	**65.91**	42.42	**74.36**	1.57	**91.8**	15.3	**84.94**	33.51
1BKD:R	26.42	22.53	77.78	3.21	99.02	2.91	90.71	16.92	**45.28**	36	55.81	2.31	95.37	4.65	89.63	18.63
1BUH:L	65	25.53	43.33	0.95	69.64	9.12	68.42	17.46	100	32.89	39.22	0.86	44.64	11.75	59.21	10.77
1BUH:R	11.11	-1.47	5.41	0.41	87.32	-0.1	82.65	5.07	**38.89**	34.47	**53.85**	4.1	**97.83**	2.25	**94.22**	10.62
1DE4:L	0	-1.49	0	0	98.48	-0.03	96.24	4.08	0	-0.55	0	0	**99.44**	-0.01	**97.18**	4.21
1DE4:R	0	-9.7	0	0	89.5	-0.79	82.75	4.08	**14.29**	11.86	**44.44**	3.08	**98.54**	0.97	**92.18**	8.34
1E96:L	65	48.85	41.94	2.03	89.53	5.68	86.98	17.09	**85**	57.4	32.08	1.56	79.07	6.67	79.69	16.53
1E96:R	38.89	14.58	15.91	0.83	77.3	1.61	73.48	7.63	38.89	30.15	**43.75**	2.28	**94.55**	3.29	**89.07**	13.61
1EER:L	37.74	24.69	37.04	1.65	90.58	3.63	83.82	13.46	**49.06**	40.36	**72.22**	3.22	**97.23**	5.93	**91.06**	18.31
1EER:R	46.34	24.05	51.35	1.24	85.6	7.89	75.9	21.15	24.39	13.55	**55.56**	1.34	**93.6**	4.44	**76.51**	19.78
1EFN:L	100	35.09	43.24	0.92	48.78	13.69	63.16	14.04	100	17.54	34.04	0.72	24.39	6.85	45.61	-2.48
1EFN:R	0	0	0	0	0	0	0	0	**57.14**	45.02	**66.67**	2.86	**95.29**	7.42	**89.9**	19.7
1F51:L	56.25	24.85	47.37	0.99	77.53	8.93	71.9	21.07	**81.25**	35.8	47.27	0.99	67.42	12.87	71.07	20.87
1F51:R	38.89	29.85	45.16	2.33	94.46	3.5	88.63	13.52	**52.78**	43.74	**61.29**	3.17	**96.09**	5.13	**91.55**	16.44
1FAK:L	30.19	14.8	57.14	1.07	90.7	6.08	73.08	25.52	**49.06**	16.09	43.33	0.81	73.64	6.61	66.48	17.69
1FAK:R	23.68	18.51	50	2.38	97.1	2.27	89.08	13.08	**28.95**	22.34	47.83	2.28	96.13	2.74	88.79	13.62
1FC2:L	50	44.44	43.48	2.67	96.7	2.26	94.44	14.45	0	-2.17	0	0	**97.72**	-0.11	93	10.72
1FC2:R	68.75	15.42	34.38	0.77	52.27	5.61	56.67	7.04	**93.75**	2.08	27.27	0.61	9.09	0.76	31.67	-13.7
1FCC:L	64.71	7.56	34.38	0.85	46.15	3.3	51.79	3.15	64.71	3.99	32.35	0.8	41.03	1.74	48.21	0.26
1FCC:R	16.67	12.08	15.79	1.92	95.96	0.55	92.51	4.57	0	-2.17	0	0	**97.73**	-0.1	**93.48**	3.51
1FFW:L	87.5	42.57	45.16	1.27	67.92	12.85	72.46	21	100	36.23	36.36	1.02	47.17	10.94	59.42	13.4
1FFW:R	57.14	23.55	18.6	0.93	69.3	2.89	67.97	8.13	**85.71**	34.93	18.46	0.92	53.51	4.29	57.03	7.5
1FQJ:L	60.71	35.15	50	1.34	83.81	9.37	78.95	22.76	**92.86**	55.9	**50.98**	1.37	77.27	14.23	**80.43**	27.13
1FQJ:R	41.67	34.7	45.45	2.73	95.89	2.85	91.77	13.08	**62.5**	53.64	**53.57**	3.21	95.55	4.41	**93.04**	15.61
1GCQ:L	52.63	1.89	29.41	0.68	50	0.75	50.75	0.85	**89.47**	11.86	**32.69**	0.76	27.08	4.7	44.78	-1.46
1GCQ:R	0	-9	0	0	90.31	-0.69	83.89	22.17	**33.33**	29.07	**55.56**	1.56	**97.96**	2.22	**93.36**	30.3
1GHQ:L	0	-26.15	0	0	71.67	-2.18	66.15	-2.21	**50**	5.38	**8.62**	0.75	55.83	0.45	55.38	1.24
1GHQ:R	0	-14.63	0	0	84.7	-0.68	80.95	3.18	**76.92**	64	**26.32**	2.45	**90.04**	2.96	**89.46**	10.35
1GLA:L	65	41.67	37.14	1.62	83.08	6.41	80.67	16.26	**85**	51.67	34	1.48	74.62	7.95	76	17
1GLA:R	0	-8.05	0	0	91.65	-0.3	88.33	4.72	**16.67**	5.6	**5.45**	0.55	89.14	0.21	86.52	5.33
1GP2:L	35.9	27.5	41.18	1.86	94.54	2.93	88.89	15.72	**64.1**	54.72	**65.79**	2.97	**96.45**	5.83	**93.33**	20.72
1GP2:R	71.43	62.4	53.57	4.69	95.5	4.53	93.87	12.25	**80.95**	71.27	**56.67**	4.97	95.5	5.18	**94.52**	13.4
1GPW:L	51.85	37.35	48.28	1.63	91.33	5.83	86	21.57	**81.48**	61.48	**55**	1.85	89.6	9.6	**88.5**	26.3
1GPW:R	0	-17.79	0	0	79.26	-2.95	67.98	4.63	0	-3.95	0	0	**95.39**	-0.66	**81.82**	12.73
1GRN:L	48.15	31.75	41.94	1.6	88.89	5.29	83.07	17.09	**70.37**	49.21	**47.5**	1.81	87.04	8.2	**84.66**	20.94
1GRN:R	59.26	40.31	44.44	1.66	87.73	6.68	83.68	19.23	**70.37**	50.9	**51.35**	1.92	**88.96**	8.43	**86.32**	22.11
1H1V:L	2.94	-0.35	4.17	0.2	96.69	-0.02	92.32	14.63	2.94	-0.49	4	0.2	96.55	-0.02	92.18	14.57
1H1V:R	3.33	-5.29	3.12	0.18	90.91	-0.47	83.83	6.89	**26.67**	20.2	**33.33**	1.91	**95.31**	1.78	**89.76**	11.41
1H9D:L	33.33	12.77	37.93	0.79	83.33	3.9	71.63	20.35	**69.7**	17.92	31.51	0.66	53.7	5.48	57.45	7.52
1H9D:R	10.34	-16.85	9.68	0.23	67.06	-5.75	52.63	-0.72	**31.03**	17.88	**60**	1.41	**92.94**	6.1	**77.19**	21.78
1HCF:L	46.43	3.41	35.14	0.78	58.62	1.64	54.65	3.98	**92.86**	6.81	35.14	0.78	17.24	3.29	41.86	-4.65
1HCF:R	0	0	0	0	0	0	0	0	0	-3.7	0	0	**95.81**	-0.48	**84.72**	4.96
1HE1:L	65.22	40.36	33.33	1.32	81.01	5.87	79.01	16.57	52.17	42.88	**70.59**	2.79	**96.88**	6.16	**91.26**	21.13
1HE1:R	37.5	11.55	35.29	0.87	77.78	3.73	67.94	13.37	**75**	38.43	**48.98**	1.21	75.49	12.06	**75.37**	22.82
1HE8:L	65	41.51	33.33	1.4	82.19	5.69	80.12	16.24	**95**	64.88	**38**	1.6	78.77	8.89	**80.72**	20.31
1HE8:R	0	-4.77	0	0	95.13	-0.1	93.21	2.76	0	-0.95	0	0	**99.03**	-0.02	**97.02**	3.17
1I2M:L	14.89	9.41	31.82	1.01	95.76	1.25	86.28	19.85	**34.04**	21.82	**32.65**	1.04	90.68	2.9	84.04	20.09
1I2M:R	35.29	18.46	35.29	0.86	86.9	3.74	78.22	22.22	**47.06**	26.27	**38.1**	0.93	84.52	5.32	78.22	22.94
1I4D:L	65.22	41.46	34.88	1.33	82.28	6.04	80.11	17.59	**78.26**	55.31	**42.86**	1.64	**85**	7.95	**84.15**	21.24
1I4D:R	0	-0.5	0	0	99.47	-0.03	93.03	6.03	7.69	1.72	8.33	0.66	94.15	0.12	88.56	5.64
1IB1:L	29.03	6.14	23.68	0.74	78.52	1.41	69.28	9.5	**58.06**	44.21	**78.26**	2.45	**96.3**	10.15	**89.16**	26.11
1IB1:R	56.52	47.61	63.41	3.16	96.38	5.29	92.39	17.81	**80.43**	64.35	50	2.49	91.06	7.15	90	19.71
1IBR:L	12.96	6.14	23.33	1	94.04	0.86	84.09	11.1	5.56	4.42	**60**	2.57	**99.48**	0.62	**87.95**	11.94
1IBR:R	37.78	18.21	47.22	0.9	86.33	5.9	74.46	25.87	**53.33**	26.16	**48**	0.92	81.29	8.47	74.46	25.52
1IRA:L	12.24	-7.07	21.43	0.35	77.08	-3.61	55.17	11.61	**40.82**	21.51	**71.43**	1.18	**91.67**	10.98	**74.48**	30.92
1IRA:R	5.56	-1.86	13.04	0.43	92.19	-0.39	77.1	10.21	**9.26**	-9.45	8.62	0.29	79.3	-1.99	67.1	4.69
1J2J:L	92.31	10.26	37.5	0.98	23.08	5.13	46.15	3.7	84.62	17.95	**42.31**	1.11	**42.31**	8.97	**56.41**	10.33
1J2J:R	83.33	59.24	37.5	1.67	83.11	7.2	83.13	18.89	**94.44**	67.34	**37.78**	1.68	81.08	8.19	82.53	19.94
1JK9:L	48.57	27	51.52	1.27	86.44	8.01	77.78	22.49	**80**	44.05	50.91	1.25	77.12	13.07	77.78	25.16
1JK9:R	53.85	38.78	42.42	2.25	90.16	5.22	85.84	14.08	**84.62**	64.25	**48.89**	2.59	88.21	8.57	**87.78**	19.31
1JMO:L	16.98	1.63	24.32	0.61	85.11	0.46	70.12	12.88	**60.38**	33.04	**42.11**	1.06	80.44	7.78	**76.62**	21.88
1JMO:R	3.57	-0.57	6.25	0.47	95.81	-0.04	89.12	5.49	**50**	45.34	**77.78**	5.83	**98.88**	3.55	**95.34**	12.09
1JWH:L	12.5	0	7.32	0.48	87.5	0	82.01	5.9	**41.67**	37.4	**71.43**	4.7	**98.68**	2.95	**94.51**	12.67
1JWH:R	18.75	11.65	11.54	1.49	93.43	0.53	90.16	3.9	**50**	37.98	**18.18**	2.35	89.71	1.74	87.98	5.87
1JZD:L	45.45	19.59	50	1.08	81.93	7.79	71.55	19.85	**48.48**	19.17	47.06	1.01	78.31	7.62	69.83	18.37
1JZD:R	65.62	51.01	33.33	2.27	89.47	4.09	87.7	12.71	**78.12**	57.48	28.09	1.91	83.96	4.61	83.53	12.79
1K5D:L	55.32	45.7	78.79	2.65	97.64	7.26	91.84	25.48	**63.83**	51.58	71.43	2.4	95.95	8.19	91.55	26.25
1K5D:R	43.24	34.37	53.33	2.52	95.35	4.22	89.64	15.91	**51.35**	40.7	52.78	2.5	94.35	5	89.64	16.93
1K74:L	36.67	20.82	24.44	1.12	86.61	2.46	81.34	12.03	**63.33**	48.19	**44.19**	2.03	**90.55**	5.69	**87.68**	17.97
1K74:R	44.83	30.79	39.39	1.7	90.29	4.33	84.68	15.41	**72.41**	54.54	**50**	2.16	89.81	7.68	**87.66**	20.44
1KAC:L	33.33	13.5	33.33	1.03	83.51	3.34	73.55	12.96	**45.83**	23.52	**40.74**	1.26	83.51	5.82	**76.03**	16.31
1KAC:R	52.38	35.08	34.38	1.67	87.2	4.49	83.24	14.01	**80.95**	60.41	**44.74**	2.17	87.2	7.74	**86.49**	19.16

Protein	Sens	ScSens	PPV	ScPPV	Spe	ScSpe	Acc	ScAcc	Sens	ScSens	PPV	ScPPV	Spe	ScSpe	Acc	ScAcc
1KLU:L	0	-9.24	0	0	90	-0.76	83.19	4.46	**33.33**	29.55	**66.67**	4.52	**98.64**	2.42	**93.7**	11.12
1KLU:R	5.26	-7.47	2.13	0.21	86.86	-0.41	82.66	2.88	**42.11**	32.35	**22.22**	2.21	**92**	1.76	**89.43**	7.28
1KTZ:L	75	41.67	34.29	1.26	74.16	7.49	74.29	16.66	**93.75**	42.32	27.78	1.02	56.18	7.61	61.9	12.56
1KTZ:R	40	8.75	11.43	0.67	69.61	0.86	66.96	4.57	**80**	68.39	**61.54**	3.63	**95.1**	6.71	**93.75**	18.37
1KXP:L	0	-4.19	0	0	95.24	-0.57	83.89	8.61	**5.56**	4.23	**50**	2.23	**99.25**	0.57	**88.08**	11.22
1KXP:R	2.33	-6.57	3.03	0.12	90.24	-0.86	80.05	9.87	**37.21**	32.9	**100**	3.93	**100**	4.31	**92.72**	20.28
1LFD:L	66.67	27.59	29.41	1.1	66.67	5.75	66.67	11.6	**100**	37.93	27.78	1.04	45.83	7.9	55.17	10.78
1LFD:R	68.42	46.73	36.11	1.65	84.35	6.04	82.53	16.58	**94.74**	65.82	**37.5**	1.72	79.59	8.51	81.33	19.45
1ML0:L	75	20.07	61.54	0.81	61.54	16.47	67.61	15.02	56.25	23.86	**78.26**	1.03	**87.18**	19.57	**73.24**	32.46
1ML0:R	0	0	0	0	0	0	0	0	**14.71**	12.55	**31.25**	3.16	**98.45**	0.6	**94.61**	6.22
1MQ8:L	61.9	37.63	30.95	1.46	80.92	5.2	78.61	13.8	**66.67**	49.9	**48.28**	2.28	**90.13**	6.89	**87.28**	18.15
1MQ8:R	0	-14.59	0	0	83.73	-1.67	75.14	1.96	0	-7.57	0	0	**91.57**	-0.87	**82.16**	4.39
1N2C:L	42.37	28.11	30.49	0.98	88.95	3.21	84.17	20.75	**61.02**	50.06	**57.14**	1.83	**94.77**	5.72	**91.3**	26.64
1N2C:R	30.36	29.3	80.95	8.57	**99.79**	0.85	**97.83**	8.13	**71.43**	68.7	74.07	7.84	99.27	2	**98.48**	10.14
1OFU:L	0	-10.46	0	0	88.81	-0.73	83.01	4.68	**45**	39.12	**50**	3.52	**96.85**	2.74	**93.46**	11.86
1OFU:R	85	69.03	44.74	2.64	90.37	6.33	89.92	17.42	**90**	76.13	**54.55**	3.22	**93.12**	6.98	**92.86**	18.97
1PVH:L	41.67	30.35	27.78	2.18	91.16	2.48	87.42	8.62	16.67	5.97	11.76	0.92	89.8	0.49	84.28	5
1PVH:R	0	-5.29	0	0	94.15	-0.56	85.1	5.17	**35**	30.67	**77.78**	4.71	**98.94**	3.26	**92.79**	12.21
1QA9:L	0	-5.85	0	0	93.46	-0.69	83.63	17.36	**44.44**	23.39	**22.22**	0.7	81.7	2.75	77.78	17.11
1QA9:R	0	-12.29	0	0	86.08	-1.63	75.98	12.27	**19.05**	2.29	**13.33**	0.42	83.54	0.3	75.98	13.89
1R6Q:L	28.57	-8.51	18.18	0.5	60.29	-2.63	52.81	-0.76	**95.24**	18.83	**29.41**	0.81	29.41	5.82	44.94	2.23
1R6Q:R	42.86	17.51	33.33	0.96	78.95	4.3	71.83	14.22	**57.14**	25.45	**35.56**	1.03	74.56	6.25	71.13	15.48
1R8S:L	54.76	40.4	82.14	2.01	96.73	11.09	87.69	31.12	**88.1**	58.35	63.79	1.56	86.27	16.02	86.67	32.93
1R8S:R	67.65	47.09	62.16	1.57	90.41	10.97	86.11	29.95	61.76	42.88	61.76	1.56	**91.1**	9.98	85.56	29.04
1RLB:L	27.78	8.92	15.15	0.79	82.17	1.02	76.57	7.36	**72.22**	60.22	**61.9**	3.23	**94.9**	6.9	**92.57**	19.12
1RLB:R	30.43	9.38	7.29	0.68	79.45	0.5	76.97	4.19	**78.26**	33.52	**8.82**	0.83	57.04	1.78	58.11	3.97
1RV6:L	38.1	6.57	27.59	0.78	70.42	1.94	63.04	7.72	**57.14**	43.01	**92.31**	2.59	**98.59**	12.72	**89.13**	28.8
1RV6:R	40	23	29.41	1.38	86.29	3.29	80.5	11.6	**92**	55.5	**31.51**	1.47	71.43	7.93	74	16.27
1S1Q:L	76.47	35.04	44.83	1.29	69.81	11.24	71.43	18.8	**100**	27.14	33.33	0.96	35.85	8.71	51.43	8.43
1S1Q:R	47.37	22.89	25.71	1.14	79.03	3.51	74.83	10.85	**89.47**	41.92	25	1.11	58.87	6.42	62.94	11.6
1SBB:L	5.88	0.86	8.33	0.59	95.05	0.07	88.7	6.36	**35.29**	8.93	**9.52**	0.68	74.32	0.68	71.55	4.55
1SBB:R	0	-12.66	0	0	86.36	-0.98	80.17	2.16	**5.88**	0.42	**7.69**	0.62	**94.57**	0.03	**88.24**	4.83
1SYX:L	56.25	7.86	30	0.84	54.35	2.73	54.84	4.37	**87.5**	21.37	**34.15**	0.96	41.3	7.43	53.23	7.89
1SYX:R	10	-9.85	7.69	0.27	78.38	-1.77	67.94	4.77	**45**	2.25	**16.07**	0.57	57.66	0.41	55.73	2.56
1T6B:L	42.31	22.97	31.43	1	84.52	3.85	78.45	17	**84.62**	54.78	**40.74**	1.3	79.35	9.19	**80.11**	22.58
1T6B:R	0	-2.41	0	0	97.48	-0.11	93.23	4.32	**31.03**	28.33	**50**	5.48	**98.58**	1.29	**95.64**	6.97
1US7:L	47.06	25.35	28.57	2.15	82.14	3.85	77.52	6.74	47.06	38.53	**72.73**	5.48	**97.32**	5.85	**90.7**	10.24
1US7:R	57.14	38.79	31.58	1.52	86.02	4.38	83.09	14.61	**90.48**	60.52	30.65	1.47	76.88	6.83	78.26	16.55
1WDW:L	69.05	52.04	70.73	1.85	93.97	10.98	89.63	31.83	**85.71**	56.67	51.43	1.35	82.91	11.96	83.4	28.45
1WDW:R	6.98	1.93	7.69	0.48	95.07	0.11	90.17	9.64	**76.74**	69.63	**60**	3.74	**96.99**	4.1	**95.86**	16.74
1WQ1:L	64.86	43.81	66.67	1.55	91.04	12.1	85.38	31.34	**72.97**	47.24	61.36	1.43	87.31	13.04	84.21	30.82
1WQ1:R	52.17	41.99	72.73	2.55	96.76	6.95	90.43	23.37	**69.57**	49.81	50	1.75	88.49	8.24	85.8	22.84
1XD3:L	78.26	35.4	60	1.3	74.47	17.33	75.71	25.14	**95.65**	32.8	50	1.09	53.19	16.05	67.14	18.17
1XD3:R	6.25	1.88	22.22	0.8	95.98	0.35	82.04	11.8	**59.38**	42.87	**55.88**	2.01	91.38	7.88	**86.41**	21.55
1XQS:L	3.45	-22.46	2	0.07	70.12	-3.97	60.1	0.57	**20.69**	9.29	**27.27**	0.9	**90.24**	1.64	**79.79**	14.51
1XQS:R	27.03	19.69	52.63	1.76	95.95	3.28	86.1	18.9	**48.65**	31.27	40	1.34	87.84	5.21	82.24	19.08
1XU1:L	84.21	25.24	69.57	1.14	65	23.97	74.36	22.33	**100**	15.38	57.58	0.94	30	14.62	64.1	6.29
1XU1:R	37.04	29.12	31.25	2.03	94.16	2.09	90.35	11.25	**77.78**	44.11	15.44	1	69.5	3.16	70.05	8.75
1Y64:L	12.24	5.02	23.08	0.79	93.57	0.79	82.5	14.83	10.2	7.98	**62.5**	2.13	**99.04**	1.26	**86.94**	17.2
1Y64:R	0	-6.33	0	0	93.09	-0.59	85.16	3.11	0	-0.49	0	0	**99.47**	-0.05	**91**	4.66
1Z0K:L	93.75	24.18	46.88	1.35	43.33	12.9	60.87	16.82	**100**	2.17	35.56	1.02	3.33	1.16	36.96	1.51
1Z0K:R	53.85	33.96	40	1.28	86	5.89	81.25	20	**65.38**	46.63	**51.52**	1.64	**89.33**	8.08	**85.8**	24.12
1Z5Y:L	71.43	49.11	55.56	1.85	88	10.31	85.12	24.05	**85.71**	56.79	51.43	1.71	83	11.93	83.47	25.04
1Z5Y:R	62.5	40.35	45.45	1.33	85.6	7.75	81.88	23.13	**66.67**	39.82	40	1.17	80.8	7.65	78.52	21.25
1ZHH:L	0	-14.76	0	0	82.39	-2.85	69.05	5.59	**14.71**	7.09	**31.25**	1.01	**93.75**	1.37	**80.95**	14.77
1ZHH:R	0	-11.24	0	0	87.9	-0.86	81.66	4.11	**16.67**	11.05	**21.05**	1.46	**95.22**	0.84	**89.64**	8.1
1ZHI:L	45	22.19	34.62	1.38	81.91	4.72	75.44	11.84	**85**	51.67	**44.74**	1.79	77.66	10.99	**78.95**	20.61
1ZHI:R	35	15.79	17.95	1.02	82.51	1.73	77.83	7.85	15	7.12	**18.75**	1.07	**92.9**	0.78	**85.22**	7.88
1ZM4:L	8.7	-13.36	4.44	0.21	76.24	-1.7	68.63	2.7	**26.09**	10.89	**19.35**	0.9	**86.19**	1.38	**79.41**	9.57
1ZM4:R	0	-1.94	0	0	97.99	-0.07	94.42	4.42	**16.67**	14.85	**33.33**	3.98	**98.74**	0.56	**95.76**	5.65
2A5T:L	29.03	18.96	32.14	1.37	92.31	2.38	85.25	14.08	**67.74**	41.84	29.17	1.24	79.35	5.25	78.06	15.28
2A5T:R	46.15	33.61	33.33	1.67	90.8	3.35	86.76	14.29	**80.77**	53.24	26.58	1.33	77.78	5.3	78.05	14.56
2A9K:L	9.52	-3.91	7.41	0.38	86.11	-0.46	78.11	5.9	**47.62**	41.65	**83.33**	4.25	**98.89**	4.86	**93.53**	16.79
2A9K:R	51.72	29.7	40.54	1.22	84.17	6.2	78.57	19.25	**55.17**	28.39	35.56	1.07	79.14	5.92	75	17.26
2AJF:L	4	-14.03	3.03	0.12	79.75	-2.22	69.4	3.58	**32**	23.26	**50**	1.98	**94.94**	3.68	**86.34**	15.92
2AJF:R	7.69	1.16	5.13	0.54	93.52	0.05	89.78	4.56	**42.31**	34.43	**23.4**	2.47	**93.7**	1.57	**91.46**	7.32
2AYO:L	75.76	34.09	**83.33**	1.26	87.18	28.85	81.94	34.61	**96.97**	26.14	62.75	0.95	51.28	22.12	72.22	15.56
2AYO:R	30.77	21.27	50	1.91	94.39	3.88	84.57	15.23	**42.31**	25.39	38.6	1.48	87.72	4.63	80.71	14.92
2B4J:L	71.43	32.87	31.25	1.14	68.12	6.67	68.67	13.51	71.43	53.36	**66.67**	2.43	**92.75**	10.83	**89.16**	24.76
2B4J:R	33.33	22.3	18.75	1.26	90.44	1.48	86.9	9.52	27.78	20.88	**25**	1.68	**94.49**	1.38	**90.34**	10.07
2BTF:L	58.06	29.29	45	1.09	79.63	8.41	74.82	21.14	**83.87**	39.99	42.62	1.03	67.59	11.48	71.22	20.16
2BTF:R	10.34	2.26	10	0.61	92.11	0.19	85.71	7.46	**44.83**	37.55	**48.15**	2.96	**95.91**	3.18	**91.91**	13.11

Protein	Sens	ScSens	PPV	ScPPV	Spe	ScSpe	Acc	ScAcc	Sens	ScSens	PPV	ScPPV	Spe	ScSpe	Acc	ScAcc
2C0L:L	5.26	-19.13	3.33	0.12	72.12	-3.49	61.79	0.08	**10.53**	-5.73	**10**	0.37	**82.69**	-1.05	**71.54**	6.12
2C0L:R	31.58	21.65	40	1.47	93.18	3.12	85.43	17.11	**52.63**	38.06	**45.45**	1.67	90.91	5.48	**86.09**	19.9
2CFH:L	9.68	-7.81	12	0.35	80.36	-2.16	65.03	4.92	9.68	2.68	**30**	0.87	**93.75**	0.74	**75.52**	12.14
2CFH:R	41.94	18.04	34.21	1.06	80.47	4.37	72.96	13.71	**74.19**	35.2	**37.1**	1.15	69.53	8.52	70.44	16.54
2FJU:L	57.14	33.94	19.05	1.25	79.64	2.85	77.9	9.26	50	42.35	**50**	3.29	**95.86**	3.51	**92.35**	12.89
2FJU:R	0	-2.26	0	0	97.68	-0.06	95.06	3.55	**10.53**	9.4	**25**	3.83	**99.13**	0.26	**96.75**	4.26
2G77:L	58.62	34.98	43.59	1.26	83.82	7.46	79.39	21.23	**79.31**	57.49	**63.89**	1.85	**90.44**	12.26	**88.48**	29.76
2G77:R	41.03	29.24	43.24	1.75	92.36	4.15	85.99	16.64	**76.92**	62.02	**62.5**	2.53	**93.64**	8.55	**91.61**	23.85
2H7V:L	62.5	38.19	34.09	1.39	81.53	5.84	79.01	15.9	**87.5**	65.1	**51.22**	2.09	**87.42**	9.83	**87.43**	23.35
2H7V:R	0	-11.11	0	0	88.1	-0.79	82.22	2.19	**33.33**	28.52	**46.15**	4.05	**97.22**	2.04	**92.96**	8.07
2HLE:L	64	45.56	61.54	1.82	91.38	9.82	86.52	26.29	36	28.91	**90**	2.66	**99.14**	6.23	**87.94**	24.03
2HLE:R	10.81	-9.19	10.81	0.31	77.7	-2.3	64.32	5.27	**18.92**	-6.49	**14.89**	0.43	72.97	-1.62	62.16	4.74
2HQS:L	58.62	31.77	58.62	1.31	84.81	11.66	77.78	25.28	**75.86**	42.53	**61.11**	1.37	82.28	15.61	**80.56**	28.76
2HQS:R	11.9	0.82	11.36	0.52	89.01	0.1	80.86	9.05	**26.19**	18.63	**36.67**	1.67	**94.65**	2.2	**87.41**	13.63
2HRK:L	72.73	43.6	53.33	1.62	82.72	11.84	80.58	23.42	**86.36**	23.26	29.23	0.89	43.21	6.32	52.43	6.93
2HRK:R	71.43	50.32	39.47	1.84	85.53	6.65	83.89	17.38	52.38	44.6	**78.57**	3.67	**98.11**	5.89	**92.78**	18.65
2I9B:L	7.69	-6.94	11.11	0.32	83.51	-1.86	67.48	6.97	**65.38**	49.94	**89.47**	2.55	**97.94**	13.39	**91.06**	30.78
2I9B:R	18	6.81	30	0.9	90.37	1.56	76.87	13.93	16	3.31	23.53	0.71	88.07	0.76	74.63	12.19
2IDO:L	56.52	11.07	43.33	1.04	60.47	5.92	59.09	8.35	**91.3**	-1.12	34.43	0.82	6.98	-0.6	36.36	-6.69
2IDO:R	55.56	33.72	39.47	1.3	84.35	6.19	79.89	18.81	**74.07**	40.74	34.48	1.14	74.15	7.48	74.14	17.58
2J7P:L	28.95	10.8	23.4	0.76	83.71	1.86	75.68	13.35	**42.11**	11.99	20.51	0.67	71.95	2.06	67.57	9.87
2J7P:R	31.82	18.89	36.84	1.16	90.4	3.33	81.63	18.2	**36.36**	18	29.63	0.93	84.8	3.17	77.55	16.09
2NZ8:L	64.29	39.98	40.91	1.34	83.01	7.32	80.11	20.06	**89.29**	66.88	**60.98**	2	**89.68**	12.08	**89.62**	28.82
2NZ8:R	37.78	27.61	54.84	2.04	94.62	4.78	86.23	17.86	28.89	24.3	**92.86**	3.45	**99.62**	4.21	**89.18**	18.24
2OOB:L	**100**	58.57	48.28	1.66	73.21	14.64	78.57	25	**100**	41.43	34.15	1.17	51.79	10.36	61.43	15
2OOB:R	**100**	24.39	29.03	1	31.25	6.86	46.34	7.08	**100**	17.07	26.47	0.91	21.88	4.8	39.02	2.83
2OOR:L	69.7	50.84	69.7	1.8	92.96	11.81	88.57	31.61	**75.76**	55.76	**71.43**	1.84	92.96	12.96	**89.71**	33.01
2OOR:R	9.38	4.16	7.89	0.81	94.98	0.19	91.22	5.15	**43.75**	32.36	**16.87**	1.73	90.1	1.49	88.07	6.96
2OT3:L	72.73	49.09	61.54	1.51	88.64	12.27	85.45	30.57	**78.79**	49.09	53.06	1.3	82.58	12.27	81.82	28.06
2OT3:R	58.82	44.13	55.56	2.22	92.42	7.11	87.76	20.1	**82.35**	63.17	**59.57**	2.38	91	10.18	**89.8**	24.39
2OZA:L	0	0	0	0	0	0	0	0	**2.38**	0.25	**16.67**	0.69	**97.92**	0.04	**83.69**	8.79
2OZA:R	11.39	0.15	24.32	0.53	88.8	0.05	70.21	16.85	**21.52**	11.18	**50**	1.09	**93.2**	3.53	**75.99**	22.55
2VDB:L	85.71	28.57	56.25	1.12	60	17.14	69.64	19.64	47.62	22.62	**71.43**	1.43	**88.57**	13.57	**73.21**	23.21
2VDB:R	0	-1.37	0	0	98.56	-0.07	93.99	4.35	**18.52**	15.25	**26.32**	2.85	97.48	0.74	93.81	5.72
2Z0E:L	39.29	9.11	31.43	0.83	72.73	2.9	64.66	9.83	**71.43**	25.74	**37.74**	1	62.5	8.19	64.66	13.61
2Z0E:R	43.48	35.64	80	2.57	98.17	6.01	90.28	24.33	**65.22**	51.74	69.77	2.24	95.24	8.72	**90.91**	27.09
3BP8:L	76.19	32.95	50	1.05	69.81	13.05	71.62	21.31	**90.48**	42.48	**52.78**	1.11	68.52	16.52	**74.67**	24.58
3BP8:R	0	-4.96	0	0	94.88	-0.16	91.82	3.46	**4.17**	-4.08	**1.59**	0.21	91.62	-0.13	88.87	3.31
3CPH:L	86.36	62.58	48.72	2.13	85.92	9.7	85.98	21.77	**95.45**	73.3	**56.76**	2.48	**88.97**	11.12	**89.82**	24.74
3CPH:R	69.57	59.95	38.1	3.59	93.72	3.33	92.45	10.62	**86.96**	78.49	**54.05**	5.1	**95.89**	4.36	**95.42**	12.69
3D5S:L	68.18	10.8	42.86	0.92	48.72	6.09	55.74	6.21	**90.91**	8.94	40	0.85	23.08	5.04	47.54	-0.42
3D5S:R	13.64	2.41	9.09	0.51	88.97	0.2	83.33	8.43	**50**	34.01	**23.4**	1.31	86.76	2.75	**84.01**	12.17
**All**	**37.17**	**17.58**	**29.53**	**1.1**	**82.11**	**3.72**	**77.3**	**12.66**	**55.75**	**32.37**	**42.24**	**1.83**	**82.22**	**5.6**	**80.43**	**15.01**

The legend is the same as in S8 Table.
