# Supplementary material for: Local Geometry and Evolutionary Conservation of Protein Surfaces Reveal the Multiple Recognition Patches in Protein-Protein Interactions
Source: PLoS Comput Biol. 2015 Dec 21;11(12):e1004580. doi: 10.1371/journal.pcbi.1004580 (PMC4686965; doi:10.1371/journal.pcbi.1004580)
Supplement: S15 Table — (PDF) [file pcbi.1004580.s015.pdf]

Huang dataset

	PPV	Homo	Hetero	Transients	All
**seed**	100	11 (27%)	5 (21%)	1 (5%)	16 (19%)
	≥80	22 (54%)	11 (45%)	9 (47%)	38 (46%)
	≥60	32 (78%)	15 (63%)	13 (68%)	57 (69%)
**seed+ extension**	100	4 (10%)	1 (4%)	0 (-)	5 (6%)
	≥80	20 (49%)	10 (42%)	5 (26%)	35 (42%)
	≥60	32 (78%)	16 (67%)	13 (68%)	58 (70%)
**seed+ extension+ outer layer**	100	2 (5%)	2 (8%)	0 (-)	3 (4%)
	≥80	19 (46%)	10 (42%)	5 (26%)	34 (41%)
	≥60	31 (76%)	16 (67%)	16 (84%)	60 (72%)

PPDBv4

	PPV	EI	A	AB	O	All
**seed**	100	21 (20%)	3 (12%)	4 (17%)	33 (17%)	61 (17%)
	≥80	52 (50%)	5 (19%)	6 (25%)	70 (35%)	133 (38%)
	≥60	68 (65%)	8 (31%)	15 (63%)	107 (54%)	198 (56%)
**seed+ extension**	100	2 (2%)	0 (-)	2 (8%)	2 (1%)	6 (2%)
	≥80	18 (17%)	1 (4%)	2 (8%)	25 (13%)	46 (13%)
	≥60	45 (43%)	4 (15%)	7 (29%)	83 (42%)	139 (39%)
**seed+ extension+ outer layer**	100	1 (1%)	0 (-)	2 (8%)	3 (2%)	6 (2%)
	≥80	7 (7%)	0 (-)	2 (8%)	14 (7%)	23 (7%)
	≥60	35 (34%)	4 (15%)	6 (25%)	59 (30%)	104 (30%)

The numbers and percentages of proteins from the Huang dataset and PPDBv4 for which iJET^2 consensus predictions (8 iterations over 10) have positive predictive values of 100, at least 80 or at least 60 were computed for each step of JET^2 clustering procedure. The different structural or functional classes of the two datasets were considered. Homo: Homodimers; Hetero: Heterodimers; Transients: Transient proteins; All: all proteins from the Huang dataset. EI: enzyme-inhibitor; A: antibody-antigen; AB: bound antibody-antigen; O: proteins with other function; All: all proteins from PPDBv4.
