# Supplementary material for: Local Geometry and Evolutionary Conservation of Protein Surfaces Reveal the Multiple Recognition Patches in Protein-Protein Interactions
Source: PLoS Comput Biol. 2015 Dec 21;11(12):e1004580. doi: 10.1371/journal.pcbi.1004580 (PMC4686965; doi:10.1371/journal.pcbi.1004580)
Supplement: S16 Table — (PDF) [file pcbi.1004580.s016.pdf]

Huang dataset

	**Homo**	**Hetero**	**Transients**	**All**
**1**	3 (7%)	4 (13%)	5 (26%)	12 (15%)
**2**	11 (25%)	9 (38%)	4 (21%)	24 (30%)
$\geq$ **3**	30 (68%)	11 (46%)	10 (53%)	45 (56%)

PPDBv4

	**A**	**AB**	**EI**	**O**	**All**
**1**	22 (84%)	19 (79%)	78 (75%)	107 (54%)	226 (64%)
**2**	2 (8%)	5 (21%)	21 (20%)	63 (32%)	91 (26%)
$\geq$ **3**	2 (8%)	0 (-)	5 (5%)	28 (14%)	35 (10%)

The numbers and percentages of proteins from the Huang dataset and PPDBv4 which experimental interface contains 1, 2 or more recognition patches are reported, considering the different structural or functional classes of the two datasets.
