# Supplementary material for: Local Geometry and Evolutionary Conservation of Protein Surfaces Reveal the Multiple Recognition Patches in Protein-Protein Interactions
Source: PLoS Comput Biol. 2015 Dec 21;11(12):e1004580. doi: 10.1371/journal.pcbi.1004580 (PMC4686965; doi:10.1371/journal.pcbi.1004580)
Supplement: S17 Table — (PDF) [file pcbi.1004580.s017.pdf]

PDB code	Protein	Ligand	Structural data
**PPDBv4**			
1NCA:L	neuraminidase	sialic acid	4DGR wt. inhibitor
1QFW:L	gonadotropin	-	-
2QFW:L	gonadotropin	-	-
1KKL:R	HPR kinase	ATP	1JB1 (homolog) wt. phosphate
1BGX:L	DNA polymerase	DNA	DNA site detected
1I2M:R	Ran GTPase	GTP	1QG4 wt. GTP
1K5D:L	Ran GAP	-	active site annotated in UNIPROT
1N2C:R	Nitrogenase Mo-Fe protein	CFM/CLF/HCA	3MIN wt. ligand
1N2C:L	Nitrogenase Fe protein	ATP	1XCP wt. ATP
1DE4:R	hemochromatosis protein	-	-
2HMI:L	HIV1 reverse transcriptase	-	-
1BKD:L	Son of sevenless	-	-
1FFW:R	Chemotaxis protein CheY	-	-
1GL1:L	Protease inhibitor LCMI II	-	-
1GLA:R	Glycerol Kinase	ATP	1GLL wt. ATP analog
1GPW:R	HISF protein	PRFAR, glutamine	4EWN wt. product analog
1JK9:R	CCS metallochaperone	copper ions	-
1OFU:R	sulA	-	-
1T6B:R	anthrax PA	-	-
1US7:R	Heat shock protein 82 N-ter domain	ATP	2FXS wt. inhibitor
1WDW:R	Tryptophan synthase beta chain 1	indole	1V8Z wt. PLP
1YVB:R	Falcipain 2	peptide	3BPF wt. inhibitor
1ZLI:R	Carboxypeptidase B	lysine or arginine	1KWM wt. Zn cofactor
1ZM4:R	Elongation factor 2	GDP	2E1R wt. GDP
2AJF:R	ACE2	angiotensin	1R4L wt. inhibitor
2B42:L*	Xylanase	heteroxylan	2QZ3 wt. inhibitor
2B4J:R	HIV integrase core domain	-	-
2FD6:L	Plasminogen activator receptor	-	-
2H7V:R	Rac GTPase	GTP	1MH1 wt. GTP analog
1UDI:R	Uracil-DNA glycosylase	nucleotide	1UDH wt. uracil
2I9B:R	Plasminogen activator receptor	-	-
2NZ8:L*	Rac GTPase	GTP	1MH1 wt. GTP analog
2O8V:R	PAPS reductase	ADP, sulfite	-
2OOR:R	NAD(P) transhydrogenase	NADPH+NAD	1L7E wt. NAI
2OT3:L*	Rab21 GTPase	GTP	1YZU wt. GNP
2OUL:R	Falcipain 2	peptide	3BPF wt. inhibitor
2OZA:R	MAP kinase 14	ATP	3HEC wt. inhibitor
3D5S:L	Fibrinogen-binding protein	-	-
1E6J:L	HIV-1 capsid protein p24	-	-
2VIS:L	Flu virus hemagglutinin	-	-
1AKJ:R	MHC class 1 HLA-A2	-	-
1AK4:R	Cyclophilin	cyclosporin A	1CWB and 3RDD wt. inhibitor
1BUH:R	CDK2 kinase	ATP	2CCH wt. ATP analog
1F51:L	Sporulation response factor F	ATP	active site annotated in UNIPROT
1HE1:L*	Rac GTPase	GTP	1MH1 wt. GTP analog
1KAC:R	Adenovirus fiber knob protein	-	-
1KLU:R	MHC class 2 HLA-DR1	-	-

PDB code	Protein	Ligand	Structural data
**Huang**			
1BNC:B	biotin carboxylase	ATP, Biotin	3RUP wt. ADP
1DPG:A	glucose dehydrogenase	glucose, NADP	1H94 wt. NAD
1LEH:A	leucine dehydrogenase	leucine, NADP	1C1X (homolog) wt. NAD
1ORO:B	orotate phosphoribosyl transferase	orotidine, diphosphate	1LH0 (homolog) wt. orotate
1OSJ:A	3-isopropylmalate dehydrigenase	isopropylmalate, NAD	4WUO wt. NAD
1PKY:C	pyruvate kinase	pyruvate, ATP	active site annotated in UNIPROT
1SCU:E	succinyl-coA synthetase	ATP, succinate, CoA	1CQI wt. ADP and CoA
2EIP:A	pyrophosphatase	diphosphate	2AU6 wt. POP
8CAT:A	oxidoreductase	H2O2	3J7U wt. HEM and NAD
1HCG:A	coagulation factor X	arginine or threonine	4BTI wt. inhibitor
1TCO:A	calcineurin A - ser/thr phosphatase	serine or threonine, H2O	2IE4 (homolog) wt. okadaic acid
1TCR:B	T-cell receptor	-	-
1UBS:A	tryptophane synthase	indoleglycerol phosphate	4HT3 wt. inhibitor
8ATC:B	asp carbamoyl transferase regulatory chain	zinc ion	4FYW wt. inhibitor
9ATC:A	asp carbamoyl transferase catalytic chain	carbamoyl phosphate	2IPO wt. N-phosphonacetyl-L-asparagine
1APM:E	cAMP-dependent protein kinase	ATP	4XW4 wt. ATP analog
1UGH:E	uracil-DNA glycosylase	uracil	1EMJ wt. analog product

Proteins from PPDBv4 and Huang for which **SC2** was automatically chosen as the main scoring scheme and that do not contain a bound small molecule. For each protein, the PDB code, the name and/or function, the known ligand(s) and the structural data about complexed state(s) if any (PDB code, ligand present in the complex) are reported. Antibodies from PPDBv4 were excluded from this analysis as **SC3** was found to be the most appropriate scoring scheme for this class of proteins. The symbol "*" indicates structures that are wrongly annotated in PPDBv4 (mismatch between PDB code and name at `http://zlab.umassmed.edu/benchmark/`). The symbol "-" indicates that no evidence of a small molecule ligand could be found.
