# Supplementary material for: Local Geometry and Evolutionary Conservation of Protein Surfaces Reveal the Multiple Recognition Patches in Protein-Protein Interactions
Source: PLoS Comput Biol. 2015 Dec 21;11(12):e1004580. doi: 10.1371/journal.pcbi.1004580 (PMC4686965; doi:10.1371/journal.pcbi.1004580)
Supplement: S18 Table — (PDF) [file pcbi.1004580.s018.pdf]

PDB code	Alternative interface	Alternative PDB code
**Huang**		
1G3N:B	-	-
1LEH:A	interface with the natural ligand (active site)	1C1X (homolog)
1UGH:E	interface with FAM72A (Ugene)	-
**PPDBv4**		
1EAW:R	-	-
1MAH:R	homo-dimeric interface	1J06
1FC2:L	-	-
1GHQ:R	interface with another partner, complement factor H	4ONT
1I4D:R	interface with another partner, ADP-ribosylation factor-like protein 1	4DCN
1KLU:R	homo-dimeric interface	4FQX
1QA9:L	-	-
1SBB:L	interface with a second copy of the partner	1SBB
1FSK:L	-	-
1I2M:R	interface with another partner, Transportin-3	4OL0
1IJK:L	interface with another partner, Platelet glycoprotein Ib	1U0N
1AZS:R	homo-dimeric interface	1AB8
1E4K:L	-	-
1GPW:L	-	-
1K74:R	-	-
1MQ8:L	interface with another partner, Efalizumab	3EOB
1XD3:R	-	-
1XU1:R	interfaces with other partners	3K48, 1XU2
2B42:R	-	-
2B4J:R	interface with a second copy of the partner	2B4J
2CFH:L	-	-
2O3B:R	-	-
2OT3:L	-	-

Proteins from Huang and PPDBv4 for which JET^2 complete automated procedure yielded multi-patch predictions, obtained by combining different scoring schemes, that do not match experimental interfaces comprised of multiple recognition patches as defined by Janin and co-authors. For each protein, the PDB code is given, the alternative interface, if any, is described and the PDB code of the corresponding structure, if available, is given. The data were manually collected from the PDB and UNIPROT. The symbol ”-” indicates that no evidence of an alternative interface or no alternative PDB file could be found.
