# Supplementary material for: Local Geometry and Evolutionary Conservation of Protein Surfaces Reveal the Multiple Recognition Patches in Protein-Protein Interactions
Source: PLoS Comput Biol. 2015 Dec 21;11(12):e1004580. doi: 10.1371/journal.pcbi.1004580 (PMC4686965; doi:10.1371/journal.pcbi.1004580)
Supplement: S19 Table — (PDF) [file pcbi.1004580.s019.pdf]

PDB code	Alternative interface	Alternative PDB code
1D6R:L	interaction with a second copy of the partner	1D6R
1BUH:L	interaction with another partner, Skp1-Skp2	2ASS
1ACB:L	-	-
1GLA:L	homodimeric interface	1F3Z
1XU1:L	-	-
1Z5Y:L	-	-
2O8V:L	interactions with other partners, Trx reductase and T7 DNA polymerase	1F6M, 1X9M
2OOB:R	homodimeric interface	2OOB
2OOB:L	-	-
3SGQ:R	homodimeric interface	2QA9
BOYV:L	-	-

Proteins from PPDBv4 whose interaction site was predicted by **SC1** with high sensitivity (> 80%) but rather low precision (< 60%). For each protein, the PDB code is given, the alternative interface, if any, is described and the PDB code of the corresponding structure, if available, is given. The data were manually collected from the PDB. The symbol ”-” indicates that no evidence of an alternative interface could be found.
