# Supplementary figures and images for: Local Geometry and Evolutionary Conservation of Protein Surfaces Reveal the Multiple Recognition Patches in Protein-Protein Interactions
Source: PLoS Comput Biol. 2015 Dec 21;11(12):e1004580. doi: 10.1371/journal.pcbi.1004580 (PMC4686965; doi:10.1371/journal.pcbi.1004580)

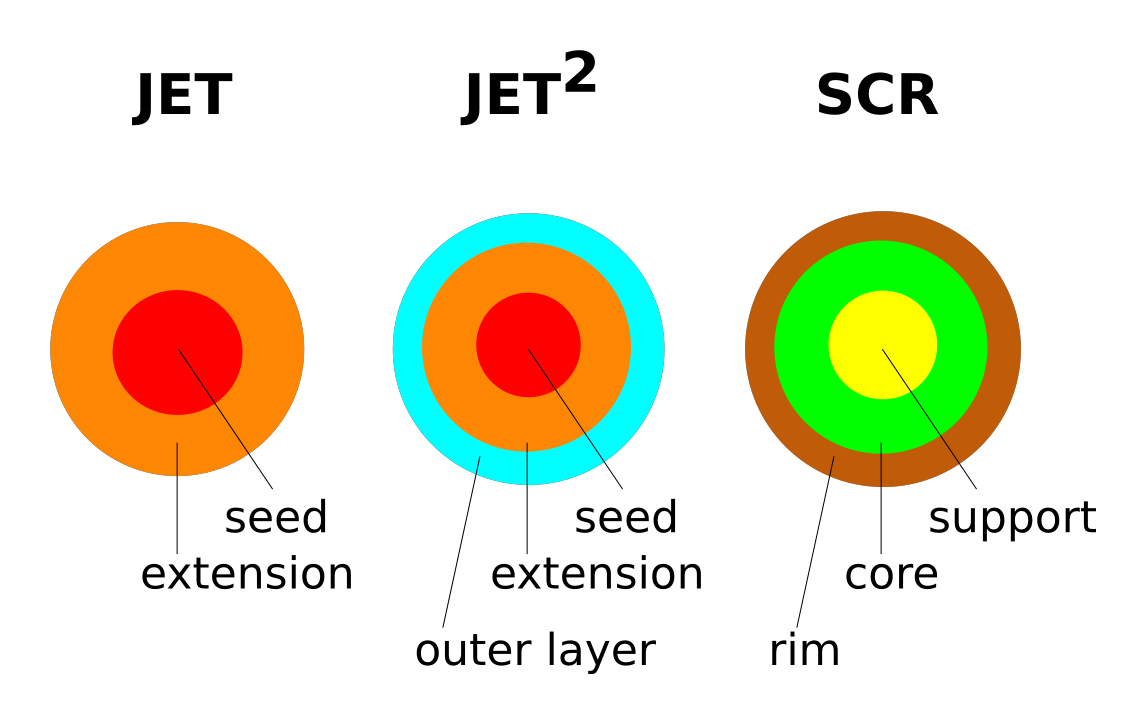

Supplement: S1 Fig — JET predictive model for protein binding sites comprises a cluster seed, detected based on conservation levels or physico-chemical properties, and an extension, detected by using a mixture of conservation and physico-chemical properties. JET2 predictive model comprises a seed, and extension and an outer layer. In scoring scheme SC1 taken here as an example, the seed is detected based on conservation levels, the extension is defined from conservation and physico-chemical properties, and the determination of the outer layer accounts for physico-chemical properties and surface geometry. The SCR model [24] of experimental protein interfaces comprises support residues, that are buried, core residues, that become buried upon the formation of the complex, and rim residues, that are exposed. (TIFF) [file pcbi.1004580.s020.tiff]

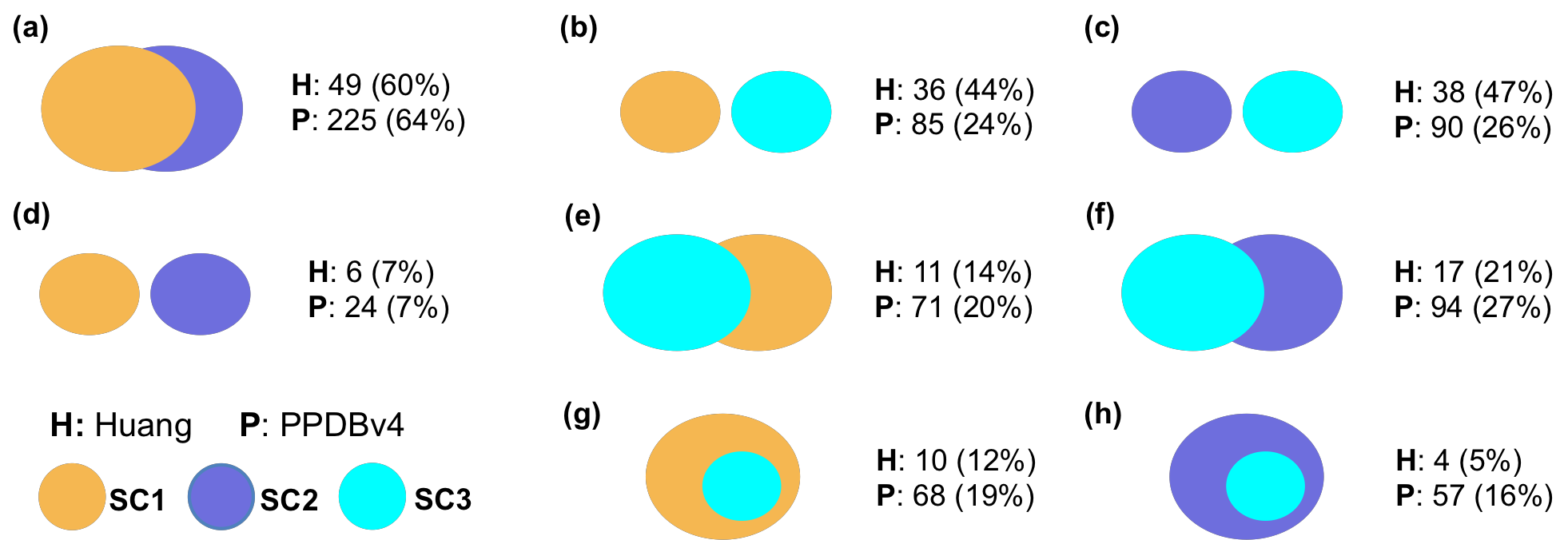

Supplement: S2 Fig — The predictions were obtained from a consensus of 2 runs out of 10 of iJET2. Overlaps are computed as: o i,j = #(pred i ∩ pred j)/#(pred i), where pred i and pred j represent the residues predicted at the interface by SCi and SCj respectively. (a) o 1,2 > 50% & o 2,1 > 50%, (b) o 1,3 < 10% & o 3,1 < 10%, (c) o 2,3 < 10% & o 3,2 < 10%, (d) o 1,2 < 10% & o 2,1 < 10%, (e) 10%≤o 1,3 < 60% & 10%≤o 3,1 < 60%, (f) 10%≤o 2,3 < 60% & 10%≤o 3,2 < 60%, (g) o 3,1 > 90% & o 1,3 < 80%, (h) o 3,2 > 90% & o 2,3 < 80%. The proportions of proteins from the Huang dataset (H) and PPDBv4 (P) corresponding to each case are given. (TIFF) [file pcbi.1004580.s021.tiff]

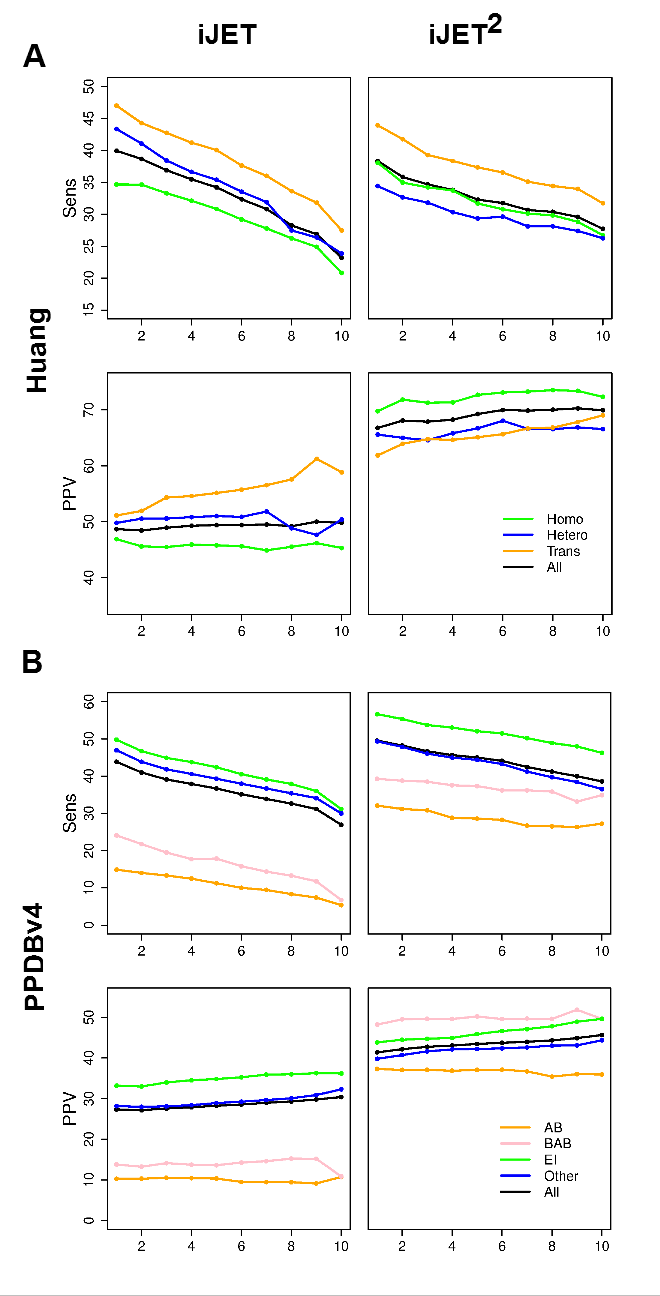

Supplement: S3 Fig — Average sensitivity and precision were computed on (A) the homodimers (in green), heterodimers (in blue), transients (in orange) and all proteins (in black) from Huang; (B) the antibodies-antigens (in orange), bound antibodies-antigens (in pink), enzymes-inhibitors (in green), others (in blue) and all proteins (in black) from PPDBv4. Predictions were obtained by a consensus over 10 runs (x-axis). (TIFF) [file pcbi.1004580.s022.tiff]

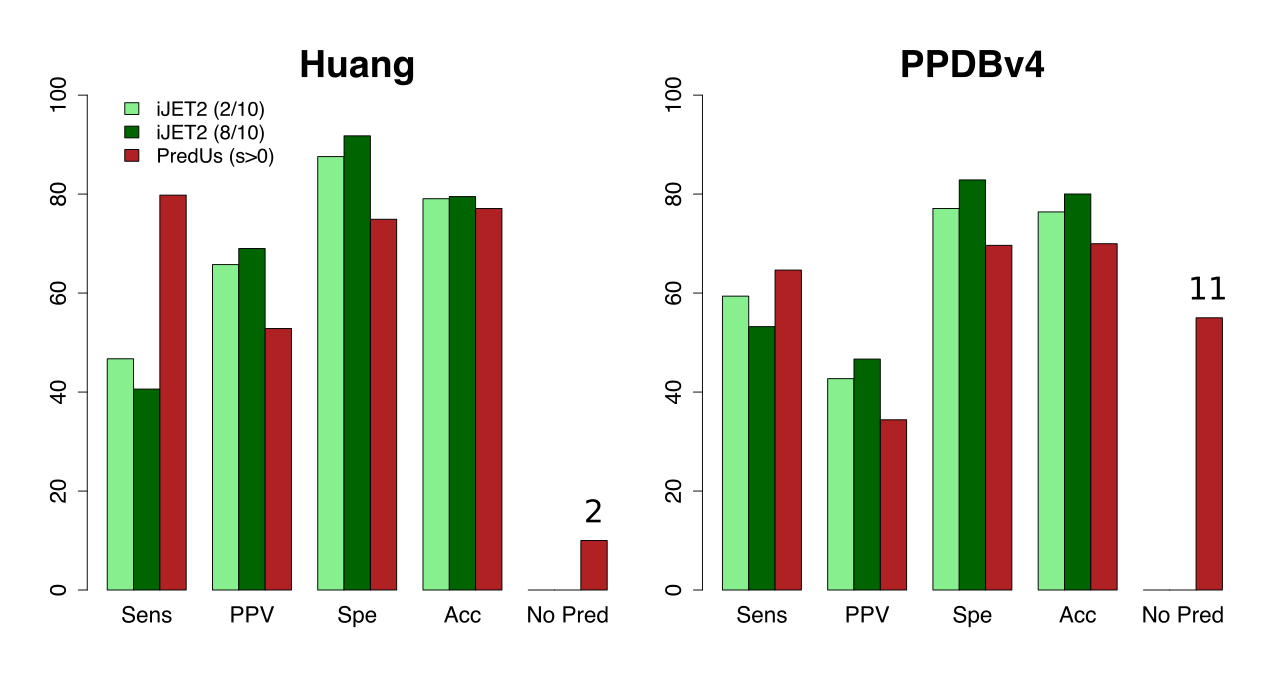

Supplement: S4 Fig — Average sensitivity (Sens), positive predictive value (PPV), specificity (Spe) and accuracy (Acc) are plotted for proteins from the Huang dataset (on the left) and PPDBv4 (on the right). Huang: 2 proteins for which PredUs calculation failed were excluded; PPDBv4: the proteins used for training PredUs, the multi-chain proteins and the 11 proteins for which PredUs calculation failed were excluded, resulting in a subset of 80 proteins. The numbers of proteins that could not be treated are indicated on the right (“No Pred”). For iJET2, consensus predictions were obtained from 2 (in light green) and 8 (in forest green) runs out of 10. The clustering procedure was run using all three scoring schemes for each protein and the best patch or combination of patches was retained for performance assessment. For PredUs, predicted patches were defined as formed by residues with positive scores (in firebrick). (TIFF) [file pcbi.1004580.s023.tiff]

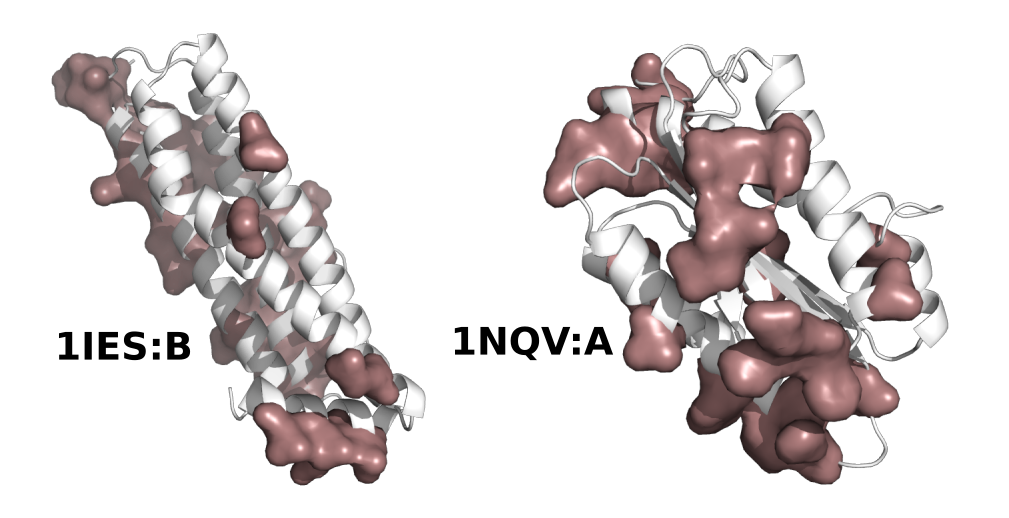

Supplement: S5 Fig — The predicted residues are displayed in dark pink surface. The predictions were defined from the list of confidently predicted interfacial residues determined by eFindSitePPI web server (compare to Fig 6). (TIFF) [file pcbi.1004580.s024.tiff]

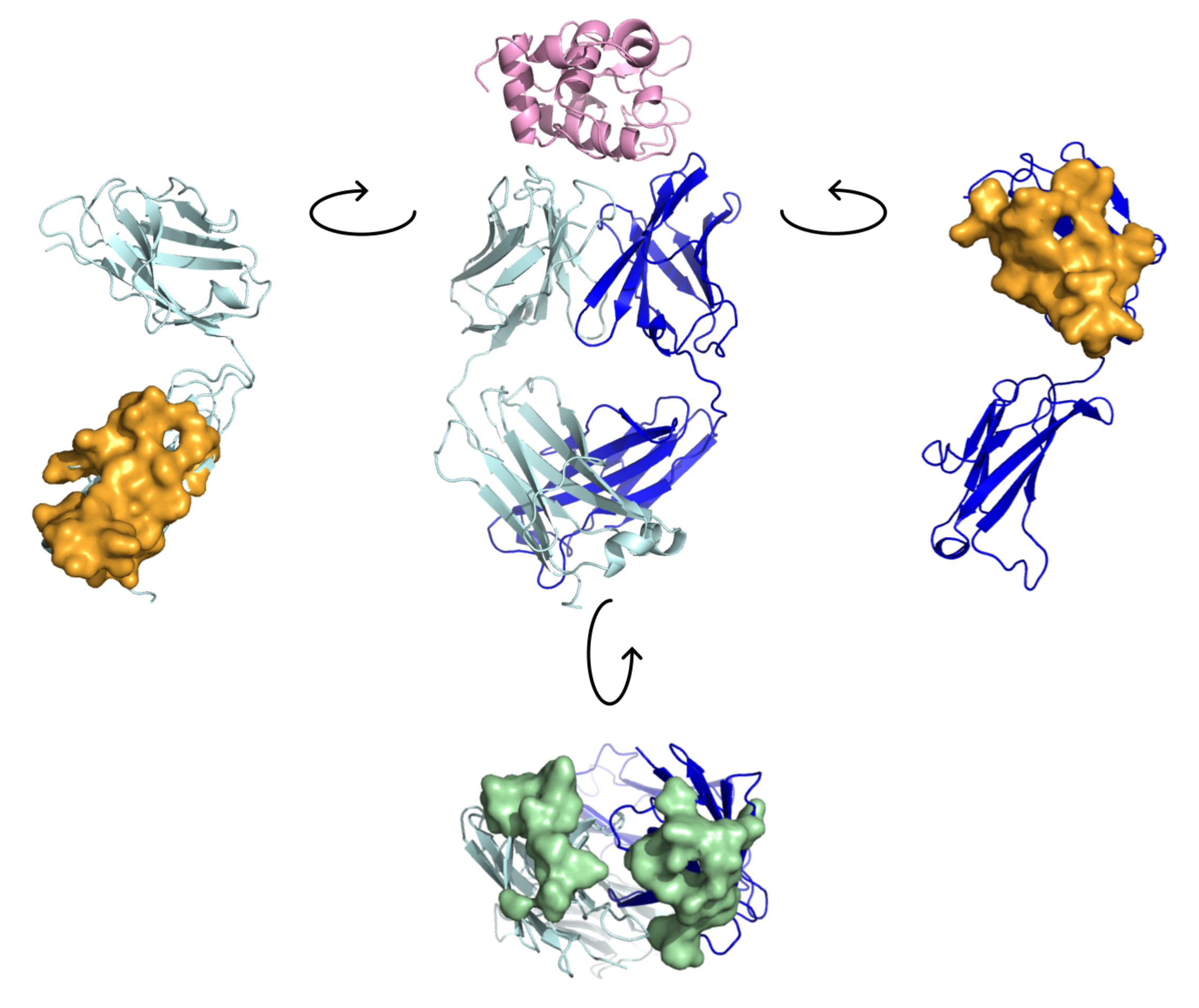

Supplement: S6 Fig — The antibody’s heavy and light chains are represented as marine and pale cyan cartoons while lysozyme is in pink. The patches predicted by JET2 for the antibody are depicted as opaque surfaces and colored according to the scoring scheme used: SC1 in orange, SC3 in green. (TIFF) [file pcbi.1004580.s025.tiff]

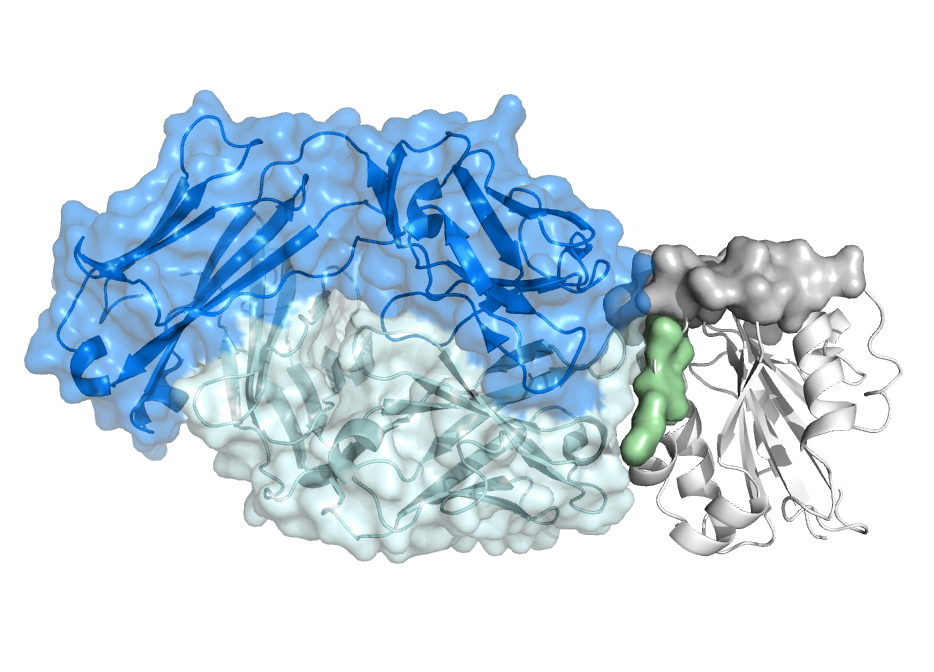

Supplement: S7 Fig — LFA-1 is displayed as a grey cartoon. The heavy and light chains of Efalizumab are colored in marine and palecyan and displayed as cartoon and transparent surface. The experimental binding site of LFA-1 natural ligand is depicted as opaque grey surface. The five residues detected by iJET2 SC3 that are specific to the interaction with Efalizumab are depicted as green opaque surface. (TIFF) [file pcbi.1004580.s026.tiff]

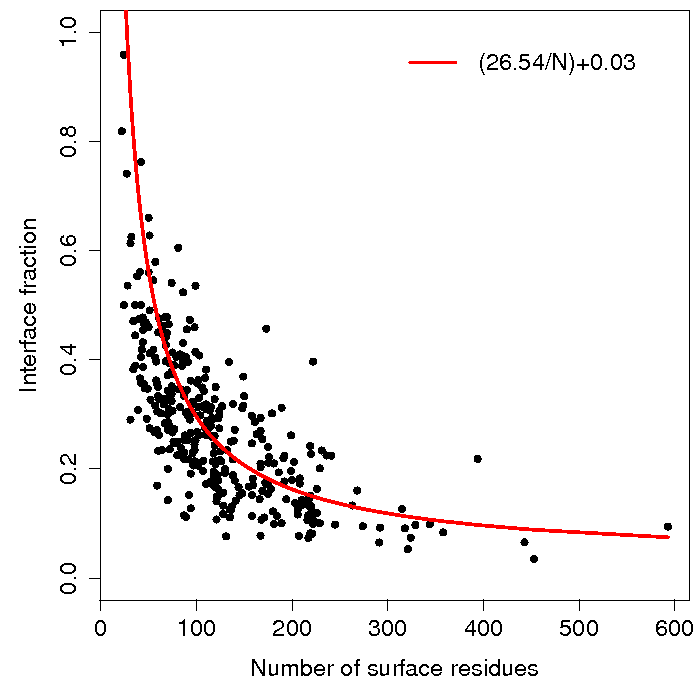

Supplement: S8 Fig — The fraction of the surface covered by the interface (y-axis) is plotted versus the number of surface residues (x-axis) for all 352 proteins from PPDBv4. The red line corresponds to the f intfrac(N) function. (TIFF) [file pcbi.1004580.s027.tiff]
